# The paleoecology and taphonomy of a Santonian-Campanian (Upper Cretaceous) dinosaur-bearing vertebrate locality from Bulgaria: a window into an underexplored part of the Late Cretaceous European Archipelago

**DOI:** 10.1186/s13358-025-00388-z

**Published:** 2025-07-29

**Authors:** Vladimir Nikolov, Polina Pavlishina, Docho Dochev, Stephen L. Brusatte

**Affiliations:** 1 National Museum of Natural History at the Bulgarian Academy of Sciences, 1 Tsar Osvoboditel Blvd., 1000 Sofia, Bulgaria; 2 Department of Geology, Paleontology, and Fossil Fuels, Sofia University ‘St. Kliment Ohridski’, 15 Tsar Osvoboditel Blvd., 1504 Sofia, Bulgaria; 3 School of GeoSciences, University of Edinburgh, Grant Institute, James Hutton Road, Edinburgh, EH9 3FE UK

**Keywords:** Palynology, Palynofacies analysis, Normapolles, Vertebrate assemblage, Bone histology, Wedl tunnel, Bioerosion, Rezhantsi Formation

## Abstract

The Upper Cretaceous European vertebrate fossil record has improved significantly in the past three decades but there still remain chronostratigraphic and geographic gaps, which obscure our understanding of the paleobiogeography and evolution within the insular environments of the Late Cretaceous European Archipelago. Recently, a new vertebrate locality of late Santonian–early Campanian age was discovered in westernmost Bulgaria, promising to fill some of these gaps. Here, we use a multidisciplinary approach involving palynology, paleontology and paleohistology to investigate aspects of the paleoecology and taphonomy of this new locality and to provide preliminary information on its taxonomic contents. Palynomorph data shows that the flora was dominated by angiosperms of the Normapolles group with subordinate presence of ferns and only rare gymnosperms. The association of the pollen taxa *Krutzschipollis crassus* and *K. spatiosus* supports latest Santonian to early Campanian age for the vertebrate-bearing strata. The floral composition and especially a number of fern spore humidity indicators imply the existence of a generally humid subtropical climate, with some seasonal droughts. Using palynofacies analysis, we infer a coastal, proximal shelf to oxidated deltaic or lagoonal depositional environment for the examined sedimentary succession. The locality has so far yielded 250 vertebrate specimens collected from eight strata. There are at least seven clades present, including lamniform sharks, lepisosteid gars, amphibians, turtles, crocodylomorphs, ornithopod and titanosaur dinosaurs, and possibly pterosaurs. Semi-aquatic and aquatic animals dominate the assemblage. Most common are turtles (about 30% of the sample), followed by dinosaurs. Skeletal elements are disarticulated, isolated and mostly fragmentary. Fossils are not sorted by size. Many of the fossil bones show signs of abrasion and bioerosion, both micro- and macroscopically. Paleohistological data reveal that all sampled dinosaur bones belong to subadult or adult individuals. We interpret the site to be an attritional assemblage. Taxonomic comparisons with other well-known Santonian to Maastrichtian fossil assemblages from Central (Hungary and Austria) and Eastern Europe (Romania and Serbia) indicate similarities with both the Santonian Iharkút-Ajka vertebrate fauna of Hungary and the younger Haţeg Island fauna of Romania, although additional material and more precise taxonomic identification of the Bulgarian fossils is needed. Our work presents the first more in-depth look at life on land in this currently underexplored part of the Late Cretaceous European Archipelago.

**Supplementary Information:**

The online version contains supplementary material available at 10.1186/s13358-025-00388-z.

## Introduction

The European fossil record provides a unique perspective on the evolution of terrestrial vertebrate faunas during the Late Cretaceous and their response to the major climatic and environmental changes. This uniqueness reflects the geological past of the continent: for the duration of the Late Cretaceous, modern-day Europe was a vast archipelago with ever changing topography and geography (Dercourt et al., 2000; van Hinsbergen et al., 2020). Consequently, the vertebrates living on the islands were shaped by complex processes of dispersal, isolation, and insular evolution (e.g., Benton et al., 2010; Csiki-Sava et al., 2015; Le Loeuff, 1991; Pereda-Suberbiola, 2009; Weishampel et al., 2010). Despite important paleontological work conducted in the last decades in different parts of Europe (Allain & Pereda Suberbiola, 2003; Buffetaut et al., 1997; Codrea et al., 2010; Dal Sasso, 2003; Grigorescu, 2010; Ortega et al., 2015; Pereda-Suberbiola et al., 2015), our understanding of the interplay between these processes and how they shaped the archipelago’s vertebrate faunas remains obscured by the notably patchy and stratigraphically interrupted nature of the fossil record (Buffetaut & Le Loeuff, 1991; Csiki-Sava et al., 2015). This holds particularly true for the pre-late Campanian times – a period of relatively high sea levels and little exposed land area (Dercourt et al., 2000; Haq, 2014; Miller et al., 2003)—which are crucial for understanding how the better known late Campanian–Maastrichtian dinosaur-dominated terrestrial island communities came to be. Therefore, each new Upper Cretaceous European vertebrate locality yielding remains of terrestrial fauna, especially if it is from currently understudied regions, is of high interest and promises to expand our knowledge on life on the European Archipelago before the end-Cretaceous mass extinction (Csiki-Sava et al., 2015; and references therein).

Bulgaria has a notoriously scarce fossil record of Mesozoic tetrapods (Boev, 2017), with the first remains of terrestrial vertebrates, two non-avian dinosaurs of late Maastrichtian age, reported only recently from the Labirinta Cave and adjacent area, near the town of Cherven Bryag, NW Bulgaria (Godefroit & Motchurova-Dekova, 2010; Mateus et al., 2010). Systematic prospecting, initiated by the find of a single fossil bone fragment in 2006, has led to the discovery of a new Upper Cretaceous vertebrate fossil locality in the area of the Vrabchov Dol gully, near the town of Tran (Western Bulgaria) (Nikolov et al., 2020b). Subsequent field work at the locality (2018–2024) revealed an unexpected taxonomic diversity of Mesozoic vertebrates, from terrestrial to semi-aquatic and aquatic taxa, the highest thus far reported from the country. Currently described fossil material pertains to lepisosteids (Nikolov et al., 2025), allodaposuchids (Hristova, 2020) and a putative titanosaur (Nikolov et al., 2020b), but preliminary data indicates the presence of turtles, microvertebrates (amphibians), ornithischian dinosaurs, and possibly pterosaurs (Nikolov et al., 2020a). Using palynological data derived from three beds within the sedimentary succession at the site, Pavlishina et al. (2019) dated the fossil-bearing deposits to the late Santonian–early Campanian and revealed that the area inhabited by the fauna was dominated by a Normapolles group angiosperm paleoflora and experienced a seasonally dry, warm paleoclimate.

**Fig. 1:**
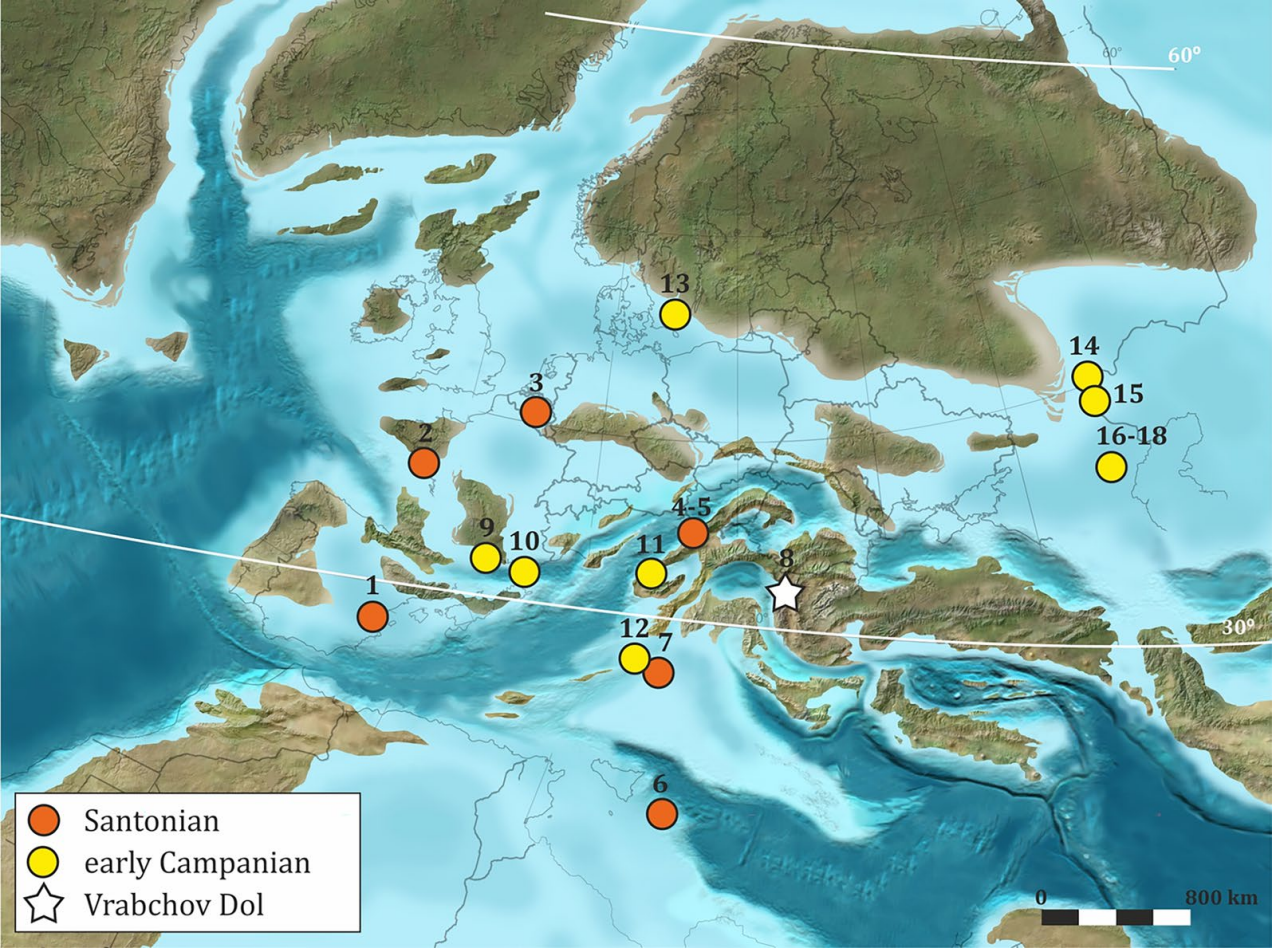
Paleogeographic distribution of Santonian and lower Campanian vertebrate fossil localities in Europe. **1** Millares, Valencia Province, Spain; **2** Notre-Dame-de-Riez, Vendée, France; **3** Lonzée, Namur Province, Belgium; **4** Iharkút, Hungary; **5** Ajka, Hungary; **6** Altamura, Bari, Italy; **7** Križ, Kras, Slovenia; **8** Vrabchov Dol, Pernik Province, Bulgaria; **9** Villeveyrac, Hérault Department, France; **10** Lambeau de Beausset, Bouches-du-Rhône, France; **11** Muthmannsdorf, Austria; **12** Villaggio del Pescatore, Trieste Province, Italy; **13** Skåne Province, Sweden; **14** Malyy Prolom, Ryazan Oblast, Russia; **15** Malaya Serdova, Penza Province, Russia; **16** Beloe Ozero, Saratov Province, Russia; **17** Shyrokii karamysh, Saratov Province, Russia; **18** Saratov 2, Saratov Province, Russia. Base map for the Campanian (~ 75 Ma) by Ron C. Blakey, ©2023 Colorado Plateau Geosystems Inc. (used with permission)

The age of the Vrabchov Dol locality makes it of particular paleontological interest. Only a handful of Santonian and lower Campanian European localities yield fossils of terrestrial (and/or freshwater) vertebrates (Fig. 1). With few exceptions, like the more complete and diagnostic fossils from the famous Iharkút locality in Hungary (Ősi et al., 2012b), the vertebrate remains are typically very fragmentary and, therefore, poorly diagnostic at a genus, let alone at a species level. Santonian material is known from Belgium (Godefroit & Lambert, 2007), France (Buffetaut & Pouit, 1994), Hungary (Ősi et al., 2012b, 2016), Italy (Nicosia et al., 1999a, b; Dal Sasso, 2003), Slovenia (Buffetaut et al., 2002b), and Spain (Santisteban & Suñer, 2003), and lower Campanian fossils are found in Austria (Bunzel, 1871; Seeley, 1881), Italy (Chiarenza et al., 2021), France (Allain & Pereda Suberbiola, 2003; Buffetaut et al., 1996; Garcia & Pereda Suberbiola, 2003; Le Loeuff & Buffetaut, 1991), the European part of Russia (Averianov, 2007, 2014; Solonin et al., 2021), and Sweden (Lindgren et al., 2007; Weishampel et al., 2004). Among the most important of these, due to their taxonomic richness and state of detailed research, are the upper Santonian Iharkút fossil site in Western Hungary (Botfalvai et al., 2021b; Ősi et al., 2012b) and the lower Campanian Muthmannsdorf locality in Eastern Austria (Bunzel, 1871; Buffetaut, 1979; Pereda-Suberbiola & Galton, 1992, 2001; Sachs & Hornung, 2006; Seeley, 1881; Wellnhofer, 1980). Meanwhile, detailed paleoecological and taphonomic studies of European Upper Cretaceous localities yielding remains of terrestrial vertebrates are still relatively rare (Augustin et al., 2019; Botfalvai et al., 2015, 2016, 2017; Cincotta et al., 2015; Csiki et al., 2008, 2010b; Grigorescu, 1983; Pereda-Suberbiola et al., 2000; Therrien, 2005).

In this contribution, we investigate aspects of the paleoecology and taphonomy of the upper Santonian–lower Campanian vertebrate assemblage at Vrabchov Dol from palynological and paleontological perspectives. First, we aim to provide a generalized picture of the paleoenvironment inhabited by the Vrabchov Dol vertebrate fauna and to track, if possible, any environmental, depositional, and/or climatic changes. To do so, we build upon the previous research of Pavlishina et al. (2019) by performing a detailed bed-by-bed palynological and palynofacies analysis of the locality. The data from this analysis are then used to reconstruct the depositional environment for each bed, the composition of the contemporary paleoflora and the abundance of each of its components, and to derive information on the climate. Second, we analyse the fossils from the site, with specific focus on describing the nature of the vertebrate fauna. The taxonomic and skeletal element composition and abundance of fossils, and their state of preservation and stratigraphic distributions, are evaluated on the basis of an exhaustive list of the fossil material collected up until 2024. We use the data to determine the autochthonous and allochthonous components of the assemblage and reveal some of its taphonomic characteristics. Additionally, we use bone histology data to elucidate the ontogenetic structure of some of the non-avian dinosaur material and examine the characteristics of microscopic focal destruction (MFD) present in some of the studied specimens, in order to obtain more specifics about their taphonomic history. Several types of bioerosion traces present on some dinosaur bones provide further information on their taphonomy. Lastly, the palynological content and paleoflora, the vertebrate fauna, and the paleoenvironment of the Vrabchov Dol locality are compared in detail to those of the similarly-aged European fossil localities, particularly Iharkút and Ajka (Hungary) and Muthmannsdorf (Austria) localities. Brief paleoenvironmental and taxonomic comparisons of the Vrabchov Dol assemblage to the recently discovered vertebrate faunas of late Turonian and early Coniacian ages in Austria (Ősi et al., 2019b, 2021) and from the Maastrichtian of Serbia (Marković et al., 2025), as well as to the classic Haţeg fauna from the latest Cretaceous of Romania, are also made.

## Geological background

**Fig. 2:**
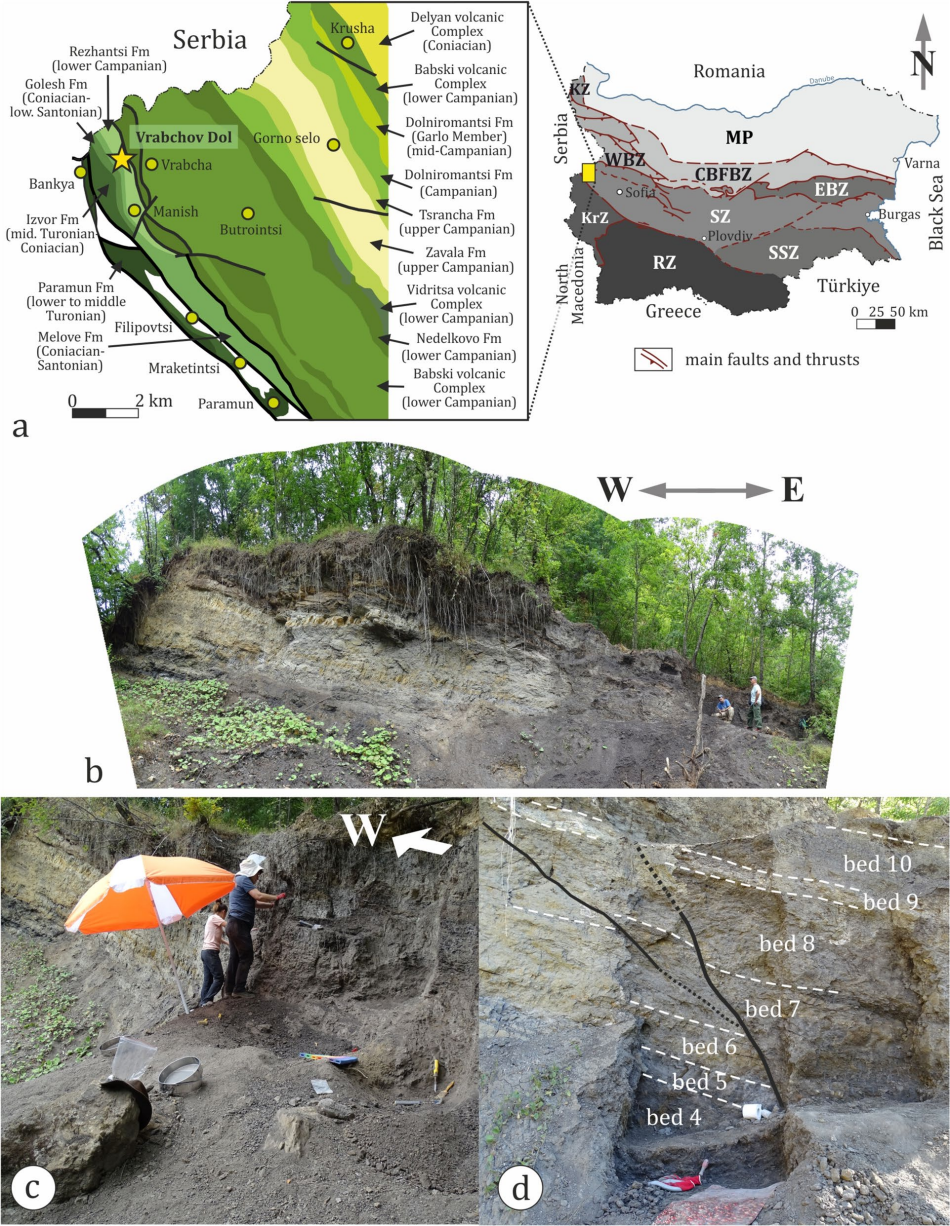
Geographic position and geology of the Vrabchov Dol locality. **a** Tectonic division of Bulgaria and geological sketch map of the area of Vrabchov Dol. *CBFBZ* Central Balkan–Fore-Balkan Zone, *EBZ* East Balkan Zone, *KZ* Kula Zone, *KrZ* Kraishte Zone, *MP* Moesian Platform, *SZ* Srednogorie Zone, *SSZ* Sakar-Strandzha Zone, *RZ* Rhodope Zone, *WBZ* West Balkan Zone. **b** Panoramic view of the vertebrate locality as of August 2023. **c** The naturally formed ramp at the level of most productive fossiliferous strata in the eastern part of the site as of August 2024. **d** Part of the sedimentary section in the central parts of the site affected by small faults (black solid and dotted lines)

The Vrabchov Dol fossil vertebrate locality is situated in a small west-to-east elongated gully between the villages of Bankya and Vrabcha, about 4.7 km NE of the town of Tran (Tran municipality, NW Bulgaria). In geological terms, it falls into the Western Srednogorie Tectonic Subzone (Ivanov, 2017) (Fig. 2). The Upper Cretaceous rocks exposed in the subzone form northwest-southeast strips and are mainly composed of diverse carbonates and terrigenous lithologies, as well as small intrusive bodies and large amounts of volcano-sedimentary successions. The Upper Cretaceous strata crop out throughout the entire Vrabchov Dol gully, whereas the pre-Upper Cretaceous basement has a strictly limited distribution. It is represented mostly by the diverse organogenic and bioclastic limestones of the Slivnitsa Formation (upper Kimmeridgian-Valanginian) (at the vicinity of Bankya village) and the Para-Urgonian Unit (lower Barremian-Aptian) (exposed southwest from the Vrabcha village) (Marinova et al., 2010).

The knowledge of lithostratigraphy, chronostratigraphy, distribution and evolution of the Upper Cretaceous deposits in the Western Srednogorie Subzone has undergone many changes (see Nikolov et al., 2020b). The currently accepted and used lithostratigraphical scheme and chronostratigraphical frame is that of Sinnyovsky et al., (2012, 2013). These authors propose and define 12 formal formations and members. Based on the chronostratigraphic distribution and biostratigraphy of the key nannofossils markers, the chronostratigraphical interval of the Upper Cretaceous rocks exposed at the Vrabchov Dol gully spans the lower Turonian to the upper Campanian (Sinnyovsky et al., 2012, 2013).

Geological field work conducted in the period 2018–2024 by one of us (D.D.) along the Vrabchov Dol gully allows for a tentative reconstruction of the sedimentation history in the studied area. During the Late Cretaceous, the sedimentation took place in relatively shallow marine paleoenvironments as the sea level was gradually rising, with short term fluctuations, in a basin with dextral transitional tectonic regime (Dochev, pers. obs.). Characteristic for this type of basin environment are fast lateral and vertical lithological transitions. During the early-middle Turonian times, the marine transgression started with a flooding of the paleorelief, formed by the erosion of Slivnitsa Formation and Para-Urgonian Unit limestones. During this time, the deposition of terrigenous material, represented by conglomerates, sandstones and siltstones with rare strata of claystones, sandy marlstones and limestones, led to the formation of the Paramun Formation (Sinnyovsky et al., 2012). Later, the thin-bedded marlstones and limestones of the Izvor Formation (middle Turonian–lower Coniacian) and the gray, red and motley sandy-clayey marlstones and slightly cemented clayey sandstones of the Golesh Formation (Coniacian–Santonian) were accumulated under a regime of continuing sea level rise. The very distinctive white and motley thin-bedded carbonate turbidites, with rare thin-bedded marlstones and sandy limestones, of the Melove Formation (Santonian–lower Campanian) were deposited in slope environments, forming a slope carbonate turbidite apron. During late Santonian–early Campanian times, the marine paleonvironments became shallower, and the diverse argillites, sandstones, marlstones, sandy limestones and reef limestones of Rezhantsi Formation were accumulated. Palynological data reveals that these diverse sedimentary rocks were settled in very shallow, foreshore to lagoonal paleoenvironments (Pavlishina et al., 2019).

**Fig. 3:**
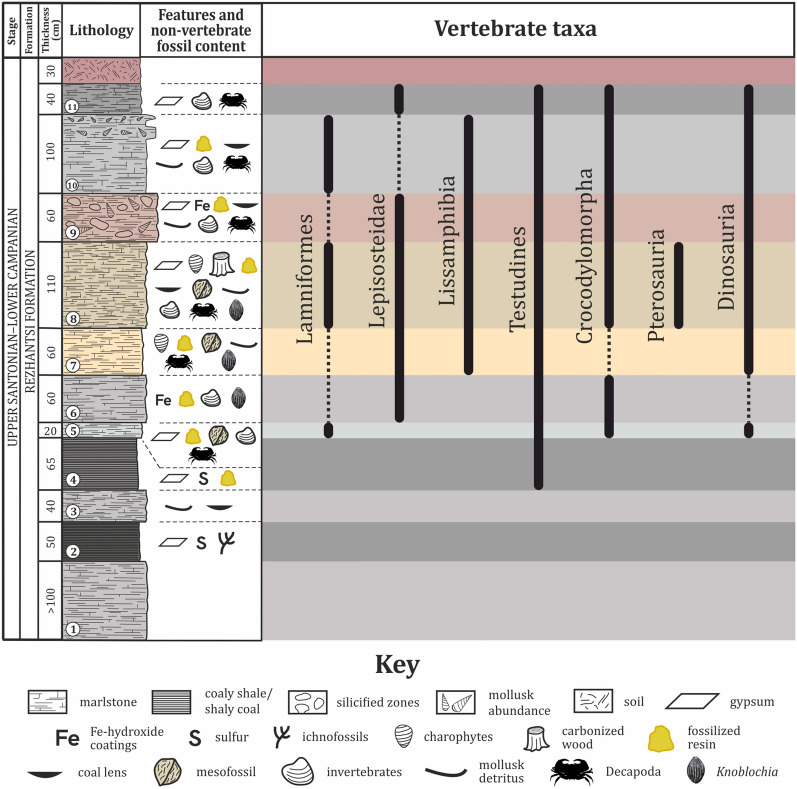
Lithology of the Vrabchov Dol vertebrate locality and stratigraphic distribution of recognized vertebrate taxa. Decapod silhouette by T. Michael Keesey (Public Domain Mark 1.0; www.phylopic.org)

The studied fossil-bearing section is located about 1 km south-west from the village of Vrabcha and falls into the range of the Rezhantsi Formation (Nikolov et al., 2020b; Pavlishina et al., 2019) (Fig. 2a). The section represents a small slumped block of sedimentary strata with unclear relationships with overlying and underlying rocks due to soil and dense vegetation cover. Rocks crop out as a vertical wall, which is generally west–east oriented and has a width of about 20–25 m. The strata have a total thickness of ~ 8 m, but the thickness of the beds is not laterally uniform across the outcrop. There appears to be a westward trend of increase in stratum thickness in the upper half of the section (Fig. 2b). In the eastern part of the outcrop, there is a naturally formed ramp, which allows access to the upper third of the rock section (Fig. 2c). Several small-scale faults are observed mostly in the central parts of the outcrop (Fig. 2d). Lithologies present at the site include variously colored but mostly gray to light-gray marls, silicified marls, coal shales, shaly coals and coal lenses. These rocks are grouped into 11 beds (strata) of which eight yield remains of fossil vertebrates (Fig. 3).

## Material and methods

### Palynological sampling and analyses

**Fig. 4:**
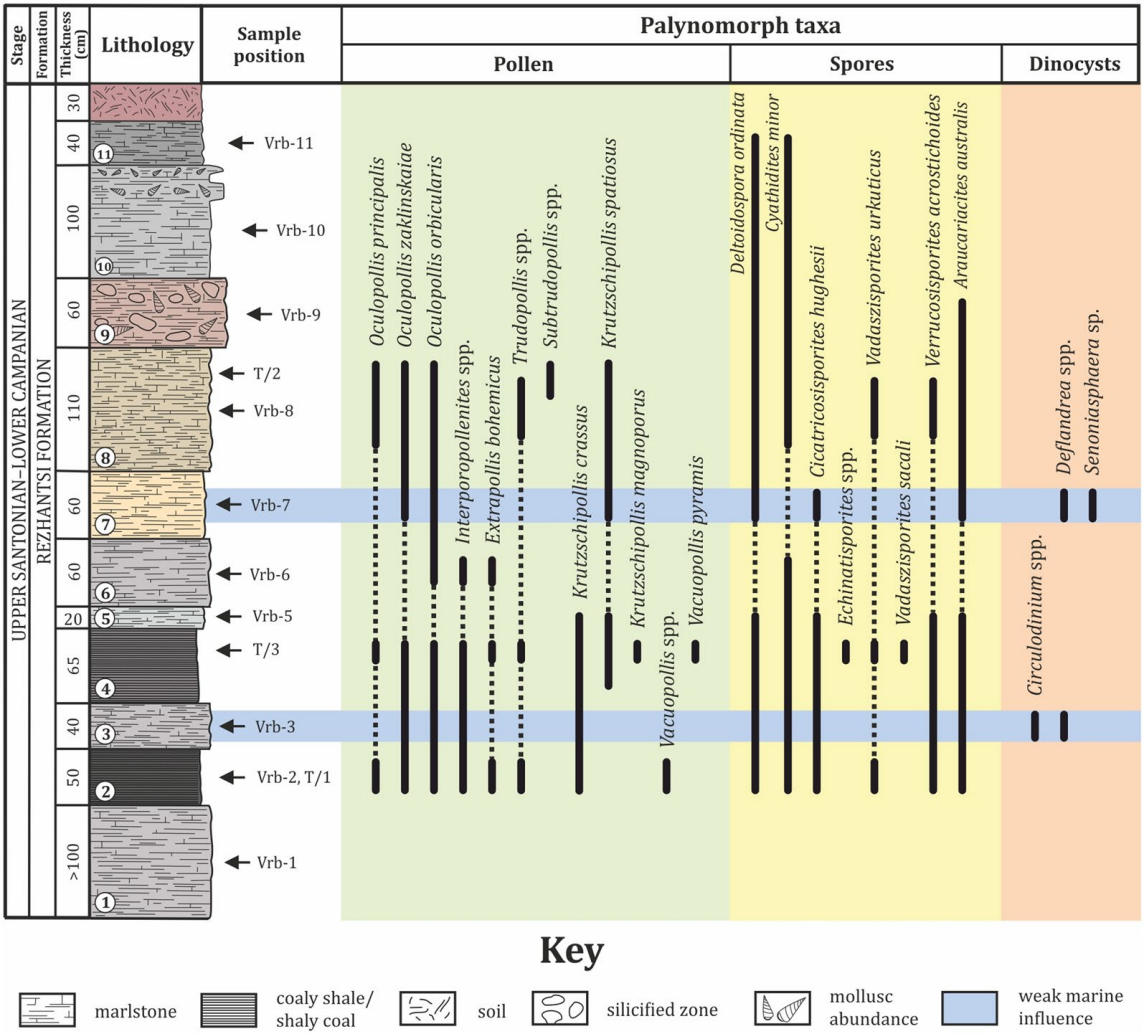
Stratigraphic position of palynological samples and distribution of palynomorphs

Each bed of the vertebrate-bearing succession at Vrabchov Dol, with the sole exception of bed 4, was sampled for palynological analyses. During the sampling process, we followed the lithological division of the deposits established by Pavlishina et al. (2019) and Nikolov et al. (2020b). Samples were collected from fresh rock surfaces at about mid-levels within each bed (Fig. 4). All samples were designated with the abbreviation ‘Vrb’ (for the Vrabchov Dol gully), followed by the number of the bed which was sampled (e.g., sample ‘Vrb-1’ is from bed 1, the lowermost in the succession), which was later carried over as a catalogue number for the respective palynological slides.

Palynological samples were processed at the Sample Preparation Laboratory (GeoPrep) of the Institute of Geological Sciences, Polish Academy of Sciences (Instytut Nauk Geologicznych, PAN), Kraków, Poland by Assist. Prof. Przemysław Gedl, following standard palynological techniques (Wood et al., 1996). About 50 g of sediment from each sample were processed by HCl (hydrochloric acid) and HF (hydrofluoric acid) treatment and heavy liquid separation. The residues were finally sieved through 10 µm nylon meshes. Strew mounts were made in glycerine jelly. From each sample, two palynological strew mounts were prepared and analyzed for their palynological content on a Leica DM5500 microscope. Selected palynomorphs were photographed with a microscope-mounted Leica DFC310 FX digital camera. Additionally, the three samples studied by Pavlishina et al. (2019), which were taken from beds 2, 4, and 8, were re-examined for the purposes of this study (see Fig. 4 for the catalogue numbers of these slides). The palynological slides are stored in the collections of the Department of Geology, Paleontology and Fossil Fuels at Sofia University ‘St. Kliment Ohridski’.

Besides the study of their palynological content, the slides were subjected to a palynofacies analysis. The analysis focuses on the composition and major characteristics of the sedimentary organic matter (OM) constituents based on counting 400 particles per slide. Three main groups of kerogen constituents proposed by Tyson (1995), Radmacher et al. (2020) and Slimani et al. (2021) have been recognized in the slides, namely: (1) phytoclasts (translucent and opaque organic particles), (2) palynomorphs (spores, pollen and dinoflagellate cysts) and (3) amorphous organic matter (AOM). The data are plotted in the ternary AOM–Phytoclast–Palynomorph plot of Tyson (1993). Palynofacies parameters, such as the ratio of continental to marine particles (C/M ratio) and the ratio of opaque to translucent phytoclasts (OP/TR ratio) (Tyson, 1993, 1995), are estimated to characterize the paleoenvironmental settings.

### Fossil material—overview

All fossil material collected during the period 2017–2024 was examined for the purposes of this study. Although the fossil site yields diverse plant (charophytes, mesofossils, carbonized wood, and amber) and invertebrate fossils (gastropods, bivalves, decapods, as well as insect eggs), herein we focus only on the vertebrate remains, with non-vertebrate material mentioned only briefly and discussed where it can provide additional paleoecological and/or taphonomic information. A list of 250 vertebrate specimens was compiled, including information (where available) on each specimen’s nature (i.e. type of skeletal element), taxonomic affinities, stratigraphic position, date of collection and collector. All information is presented in Table S1. Except for one specimen, all Vrabchov Dol fossil remains are housed in the Paleontological collection of the National Museum of Natural History at the Bulgarian Academy of Sciences (NMNHS). Specimen U.S., K_2_ 1586, described by Nikolov et al. (2020b), is curated at the Museum of Paleontology and Historical Geology, Sofia University ‘St. Kliment Ohridski’ (MPHG).

### Vertebrate macrofauna

Vertebrate macrofossils were excavated following standard vertebrate paleontology practices. A few severely fractured and fragmented specimens, mostly partial turtle carapaces, but also dinosaur long bones, were collected in plaster jackets and then prepared in the paleontological laboratory of the NMNHS. Because of the steep topography of the fossil locality, it was not possible to use a square-meter grid and to create a sitemap to document the exact position and orientation of each fossil in regards to other fossils within the same bed. Available field notes have been examined for the purposes of elucidating some aspects of the taphonomy of the vertebrate remains. Bones were studied for the presence of features indicative of weathering, bioerosion, transportation and mechanical abrasion. Preliminary taxonomic identification of the macrofauna fossils was done largely with reference to published literature. The non-avian dinosaur material was further studied and compared to the Maastrichtian-aged titanosaur and ornithopod fossils in the Paleontological collection at the University of Bucharest, Romania. For our analysis, all taxonomically undiagnostic and/or currently indeterminate specimens of vertebrate macrofauna (referred in Table S1 to the clades Ornithodira, Archosauria, Archelosauria, Sauropsida, or Tetrapoda) were lumped in the category ‘indet. macrofauna’. Although on several occasions we provide comments on the morphology and taxonomy of part of the studied material, detailed osteological description and taxonomic interpretation of the fossils is beyond the scope of this paper and will be published elsewhere. For description of the macroscopic bioerosion traces (i.e. general morphology, site of emplacements and pattern of occurrence) observed on some bones, we follow the ichnotaxabases scheme proposed by Pirrone et al. (2014). Specimens were photographed with a Sony HX400V digital camera and microphotographs were taken under Zeiss Stemi 2000-C stereomicroscope with mounted Canon EOS 1300D digital camera.

### Vertebrate microfauna

Although some specimens have been collected in situ in the field, the majority of the microvertebrate material discussed in this study was collected via screenwashing. During the 2021 fieldwork season, between 2 and 5 kg of sediment from beds 7 through 11 were screen-washed on site, using sieves with 0.6 mm metal or plastic meshes. The sieved material was left to dry, then it was collected in sample plastic bags and transported to the NMNHS where it underwent further processing. The sediment was soaked in 15% aqueous solution of H_2_O_2_ (hydrogen peroxide) for 15 min, then put in a sieve and rinsed with running water for several minutes, until the removal of the finest fraction. The residual material has been left to dry under normal room conditions and then studied for the presence of microfossils under a MBS-2 stereomicroscope. Plant (charophytes and mesofossils) and invertebrate (decapod fragments and insect eggs) microfossils have also been collected from the samples.

Vertebrate remains were then examined on stereomicroscopes Zeiss Stemi 2000-C with mounted Canon EOS 1300D digital camera and Zeiss Stemi 350 with mounted Zeiss Axiocam 208 Color digital camera. Photographs of the specimens imaged in this study were obtained with software EOS Utility v.3.0 and Labscope v.3.0.1, respectively. The images were processed with the software Helicon Focus 7.0.2, using the function ‘Render’, or with Adobe Photoshop CS5.1, using the functions ‘Automate > Photomerge’ and ‘Auto-Blend Layers’.

### Analysis of the vertebrate fossil content

The distribution and abundance of the vertebrate remains are analysed stratigraphically, taxonomically, and by type of the skeletal element. The input data is presented in Table S1, and subsets of these data are given in Tables S2–4. The values of different parameters are given in percentages of the total, or in some cases a subtract of the total, and are calculated in Microsoft Excel 2019 (see Tables S2–4).

### Bone histology

The osteohistology of ten long bones and bone fragments, pertaining to non-avian dinosaurs (titanosaurs and ornithopods) and a putative pterosaur, is studied to establish their ontogenetic stage (Table 1). The specimens were sampled in 2018 via complete bone sectioning. Regrettably, none of the present authors were involved with this process and the colleagues overseeing it were not trained paleohistologists, which led to sectioning some of the bones slightly oblique to the long axis of the skeletal element, instead of transversely as is the standard in paleohistological studies (Lamm, 2013). However, this mistake does not hamper the interpretation of the obtained results, because the current analysis necessitates only determination of the relative ontogenetic status of sampled fossils and establishing the presence (or absence) of histological or structural features of taphonomic importance if possible. In addition to the aforementioned material, we re-examined the histology of two titanosaur specimens, originally described by Nikolov et al. (2020b).

**Table 1:** Specimens sampled for paleohistological analysis

Specimen	Taxon	Skeletal element	Bed	Primary cortical bone	Trabecular bone	Vascularity	Secondary remodeling	Growth lines	EFS	HOS	RS	Ontogenetic stage	Wedl tunnels	Abrasion	Histology Index
NMNHS FR17	Titanosauria	Femur	8?	WPC (high PFB)	Yes	PO; LAM	Yes	No	No	10–11	4	Subadult	Yes	Yes	4
NMNHS FR24	Titanosauria	Fibula	8	WPC (high PFB)	Yes	PO; LAM, LONG?	Yes	No	No	12–13	9–10?	Subadult	Yes	Yes	3
NMNHS FR16	?Titanosauria	Long bone fragment	ex situ	WPC (high PFB)	Yes	PO; LAM, LONG	Yes, extreme	No	No	14	12	Subadult	Yes	Yes?	3
NMNHS FR55	?Titanosauria	Long bone fragment	ex situ	WPC	Yes	PO; LAM?	Yes, extreme	No	No	13	10	Subadult	Yes	Yes	3–4
U.S., K2 1586	?Titanosauria	Long bone fragment	ex situ	WPC (high PFB)	yes	PO; LAM, LONG	yes, extreme	1 LAG	No	12–13	13	Subadult	?	?	4
NMNHS FR25	Ornithopoda	Tibia	8	WPC (WB; high PFB)	yes (small amount)	PO; LAM	yes, weak	4–6? LAGs	No			Subadult	Yes	Yes	4
NMNHS FR40	Ornithopoda	Tibia	5	LB, WPC	yes	PO; SVC; LONG	yes	2 LAGs	Yes?			Subadult/adult	No	Yes	3–4
NMNHS FR19	Dinosauria indet	(long) bone fragment	ex situ	?	yes	?	yes	?	?			?	?	?	4
NMNHS FR21	Dinosauria indet	(long) bone fragment	8	WPC (high PFB); LB	Yes (small amount)	PO; SVC; LAM	yes	8–10? LAGs	yes?			Adult	No	Yes	2
NMNHS FR23	Dinosauria indet	Metapodial	8	?	Yes	?	Yes, extreme	?	?			Subadult/adult	Yes	Yes	3–4
NMNHS FR54	Dinosauria indet	(long) bone fragment	Ex situ	?	Yes	?	Yes	?	?			?	Yes	Yes	3
NMNHS FR39	?Pterosaria	Indetermined large bone	8	WPC (high PFB); LB	Yes	PO; LAM?	Yes	?	?			subadult/adult	Yes	Yes	4

*EFS* external fundamental system, *HOS* histological ontogenetic stage, *LAG* line of arrested growth, *LAM* laminar bone, *LB* lamellar bone, *LONG* longitudinal vascularization, *PBF* parallel-fibered bone, *RS* remodeling stage, *SVC* simple vascular canal, *WB* woven bone, *WPC* woven-parallel complex

The selected specimens were sectioned at the Petrographic laboratory of the Geological Institute at the Bulgarian Academy of Sciences, Sofia, Bulgaria (GI-BAS), and histological thin sections (or ‘ground sections’) were prepared from the samples following standard paleohistological procedures (Lamm, 2013). The process of thin section preparation is outlined by Nikolov et al. (2020b). Thin sections were examined on a Leica DM2700P petrographic microscope in transmitted plane- and cross-polarized light. Bone histology features of each specimen were imaged with microscope-mounted Leica Flexacam C3 digital camera, using Leica Application Suite X (LAS X) v.5.2.1.27831 software.

For our osteohistological descriptions we employ the terminology of Francillon-Vieillot et al. (1990), along with the term ‘woven-parallel complex’ introduced by Prondvai et al. (2014b). The presence and type of microscopic destruction of bone caused by the process of bioerosion bears taphonomic information for subfossil and fossil bones (Jans, 2022; Trueman & Martill, 2002), and thus we examined the thin sections for signs of MFD. For diagnosis and terminology regarding the types of MFD, we follow Hackett (1981). A qualitative Histological Index, which summarizes the degree of diagenetic change the bone has undergone, is established for all sampled fossils based on the work of Hedges et al. (1995). For the titanosaur material the Histological Ontogenetic Stage (HOS) and Remodeling Stage (RS) of each specimen have been determined following Klein and Sander (2008) (see also Stein et al., 2010) and Mitchell et al. (2017), respectively. Both HOS and RS are used to establish the relative ontogenetic status of sauropod remains, long bones in particular, on the basis of the features of the cortical bone tissues (e.g., type of bone matrix, vascular architecture and density, degree of secondary remodeling and number of secondary osteon generations, etc.) (Klein & Sander, 2008; Mitchell et al., 2017). While the 14 established HOS give general information about the ontogenetic status of fossil bones, with higher HOS generally indicating more advanced ontogenetic stages (Klein & Sander, 2008; but see Stein et al., 2010), the RS is developed to extend the histologic ontogenetic stages into senescence for specimens with strongly or completely remodeled cortical tissues (Mitchell et al., 2017). There are a total of fifteen remodeling stages (RS 1–15) recognized in the original sample of sauropod bones studied by Mitchell et al. (2017), with higher stages reflective of a more advanced ontogenetic status of the skeletal element (for details see Mitchell et al., 2017).

### Potential biases

As with many paleontological studies, there are some potential biases, both objective and subjective, which might skew the analysed data and affect the subsequent interpretation, and thus warrant mention. First and foremost, the topography of the fossil locality—a deep densely forested gully in a mountainous region—and the studied outcrop—a vertical wall—prevents extensive surface excavation and any usage of grid or drawing of a site map. In result, it is difficult to ascertain the spatial relationships between the fossils found in each bed. This leads to the loss of some taphonomic information upon excavation. Additionally, despite the attempted systematic approach to the work conducted at the site, the discovery and collection of vertebrate macrofossils remain largely opportunistic endeavors due to the aforementioned specifics of the site. The limited access to the horizontal plane of the fossiliferous beds certainly leads to underestimation of the actual number of fossils contained in those beds. The proportion of microvertebrate material is probably an underrepresentation of the actual abundance of microvertebrates at the site, due to the uneven stratigraphic sampling and the relatively small amount of processed sediment per bed caused by limited resources and insufficient working power. However, the taxonomic content and its characteristics are fairly consistent through the studied section, which gives us the confidence that, despite biases at play, the observed trends reflect the actual state of the fossil record at the Vrabchov Dol locality.

## Results

### Palynological content and palynostratigraphy

#### Palynological content

**Fig. 5:**
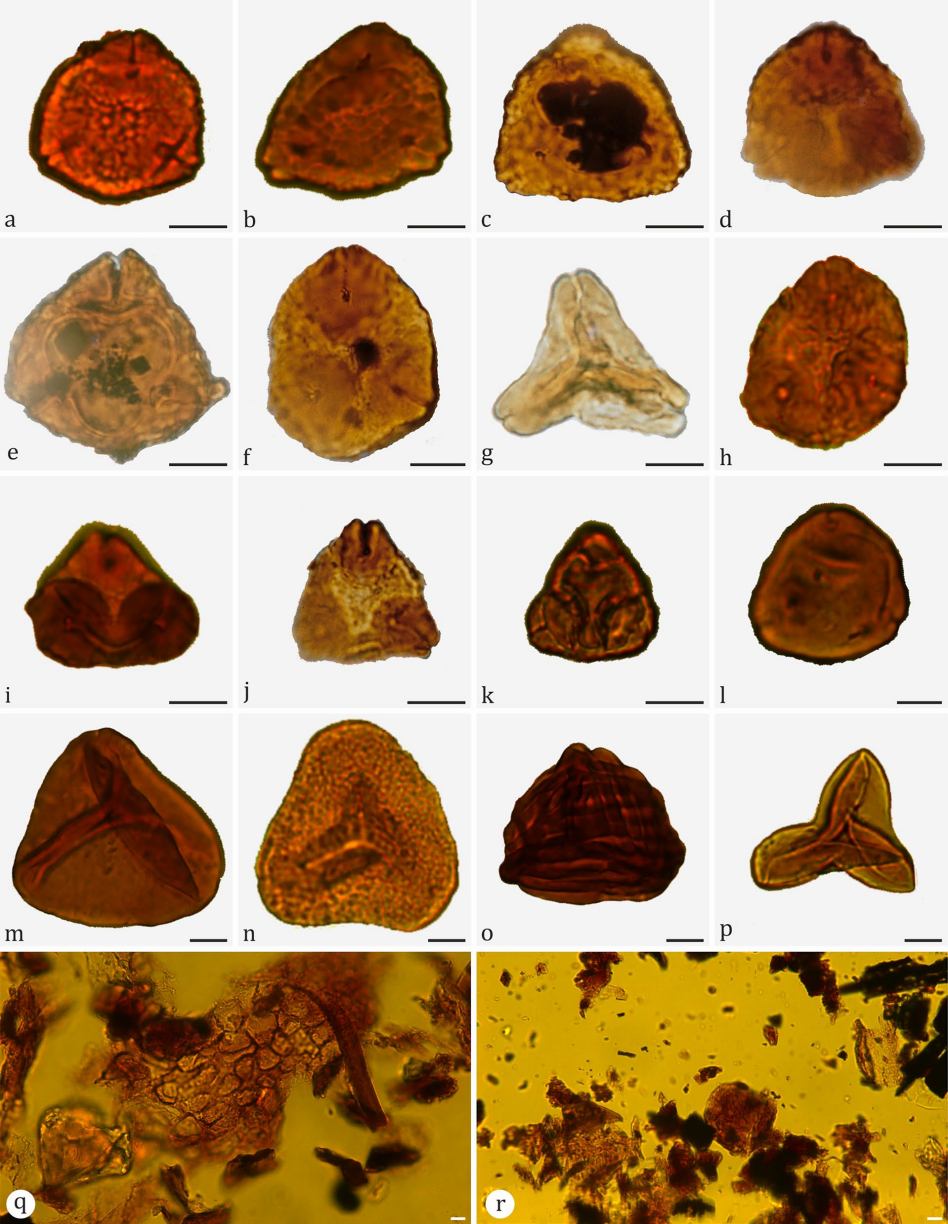
Pollen and spores from Vrabchov Dol. **a** *Krutzschipollis magnoporus* Góczán in Góczán et al., 1967, sample T/3. **b, c** *Krutzschipollis crassus* (Góczán, 1964) Góczán in Góczán et al., 1967, sample T/3. **d** *Oculopollis zaklinskaiae* Góczán, 1964, sample T/2. **e** *Krutzschipollis spatiosus* Góczán in Góczán et al., 1967, sample T/3. **f** *Oculopollis orbicularis* Góczán in Góczán et al., 1967, sample T/3. **g** *Extrapollis bohemicus* Krutzsch and Pacltová, 1967 in Góczán et al., 1967, sample Vrb-6. **h** *Oculopollis zaklinskaiae* Góczán, 1964, sample T/2. **i, j** *Oculopollis principalis* Weyland & Krieger, 1953, sample T/2. **k** *Trudopollis pertrudens* Pflug, 1953, sample Vrb-8. **l** *Interporopollenites* sp., sample Vrb-3. **m** *Deltoidospora ordinata* Brelie, 1964, sample T/2. **n** *Vadaszisporites urcuticus* (Deák, 1964) Góczán in Góczán et al., 1967, sample T/3. **o** *Cicatricosisporites hughesii* Dettmann, 1963, sample T/1. **p** *Cyathidites minor* Cookson, 1947, sample Vrb-3. **q, r** Palynofacies type dominated by large translucent phytoclasts and plant cuticles, samples Vrb-3 and T/3. Scale bar: 10 μm. All photomicrographs were taken using conventional light microscopy

In the section of Vrabchov Dol, the sampled vertebrate-bearing succession yielded well preserved palynological assemblages of moderate to low species composition of terrestrial and rare marine palynomorphs (Fig. 5). They include pollen grains, plant spores, rare dinoflagellate cysts and micro-foraminiferal test linings at some levels (Fig. 4). Terrestrial palynomorphs dominate the associations. The most abundant angiosperm pollen grains belong to the morphologically characteristic Normapolles group with the encountered species *Oculopollis zaklinskaiae* Góczán, 1964, *Oculopollis orbicularis* Góczán, 1964, *Oculopollis principalis* Weyland & Krieger, 1953, *Krutzschipollis crassus* (Góczán, 1964) Góczán, 1967, *Krutzschipollis spatiosus* Góczán, 1967, *Krutzschipollis magnoporus* Góczán, 1967 together with *Extrapollis bohemicus* Krutzsch & Pacltová, 1967 (in Góczán et al., 1967)*, Interporopollenites* spp*., Vacuopollis* sp. and *Trudopollis* sp. Gymnosperm pollen is rare in the assemblages and represented only by *Araucariacites australis* Cookson, 1947. The spores are dominated by *Deltoidospora ordinata* Brelie, 1964, *Deltoidospora* sp.*, Cyathidites minor* Cookson, 1947, *Vadaszisporites urkuticus* (Deák, 1964) Deák & Combaz, 1967, *Vadaszisporites sacali* Deák & Combaz, 1967, *Cicatricosisporites hughesii* Dettmann, 1963*, Verrucosisporites acrostichoides* Góczán, 1964, and *Todisporites major* Deák & Combaz, 1967 (Figs. 4, 5).

The highest proportions of the pollen and spore content are encountered in samples from beds 2, 3, 4, 5, 6 and 8. Dinoflagellate cysts are found as single occurrences only in samples from beds 3 and 7 and are represented by *Deflandrea* spp., *Circulodinium* sp. and *Senoniasphaera* sp. These samples also contain rare micro-foraminiferal test linings. The average proportion of dinocysts and marine elements in these levels is around 8% of the total palynomorphs in the samples.

Considering the floral characteristics, the recovered species association is dominated by 10 species of angiosperm pollen (core Fagales). Gymnosperms are represented by one conifer species (such as Araucariaceae). Fern spores are dominated by Dipteridaceae and Dicksoniaceae (*Deltoidospora*), Cyatheaceae (*Cyathidites*) and Schizaeaceae (*Cicatricosisporites*). Palynomorphs interpreted as humidity indicators are represented by all encountered spore species belonging to the genera *Deltoidospora, Cyathidites*, *Cicatricosisporites* and *Vadaszisporites* as well as to some extent to the encountered angiosperm Normapolles species. Abundance of these humidity indicators is recorded throughout the studied sedimentary succession.

#### Palynostratigraphy and age assessment

From a palynostratigraphic point of view, age diagnostic within the palynomorphs is the angiosperm pollen of the Normapolles group. This pollen group dominates and is comparatively well diversified throughout the entire section. Most profuse genera are *Oculopollis* and *Krutzschipollis.* The pollen assemblages are generally similar in all samples and comprise key taxa of high correlation value such as *Oculopollis zaklinskaiae*, *Oculopollis orbicularis*, *Krutzschipollis crassus*, *Krutzschipollis spatiosus* and *Krutzschipollis magnoporus* (Fig. 4). The assemblages give important biostratigraphic information directly from the vertebrate-bearing intervals. The *Krutzschipollis crassus*—*Krutzschipollis spatiosus* Association is recognized in the section. It is correlated to the Normapolles zonations for the Santonian—Campanian interval from the Transdanubian Range in Hungary, Southern France and Spain, North Bulgaria and the Gosau Group in Austria (Bodor & Baranyi, 2012; Góczán, 1964; Góczán & Siegl-Farkas, 1990; Góczán et al., 1967; Médus, 1972; Pavlishina, 1999; Pavlishina et al., 2004; Siegl-Farkas & Wagreich, 1996). Médus (1972) first pointed out the predominance of *Oculopollis* representatives in the Santonian palynofloras from Southern France and Spain, emphasizing that *Oculopollis orbicularis* makes its first occurrence within the middle part of the Santonian. Pavlishina (1999) recognized two successive pollen assemblages and denominated them as *Oculopollis zaklinskaiae—Krutzschipollis crassus* Association for the lower and middle part of the Santonian and *Krutzschipollis crassus—Krutzschipollis spatiosus* Association for the upper Santonian–lower Campanian interval in North Bulgaria. She pointed out the first occurrence of *Krutzschipollis spatiosus* in the upper Santonian. Krutzsch (in Góczán et al., 1967) noticed that the representatives of *Krutzschipollis* diversify during the Campanian in Central Europe, while *Krutzschipollis crassus* and *Krutzschipollis spatiosus* dominate the early Campanian palynofloras in Central Europe and the Transdanubian Range of Hungary (Bodor & Baranyi, 2012; Góczán, 1964; Góczán et al., 1967; Siegl-Farkas & Wagreich, 1996).

Based on the established ranges of species, especially in dinocyst and ammonite calibrated sections in North Bulgaria and the Gosau Group in Austria, the concurrent presence of the pollen species *Oculopollis zaklinskaiae*, *Oculopollis orbicularis*, *Krutzschipollis crassus* and *Krutzschipollis spatiosus* is regarded as characteristic for the latest Santonian and early Campanian assemblages. The recognized *Krutzschipollis crassus*—*Krutzschipollis spatiosus* Association through the studied section indicates a latest Santonian–early Campanian age for the vertebrate-bearing succession at Vrabchov Dol.

### Palynofacies analysis

**Fig. 6:**
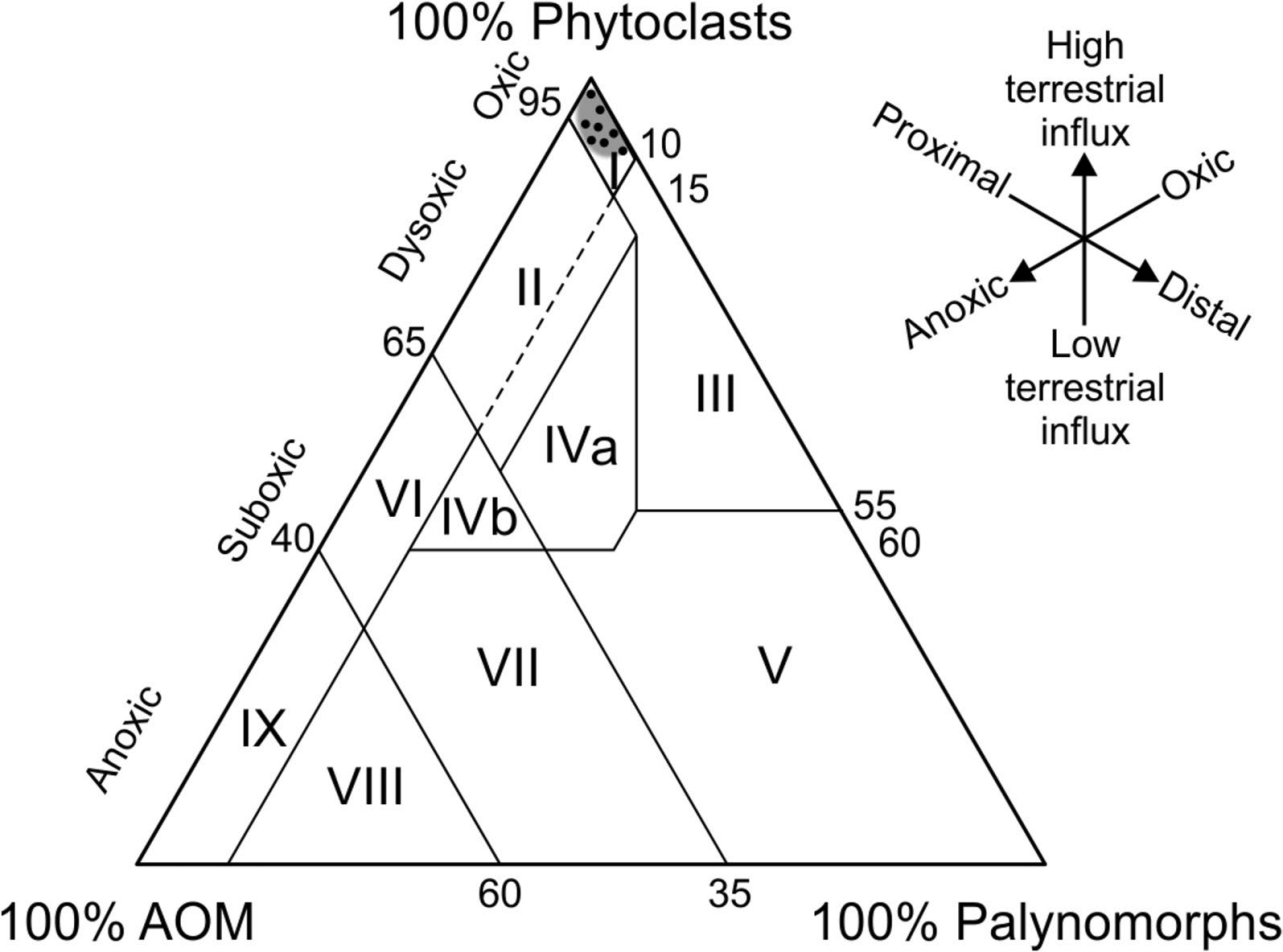
Palynofacies ternary diagram (after Tyson, 1995) and position of the palynological samples

All samples from the Vrabchov Dol succession appeared to be rich in organic matter (OM). One type of palynofacies covers most intervals of the studied succession and represents approximately more than two-thirds of the total number of samples. This palynofacies is characterized by high abundances of total phytoclasts (80–90%), compared to moderate to low AOM and palynomorph contents of the total OM composition (Fig. 6). Phytoclasts are represented mainly by translucent organic particles, including tissues, wood remains and plant cuticles (Fig. 5). Opaque phytoclasts are subordinate in the assemblages. The shape of all phytoclasts is mostly equidimensional suggesting short transportation. The values of the OP/TR ratio are low and show predominance of the translucent phytoclasts in all slides. The C/M ratio (continental to marine elements) is very high, since continental elements, together with pollen and spores, collectively make up a high proportion in the samples, up to 90%. AOM is present in all samples up to 5% in the slides. Spore tetrades are found in samples from beds 2, 3, 5 and 9 once more confirming the short transportation in the depositional environment.

Only two samples from the succession, those from beds 3 and 7, contain rare dinoflagellate cysts and micro-foraminiferal test linings (8%), indicating minor marine influx at these levels within the studied sedimentary succession. The representatives of the encountered dinocyst genus *Circulodinium* are presumed to be brackish, littoral or inner neritic or commonly regarded as being indicative of reduced salinity. This could be also supported by the presence of foraminiferal linings, which tend to be more abundant in high-productivity areas, including nearshore settings, related to river discharge (Niechwedowicz et al., 2021).

All samples of the recognized palynofacies type plot in the palynofacies field I of the APP ternary diagram (Fig. 6), which is representative of deposition in a highly proximal shelf, or even oxidated lagoonal paleoenvironments, with short transportation of the continental elements, such as spores, pollen and cuticles.

### Non-vertebrate fossils

#### Plant fossils

**Fig. 7:**
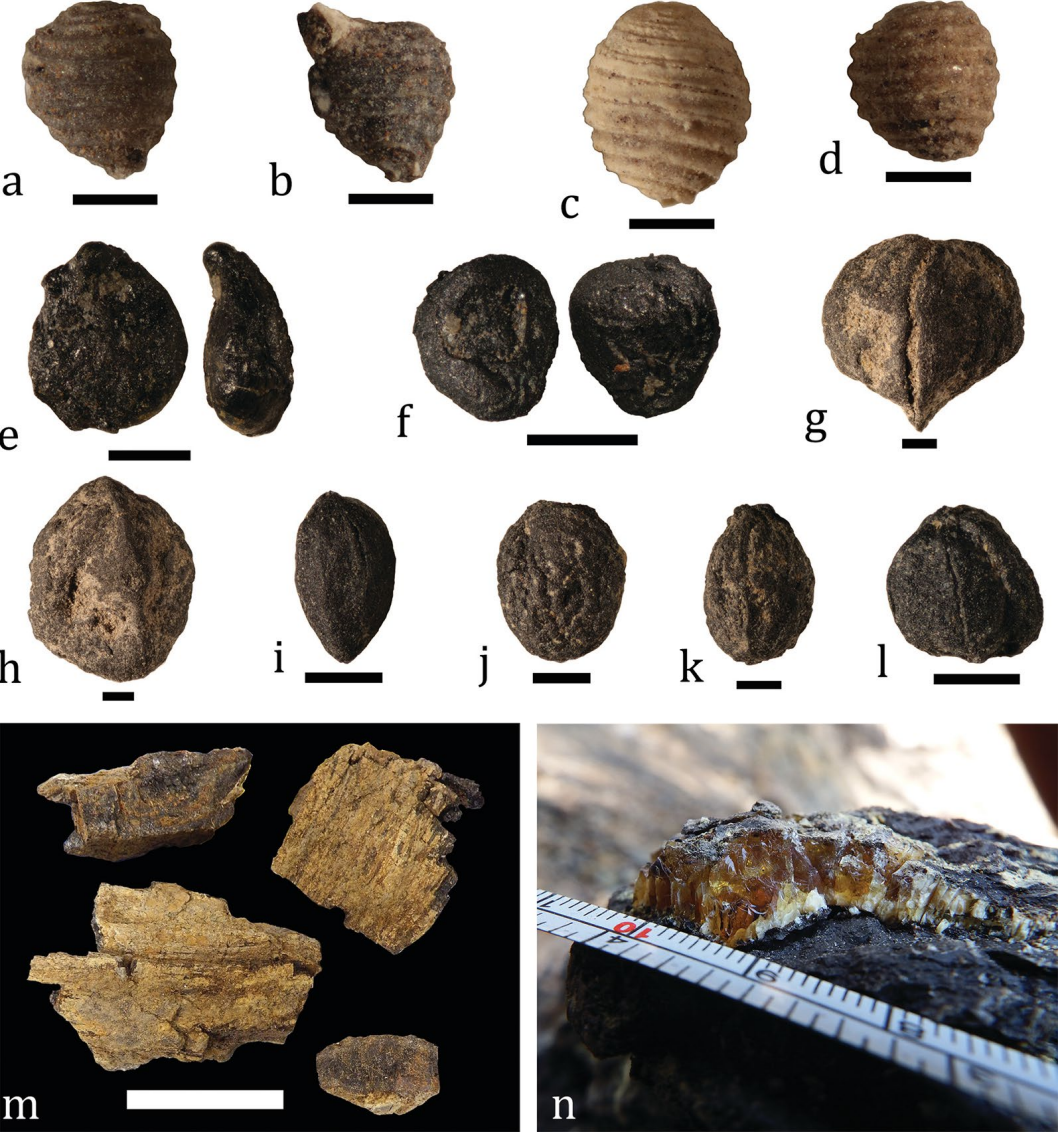
Algae and plant fossils from Vrabchov Dol. **a–d** Charophyte gyrogonites from bed 7 (**a**, **b**) and 8 (**c**, **d**). **e–l** Plant mesofossils. **m** Carbonized wood from bed 8. **n** Fossilized resin from bed 4. Scale bar: **a**–**d**: 500 μm; **e**–**l**: 1 mm; **m**: 50 mm

Various types of plant remains and fossil resin are found at the site. Rare, moderately to poorly preserved charophyte gyrogonites are collected from sediment samples from beds 7 and 8 (Fig. 7a–d). Charophyte algae inhabit diverse non-marine environments and their fossils are common in a variety of continental deposits (Schneider et al., 2015). The small number of specimens (n = 4) and their relatively poor state of preservation imply transportation and allochthonous nature of the material. Fossils of land plants are understandably para-autochthonous or allochthonous components of the fossil assemblage. Outside spores and pollen, higher plants are represented by numerous and morphologically diverse mesofossils, i.e. seeds and fruits (Fig. 7e–l). Mesofossils are present in rock samples from beds 5, 7, and 8. While coal lenses and indeterminable carbonized plant matter are common in certain beds of the section, recognizable pieces of carbonized wood appear mostly in the upper half of bed 8, especially around the boundary with bed 9, and much more rarely in bed 11. Although most of the collected specimens are under 10 cm in length, this size is the result of breakage due to the high fragility of the material. The wood is encrusted by Fe-hydroxide mineralization, which imparts rusty to reddish surface color (Fig. 7m). Fossilized resin is found in all vertebrate-bearing beds, except for bed 11. Its size and shape vary greatly from mm-wide droplets to crusts or lenses up to several centimeters in length (Fig. 7n). Most of the collected material consists of pieces one to few millimeters in size. The droplets are typically opaque but larger pieces are semi- to completely transparent. The color of the resin and related products is highly variable – from milky white to yellowish, orange and even red. No bioinclusions have been found to date.

#### Invertebrate fauna

**Fig. 8:**
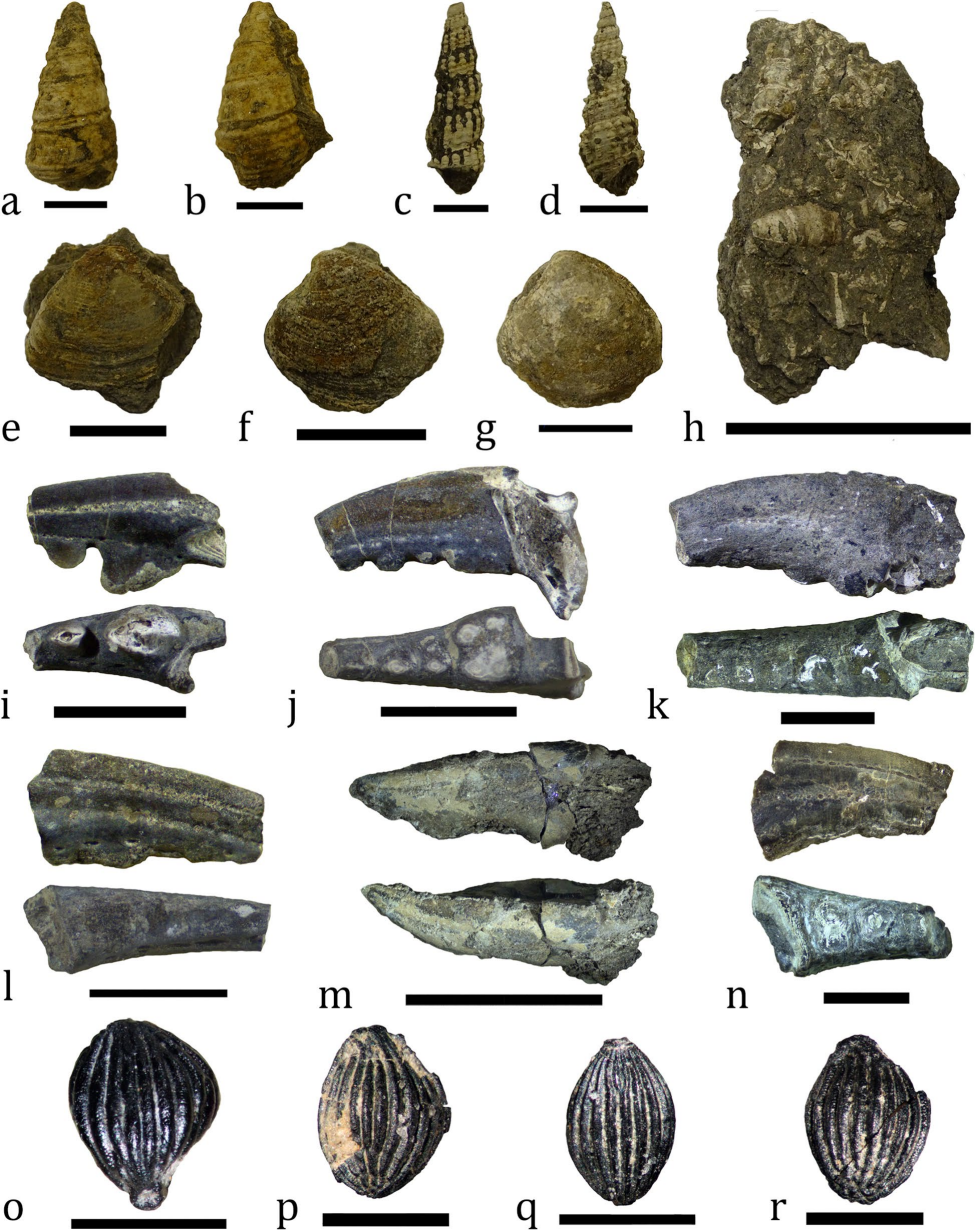
Invertebrate fossils from Vrabchov Dol. **a-d** Gastropods. **e–g** Bivalves. **h** Fossiliferous rock sample from bed 9. **i–n** Fragments of dactylae and fixed fingers of decapods. **o–r** Specimens attributable to the genus *Knoblochia* (NMNHS F31811, F31799, F31801, F31806). Scale bar: **a**, **b**, **e**, **f**, **m**: 20 mm; **c**, **d**, **k**, **n:** 5 mm; **g**, **o**–**r**: 1 mm; **h**: 40 mm; **i**, **l**: 2 mm; **j**: 3 mm

Most of the beds of the studied section contain abundant invertebrate fauna, dominated by mollusks. They are present as shell detritus and fragmentary or complete, but predominantly poorly preserved, shells. Partial Fe-hydroxide coating or clusters of small-sized gypsum crystals are frequently observed on fossil specimens. Gastropods are most numerous and are particularly abundant in the upper third of the section. Their shells range from a few millimeters to over 5 cm in height (Fig. 8a–d). Bivalves are less common and more poorly preserved in comparison to gastropods (Fig. 8e–g). Beds 8 and 9 yielded oyster remains. Decapods are the third group of invertebrates found at Vrabchov Dol. Although six out of the eleven beds at the site yield decapod fossils, these are extremely fragmentary and consist only of pieces of chelipeds’ dactylae and fixed fingers, more rarely leg fragments (Fig. 8i–n). Fossils referable to the genus *Knoblochia* are collected from beds 6, 7, and 8 (Nikolov et al., 2024) (Fig. 8o–r). Traditionally considered to be a plant mesofossil, this taxon has been recently reinterpreted as an insect egg, possibly pertaining to a representative of either clade Phasmatodea or Lepidoptera (Heřmanová et al., 2013). Currently, fossils of *Knoblochia* are restricted to the Upper Cretaceous of Europe, where they are known from both marine and continental deposits (Bodor et al., 2014; Heřmanová et al., 2013; Marmi et al., 2016). Within the context of the studied locality, the insect eggs are an allochthonous component of the assemblage. Excluding the insect material, the Vrabchov Dol invertebrate fauna is generally suggestive of a brackish environment, yet it warrants a detailed taxonomic study in order to better understand its paleoecology.

### Vertebrate assemblage data

#### General characteristics

**Fig. 9:**
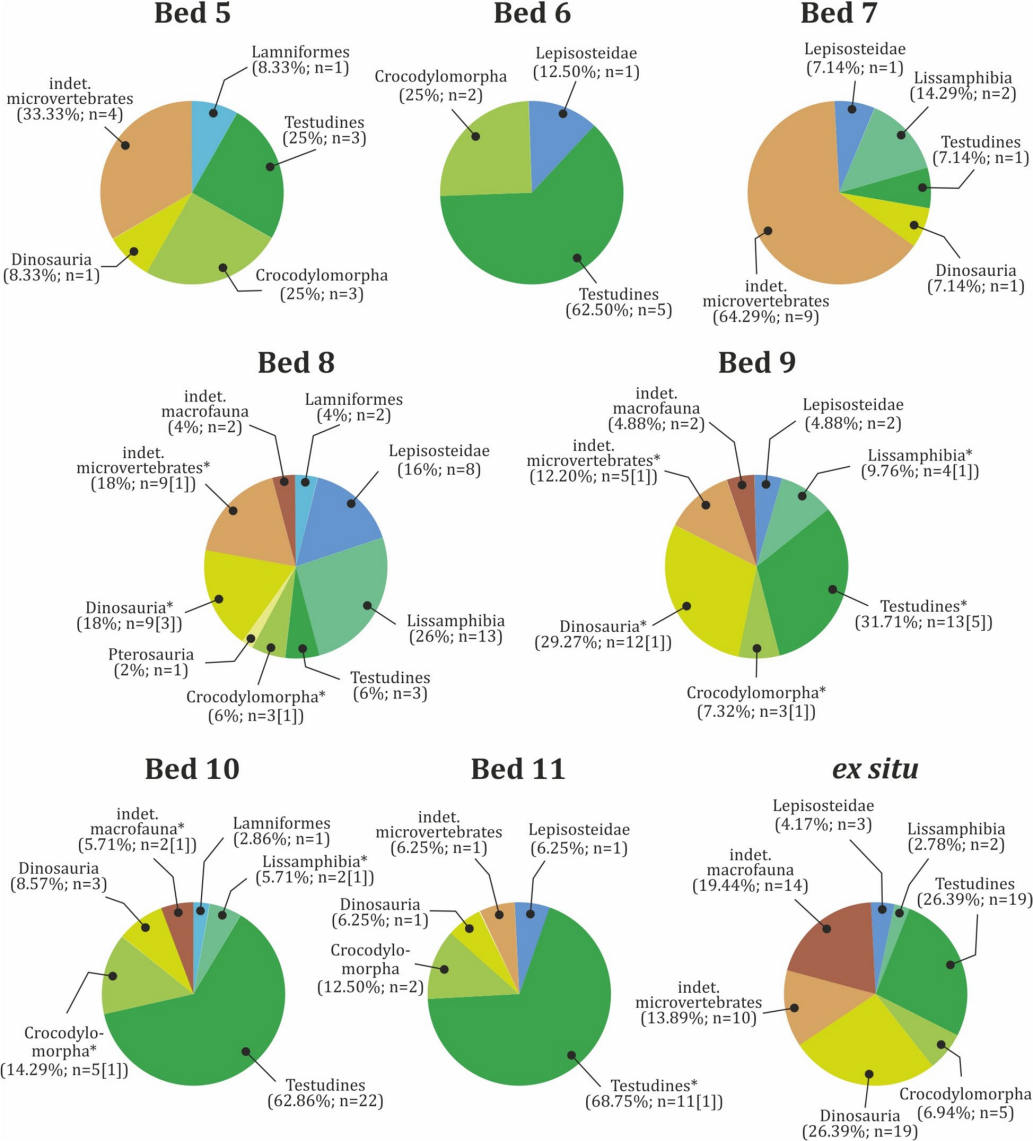
Taxonomic content and abundance of vertebrate-bearing strata. Input data are present in Table S2. Taxa marked with asterisks ‘*’ include specimens which are tentatively referred to the particular stratum (bed). Numbers given in angle brackets ‘[]’ indicate the number of specimens with tentative stratigraphic position in addition to the specimens with robust stratigraphic referral

The vertebrate assemblage at Vrabchov Dol includes the fossil remains of a minimum of seven clades of vertebrates, whose mode of life ranges from strictly terrestrial to fully aquatic (Fig. 3). The fossils range in size from microvertebrate bone fragments under 1 mm in length to non-avian dinosaur limb elements over 40 cm in length. Skeletal remains include teeth, vertebrae, long bones, a single osteoderm, partial turtle carapaces, plastron and shell fragments, and indeterminate bone fragments. Notably, unequivocal cranial remains (aside from teeth) are yet to be found at the site. Characteristic of the assemblage is the disarticulated and isolated nature of the skeletal elements. Only two specimens, NMNHS FR43a and NMNHS FR43b, interpreted as an ornithopod radius and ulna, respectively, are assumed to be associated on the basis of their corresponding size and immediate proximity to each other. Although we have not been able to collect exact spatial data, field observations made by our team suggest that the excavated in situ long bones are oriented generally in W-E to NNW-SSE direction (Nikolov, pers. obs.). Fossils do not appear to be sorted by size or by type of skeletal elements, with various bones of microvertebrates and vertebrate macrofauna occurring in the same bed. The state of preservation ranges from good to poor, but well-preserved complete skeletal elements are very rare. Fossils are preserved three dimensionally, although some of the specimens, particularly large bones, exhibit variable degree of post-burial deformation. Virtually all sufficiently complete fossils and large bone fragments suffer from severe diagenetic fracturing, seemingly the result of tectonic activity. All vertebrate-bearing beds yield remains of macrofauna. The taxonomic content is generally consistent throughout the studied rock section, with remains of turtles, crocodylomorphs, and non-avian dinosaurs typically co-occurring in the same stratum, albeit in different proportions (Fig. 9).

#### Stratigraphic distribution

**Fig. 10:**
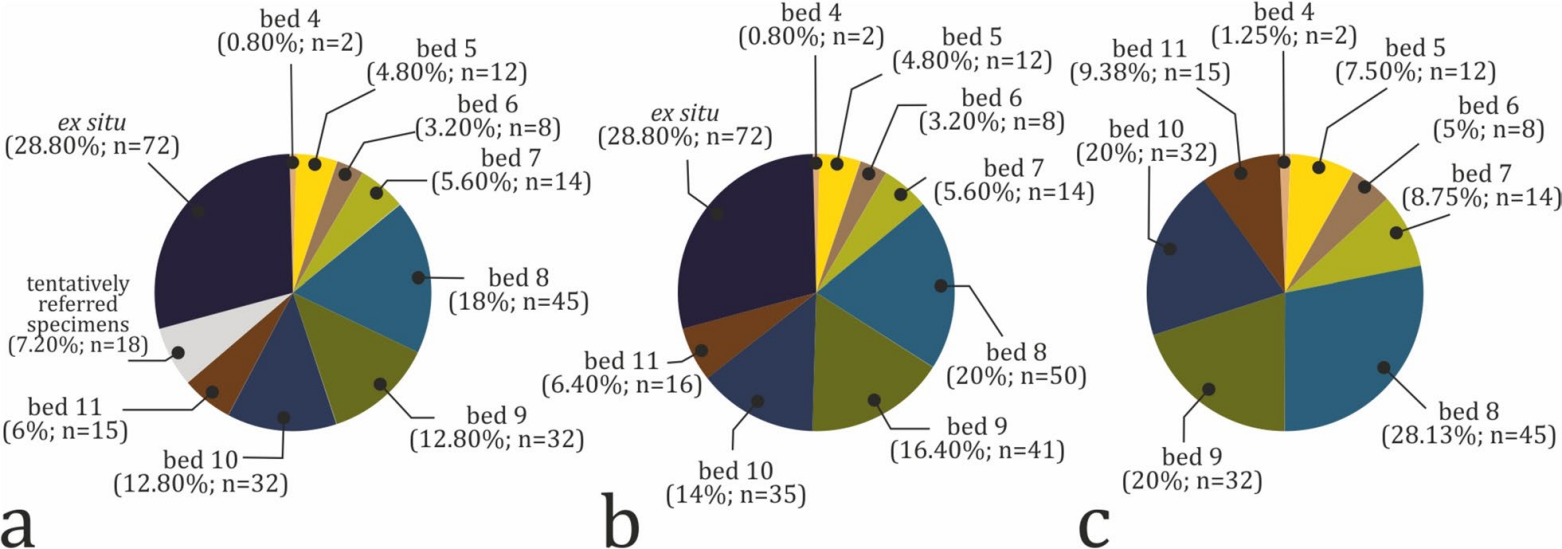
Stratigraphic distribution of fossil vertebrate material. **a** Total distribution. **b** Distribution of fossil material with specimens with inferred stratigraphic position tentatively referred to particular beds. **c** Distribution of the fossil material including only specimens with well constrained stratigraphic position. Input data are present in Table S2

Vertebrate fossils are found in eight beds, but their distribution within the studied section is uneven (Fig. 10a). Of all 250 specimens, only 64% (n = 160) can be assigned stratigraphically to a particular stratum, while 28.80% (n = 72) of the material was found ex situ. The remaining 18 specimens (7.20%) were found ex situ or their stratigraphic position is poorly documented, but thanks to some sediment matrix still present on their surface their host rock can be tentatively deduced. The four uppermost strata of the section have produced 142 specimens (including the stratigraphically tentatively referred specimens), or 56.80% of the fossil material found at the locality (Fig. 10b). Beds 4 and 6 yielded the fewest fossils, two (0.80%) and eight (3.20%) specimens, respectively. Among the fossil-bearing strata, bed 8 is richest in fossil content, producing 28.13% (n = 45) of all stratigraphically constrained specimens (Fig. 10c), followed by beds 9 and 10, with 32 fossils each. Again, bed 8 appears to contain the most taxonomically diverse vertebrate assemblage. Its macrofauna is dominated by dinosaurs (n = 9), while the most common microvertebrates are frogs (n = 13), followed by gars (n = 8) (Fig. 9). Turtle remains are the most numerous vertebrate fossils in beds 9 through 11.

#### Skeletal elements

**Fig. 11:**
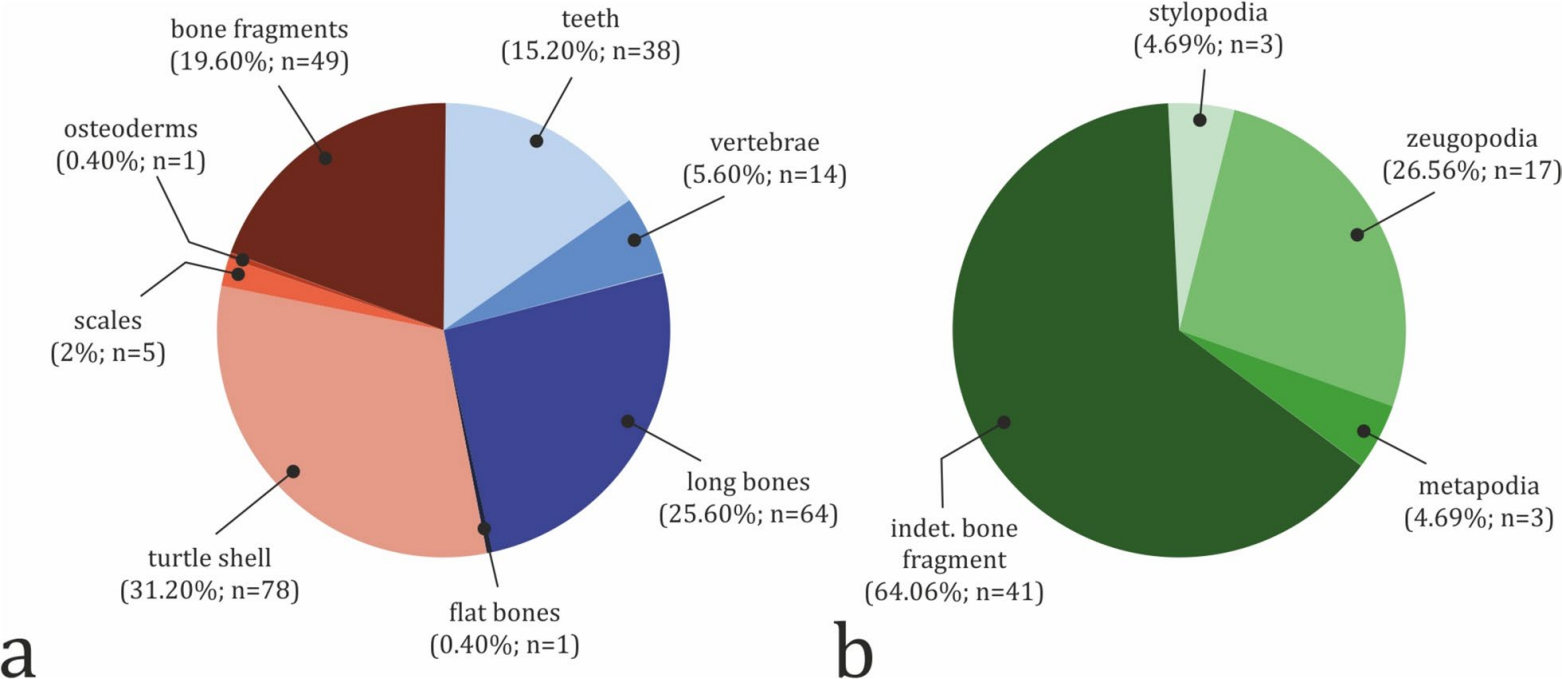
Fossil abundance by type of skeletal element. **a** Total abundance by type of skeletal element. **b** Abundance of different types of long bones. Input data are present in Table S3

**Fig. 12:**
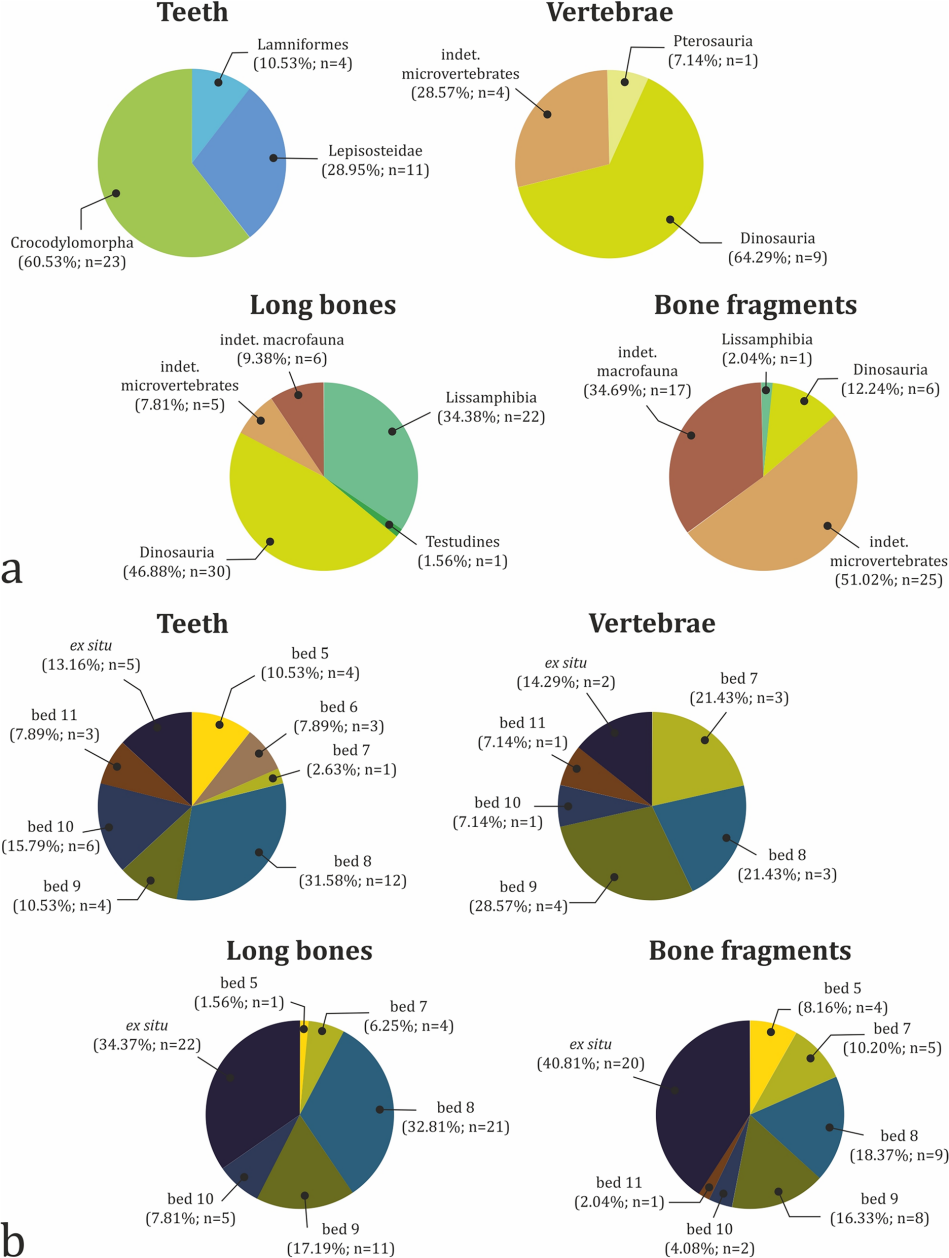
Taxonomic and stratigraphic distribution of the major types of skeletal elements found at Vrabchov Dol. **a** Taxonomic distribution. **b** Stratigraphic distribution. Input data are present in Table S3

Turtle shell fragments, including partial plastron and large carapace pieces, are the most common type of vertebrate fossil found at the site (n = 78; 31.20%) (Fig. 11a). Second most common are appendicular skeletal elements, which constitute 25.60% of the sample (n = 64), followed by teeth, which account for 15.20% of collected fossils (n = 38). About 60% of all teeth belong to crocodylomorphs, while the rest are from chondrichthyans (10.53%) and osteichthyans (28.95%) (Fig. 12a). Teeth and long bones are most numerous in bed 8 with 12 and 21 specimens, respectively (Fig. 12b). The majority of the long bone material consists of currently indeterminate or poorly diagnostic bone fragments (64.06%; n = 41). Of the diagnostic specimens, there are three stylopodial (4.69%), 17 zeugopodial (26.56%), and three metapodial elements (4.69%) (Fig. 11b). The majority of the long bone material is dinosaurian (46.88%; n = 30), with the next best represented clade being Lissamphibia (34.38%; n = 22) (Fig. 12a). Vertebrae are notably rarer, representing only 5.60% (n = 14) of the discovered material. Of these, nine vertebrae belong to dinosaurs (64.29%). Other types of skeletal elements found at the site are flat bones (a single specimen), osteoderms (a single ossicle), and fish scales (n = 5), which together account for just 2.80% of all fossils. Interestingly, no unequivocal cranial remains have been discovered so far, although we cannot rule out the possibility that some of the poorly preserved indeterminate material, especially that of microvertebrates, might represent skull bone fragments.

#### Taxonomic content

**Fig. 13:**
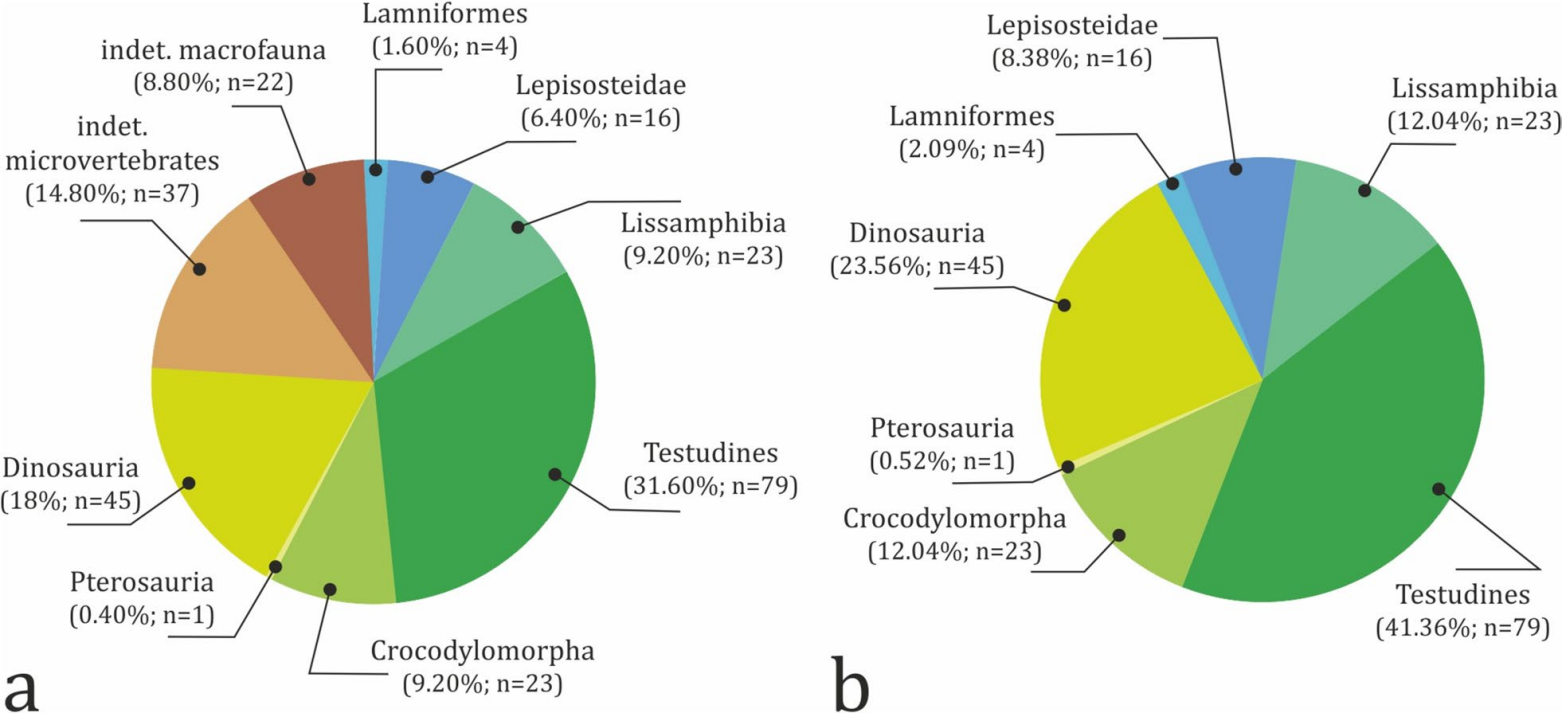
Abundance of vertebrate taxa in the Vrabchov Dol vertebrate assemblage. **a** Total abundance. **b** Abundance of vertebrate taxa, excluding fossil specimens of indeterminate micro- and macrofauna. Input data are present in Table S4

The fossil remains of representatives of at minimum seven, but possibly eight, major vertebrate clades are found at Vrabchov Dol: Chondrichthyes, Osteichthyes, Lissamphibia, Testudines, Crocodylomorpha, ?Pterosauria, Titanosauria, and Ornithopoda. Currently, only 76.40% of all specimens (n = 191) are reliably ascribed to one of these taxa (Fig. 13a). Similar to other European Upper Cretaceous vertebrate localities (Botfalvai et al., 2015; Buffetaut et al., 1999; Pereda-Suberbiola et al., 2000), turtles appear to be most abundant, comprising 31.60% (n = 79) of all collected specimens. They also exhibit the widest and most consistent stratigraphic range within the sedimentary section (Fig. 3). The next best represented clades are non-avian dinosaurs (18%), followed by lissamphibians and crocodylomorphs (9.2% each). With the exception of chondrichthyans, which are marine inhabitants, the vertebrate fauna at Vrabchov Dol consists of terrestrial and freshwater to brackish, semi-aquatic and aquatic forms. The fossil record of each recognized vertebrate taxon is further analysed in the following paragraphs.

##### Chondrichthyes

**Fig. 14:**
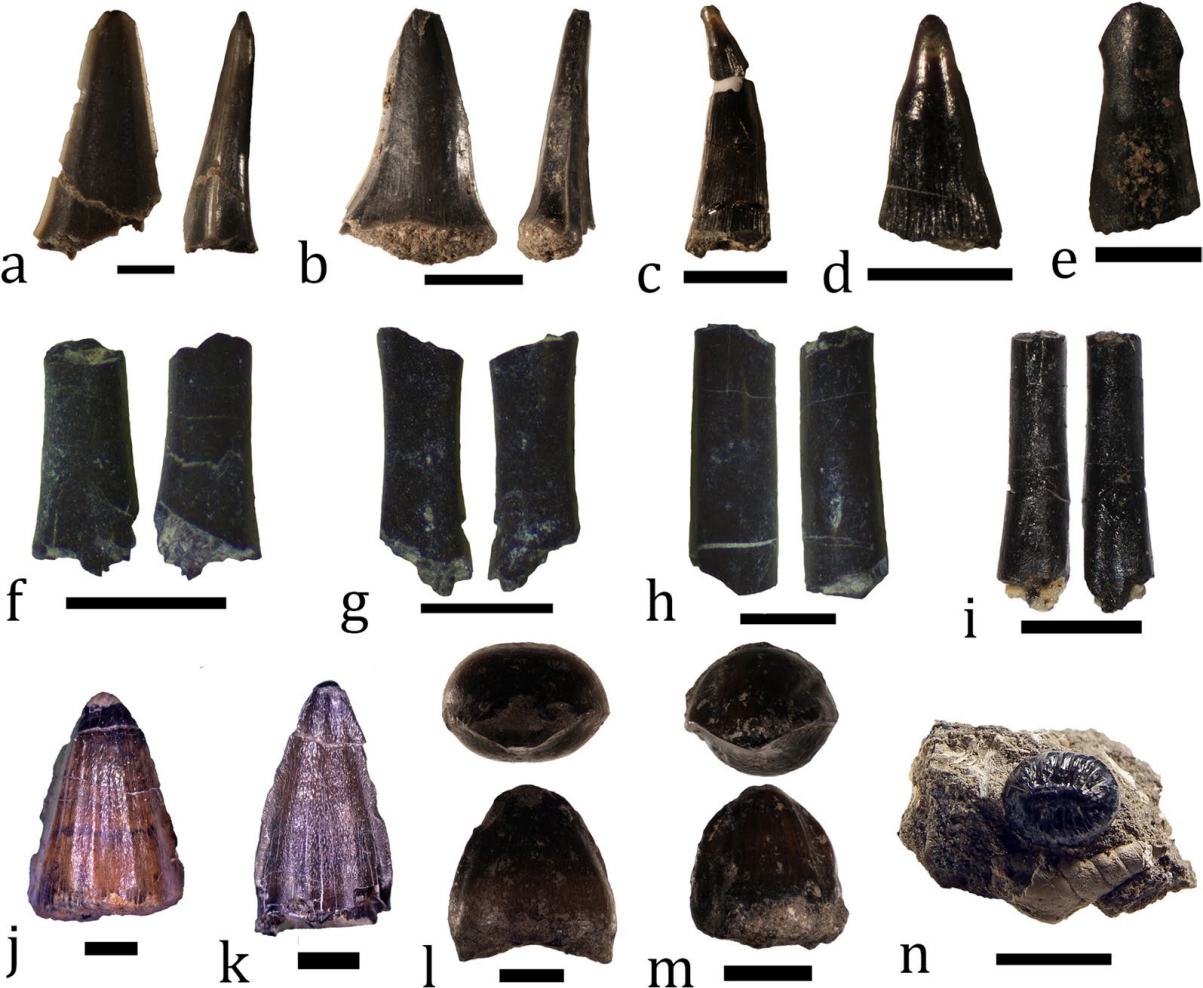
Microvertebrate material. **a** Lamniform tooth (NMNHS FR80) from bed 5. **b** Lamniform tooth (NMNHS FR81) from bed 8. **c–e** lepisosteid teeth (specimens NMNHS FR72, FR66, FR74). **f–i** amphibian ?radioulnae (**f–g**) and tibiofibulae (**h**, **i**) (three uncatalogued specimens and NMNHS FR53). **j, k** Allodaposuchid tooth crowns (specimens NMNHS FR27, FR28). **l–n** uncatalogued putative hylaeochampsid tooth crowns. Photos of specimens NMNHS FR27, FR28 and FR53 are courtesy of Boyan Zlatkov and Latinka Hristova and are used here with their permission. Scale bar: **a**, **c**–**i:** 1 mm; **b**: 3 mm; **j**–**m**: 2 mm; **n**: 4.5 mm

**Fig. 15:**
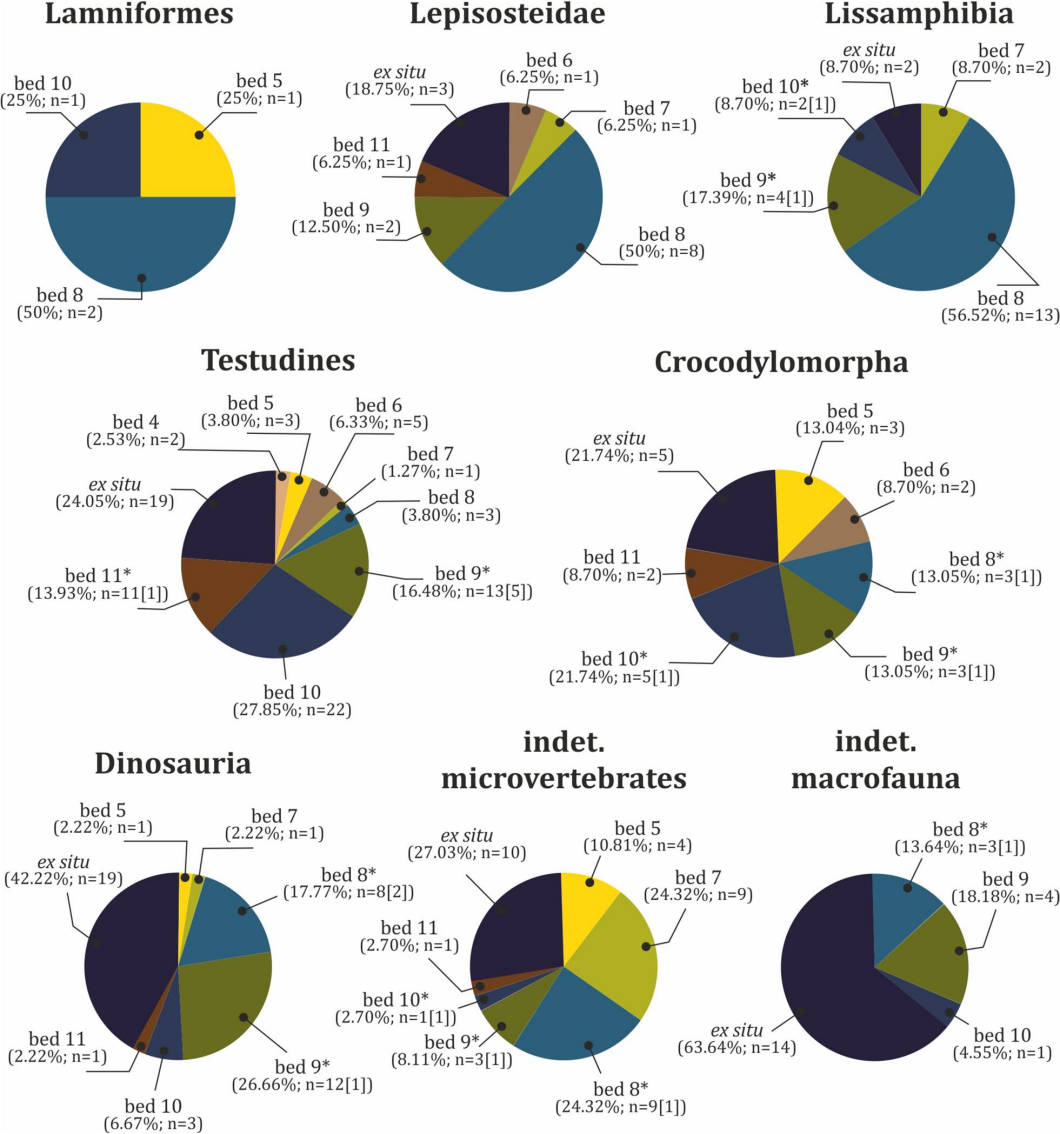
Stratigraphic distributions of the vertebrate groups present in the Vrabchov Dol assemblage, including material from indeterminate microvertebrates and macrofauna as separate groups. Stratum (bed) marked with asterisks ‘*’ includes specimens which are tentatively referred to it. Numbers given in angle brackets ‘[]’ indicate the number of specimens with tentative stratigraphic position in addition to the specimens with robust stratigraphic referral. Input data are present in Table S4

Cartilaginous fish are the rarest component of the fossil assemblage. They are represented by four tooth crowns attributable to the clade Lamniformes (P. Andreev, pers. comm.), which is just 1.60% of all specimens (Fig. 13a). The fossils are rather poorly preserved, lacking the tooth root and thus hardly diagnostic at lower taxonomic level. Two of the crowns are characterized by a generally straight lingual surface, while in the other two the surface is weakly sinusoidal apicobasally (Fig. 14a, b). The lamniform sharks are the only certain marine component of the studied vertebrate fauna. The presence of their remains in beds 5, 8, and 10 suggest a more pronounced marine influence during the deposition of these strata (Fig. 15).

##### Osteichthyes

Bony-fishes are represented by indeterminate lepisosteids (gars) (Fig. 14c–e). The gar material consists of 11 partially preserved teeth and five scales; these account for 6.4% of all fossils (Fig. 13a). These remains have been collected from beds 6 through 9. A single specimen is collected from bed 11 and three fossils are found ex situ. Half of the specimens (n = 8) come from bed 8 (Fig. 15). Most of this material has been described by Nikolov et al. (2025). These authors interpreted the gar fossils as an allochthonous component of the assemblage, being transported from more inland, freshwater environments.

##### Lissamphibia

Currently, the presence of lissamphibians in the assemblage is recognized on the basis of 23 fragmentary long bones, radioulnae and tibiofibulae in particular (Fig. 14f-i). These make 9.20% of all collected fossil material (Fig. 13a) and come from beds 7 through 10 (Fig. 15). Four specimens have been found ex situ, but two of them have been tentatively referred to a specific bed—beds 9 and 10, respectively. Over half of the material (56.52%) is collected from bed 8. It is likely that some of the indeterminate microvertebrate bones also belong to lissamphibians, and thus, the actual abundance of this clade is probably underestimated. Lissamphibians show strong preferences for freshwater and/or terrestrial environments (Duellman & Trueb, 1994), so their presence in brackish or marine deposits is indication of transportation of the remains. However, due to the small size and fragility of the bones, we hypothesize that the transportation was short and animals inhabited freshwater bodies close to the place of deposition, i.e. it is possible that they are a para-autochthonous element of the fossil assemblage.

##### Testudines

**Fig. 16:**
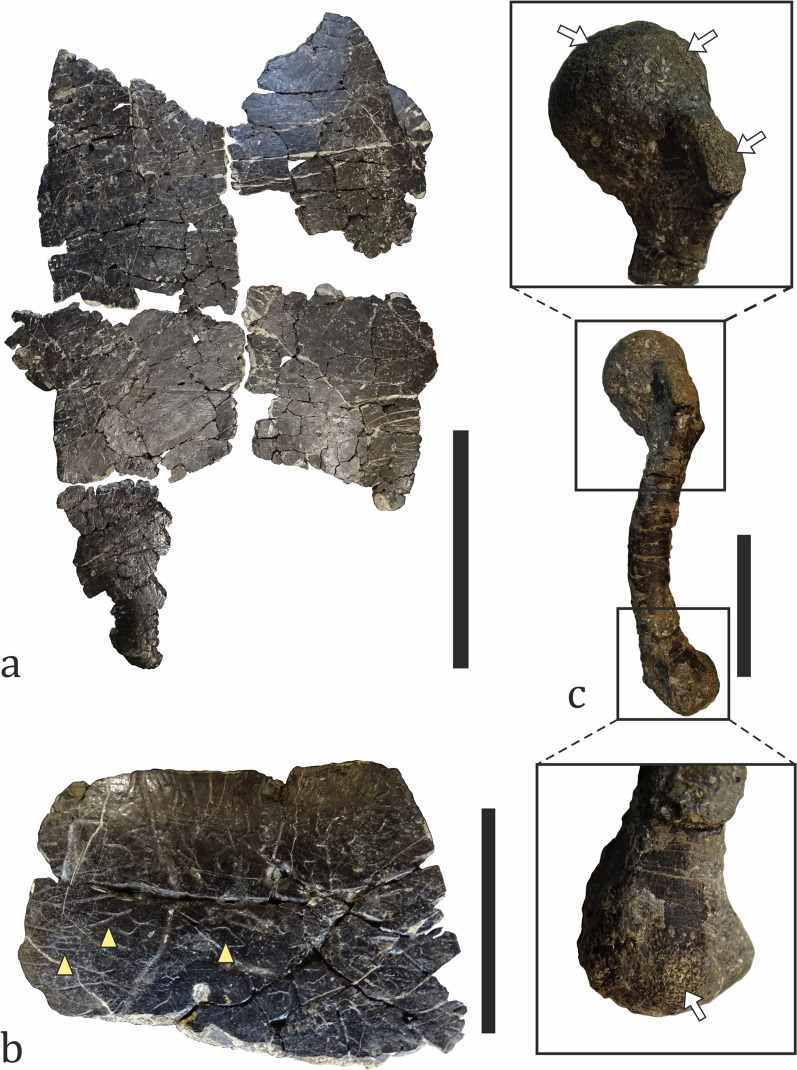
Turtle fossil material. **a** A partial plastron of bothremydid turtle (NMNHS FR26). **b** A carapace fragment, part of specimen NMNHS FR26, showing “pelomedusoid”-type surface ornamentation (pale yellow arrowheads). **c** A complete humerus (NMNHS FR51) with signs of abrasions in proximal and distal areas (arrows). Scale bar: **a**–100 mm; **b**, **c**—30 mm

Turtle remains are the most common vertebrate fossils at Vrabchov Dol, comprising 31.60% (n = 79) of all collected specimens (Fig. 13a). With the exception of a complete right humerus, all other fossils are either partial plastra and larger pieces of carapaces, or indeterminate shell fragments (Fig. 16). Fossils are found in beds 4 through 11 (Figs. 3, 15). From bed 10, 22 specimens have been collected, which amounts to 27.85% of all turtle material at the site (Fig. 15). At least two taxa are present at the site: one whose shell surface bears ornamentation of fine, diverging and irregular grooves and another one without ornamented shell surface. The shell ornamentation in the former is of the “pelomedusoid”-type observed in bothremydid pleurodirans (Gaffney et al., 2006) (Fig. 16b). The presence of bothremydines at the site has been previously mentioned in passing by Nikolov et al. (2020a); anatomical description of the most complete specimen is in advanced stage of preparation (L. Hristova, pers. comm.). Because members of Bothremydidae inhabited both freshwater and coastal marine environments during the Late Cretaceous (de Lapparent de Broin & Werner, 1998), we tentatively consider the studied material to be a para-autochthonous element of the fossil assemblage. The lack of evidence for prolonged transport (i.e. abrasion and rounding) in larger and better preserved shell fragments is in support of this interpretation. Posterior shell fragments attributable to the second turtle taxon found at Vrabchov Dol do not exhibit signs of pelvic elements sutured to the carapace and the plastron, an anatomical feature which is typical for pleurodires (Wise & Stayton, 2017). Although it cannot be identified on a lower taxonomic level, pending the discovery of more complete and/or diagnostic material, the absence of sutures for the pelvic girdle suggests that this taxon may be representative of the clade Cryptodira.

##### Crocodylomorpha

The clade Crocodylomorpha is currently represented only by fossil teeth, which account for 9.20% of all collected material (n = 23) (Fig. 13a). Of these, 65.22% (n = 15) are stratigraphically well-constrained, while 13.04% (n = 3) are tentatively referred to a particular bed, and 21.74% (n = 5) were found ex situ. Beds 5, 6, 8–11 yielded crocodylomorph teeth, with two to five specimens per bed (Fig. 15). Three teeth of identical morphology collected from bed 5 are ascribed to Allodaposuchidae by Hristova (2020) (Fig. 14j, k). Further field work revealed more crocodylomorph tooth morphotypes, which suggests that at least one additional taxon besides Allodaposuchidae is present at the site (Nikolov, pers. obs.) (Fig. 14l–n). The teeth of the second taxon are very similar in morphology to the teeth of hylaeochampsid eusuchians, like *Acynodon* and *Iharkutosuchus* (Delfino et al., 2008; Muscioni et al., 2024; Ősi & Weishampel, 2009; Ősi, 2008a). Because allodaposuchids and hylaeochampsids are semi-aquatic, predominantly freshwater animals, we consider the remains found at Vrabchov Dol to be of allochthonous nature. However, the recently described *Allodaposuchus palustris* expanded the paleoecology of allodaposuchids to coastal wetlands (Blanco et al., 2014). This leaves open the possibility that at least some of the crocodylomorph material is para-autochthonous. Lastly although allodaposuchids are best represented in the upper Campanian–Maastrichthian successions of Western Europe and Romania (Blanco, 2021), the oldest fossils (isolated teeth) referred to Allodaposuchidae to date come from the Santonian of Hungary (Ősi et al., 2016). In this chronostratigraphic context, the Vrabchov Dol allodaposuchid material constitutes one of the oldest records of the clade in Europe.

##### Pterosauria

**Fig. 17:**
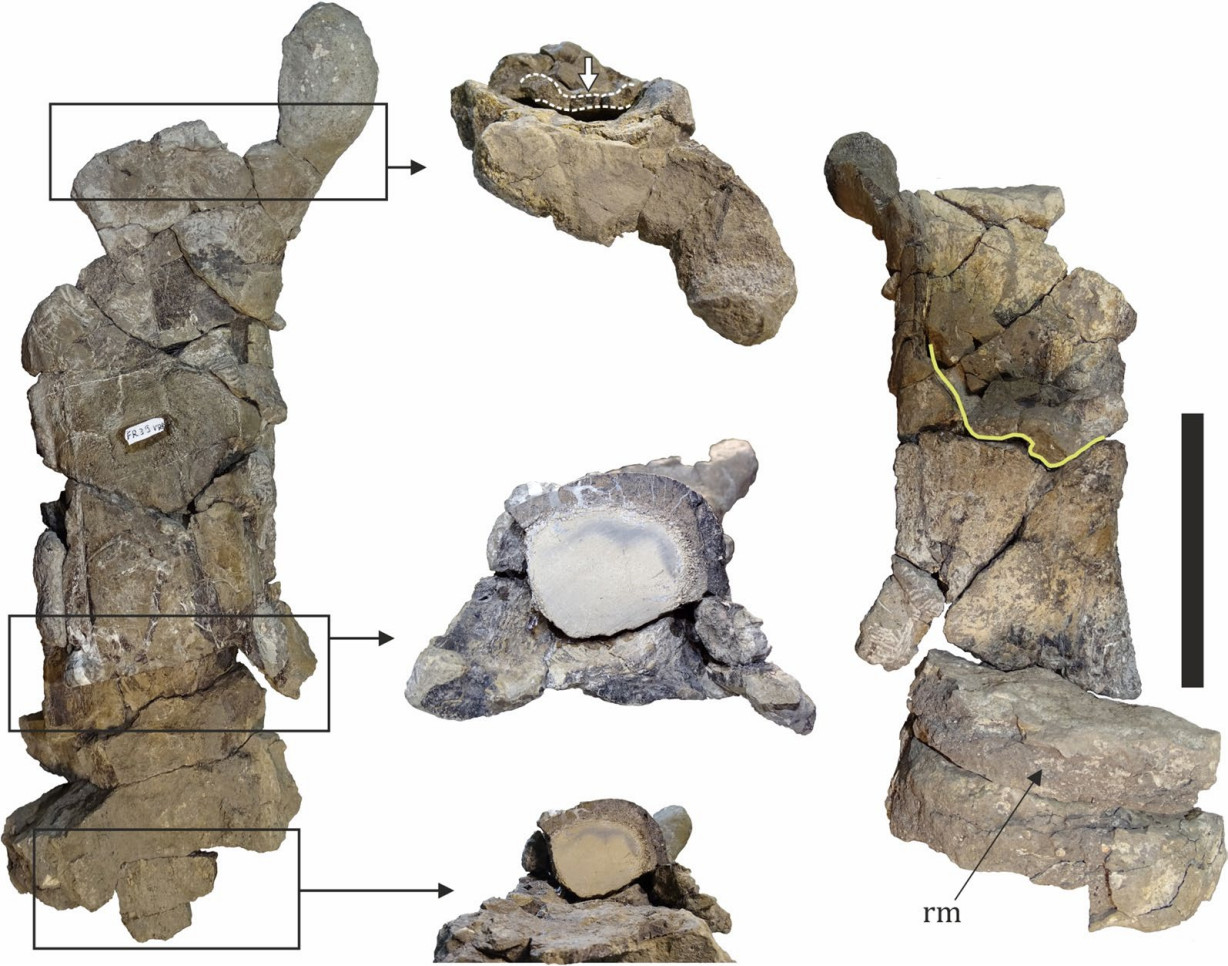
A putative pterosaur bone (NMNHS FR39). Solid yellow line (right) and arrow (top center) mark breakage and collapse of the bone wall within the medullar cavity, respectively. *rm* rock matrix. Scale bar: 100 mm

A single problematic bone is herein tentatively ascribed to Pterosauria (Fig. 17). The specimen (NMNHS FR39) is a fragment of a poorly preserved and partially crushed large-sized bone with a very large hollow medullar cavity. It is elongated and weakly rectangular in cross-section; the preserved length is 335 mm, while the width of the “shaft” along the long axis varies between 95 and 112 mm. The thickness of the cortex, as measured along broken surfaces, ranges from 5 to 12 mm, although locally it reaches up to 19 mm. The only more prominent morphological feature of the bone is a process, which extends above/below the cylindrical body, flares laterally and terminates in what appears to be a globular surface. NMNHS FR39 was excavated from bed 8 in 2018 and originally interpreted, while still entombed in hard sediment matrix, as a partial bone of a large theropod by Nikolov et al. (2018) due to its hollow interior and thin cortex. Later, after it was prepared, Nikolov et al. (2020a) reinterpreted the fossil bone as a very large pterosaur humerus, with the globular surface at the process extremity regarded as an incomplete humeral capitum. However, after a direct comparison with the holotype of the giant Romanian pterosaur *Hatzegopteryx thambema* Buffetaut et al., 2002a, which includes a partial humerus (Buffetaut et al., 2002a), the validity of the ‘humerus-hypothesis’ was challenged on the grounds of morphological differences and significant discrepancies in cortical bone thickness (Nikolov, pers. obs.). If the discussed specimen is indeed pterosaurian in origin, then its size and age suggest that it belongs to an azhdarchid pterosaur. The partially preserved process of NMNHS FR39 is morphologically reminiscent of the prezygapophysis of azhdarchid cervical vertebrae, but the observed cortical thickness at various parts of the bone is unusual for a pterosaur. Despite similarities with some published in the literature specimens (e.g., Vremir et al., 2015), direct comparisons with azhdarchid vertebrae are necessary to resolve the taxonomic identity of the fossil. We studied the osteohistology of this specimen and information on its ontogenetic state and complex taphonomic history is provided later in the text.

##### Dinosauria

**Fig. 18:**
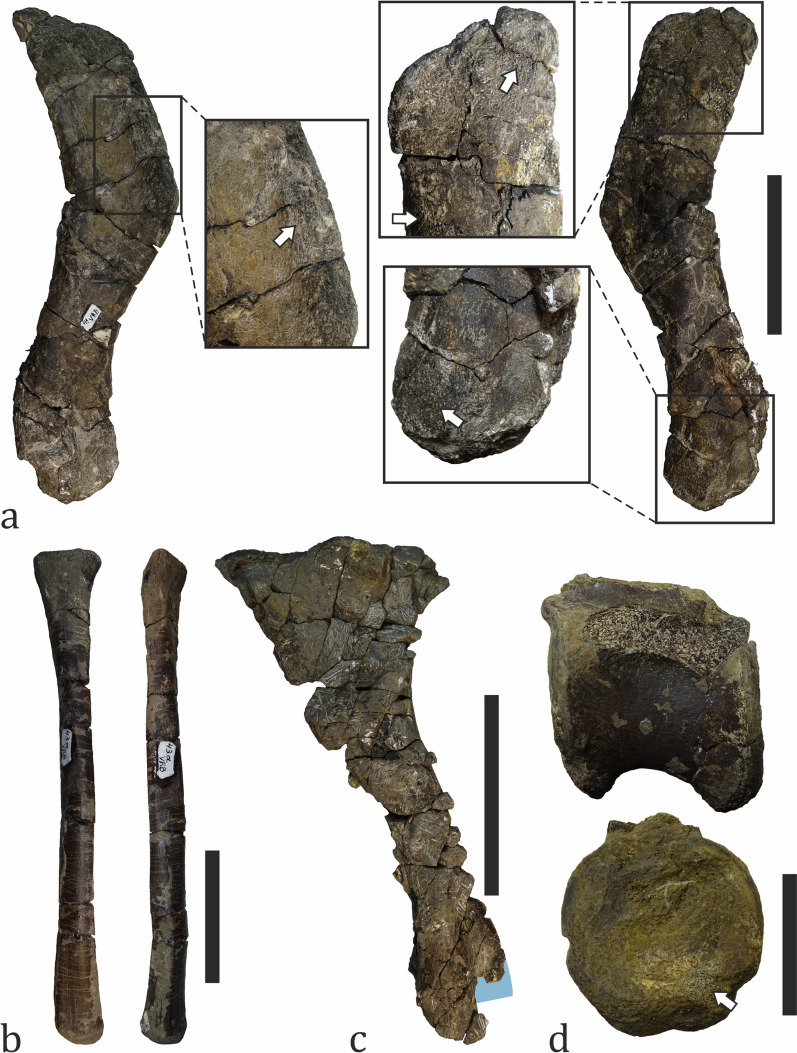
Ornithopod fossil material.** a** A complete left humerus (NMNHS FR41) in anterior (left) and posterior (right) view, with signs of abrasion (marked with arrows in enlarged areas). **b** A complete right radius (NMNHS FR43a) in medial (left) and anterior (right) view. **c** Almost complete left tibia (NMNHS FR40) in lateral view. The blue area on the posterior side marks the paleohistology sampling location. **d** A caudal centrum (NMNHS FR47) in left lateral (up) and posterior (bottom) view. The arrow marks an area suffering abrasion. Scale bar: **a**, **c**—100 mm; **b**—50 mm; **d**—30 mm

**Fig. 19:**
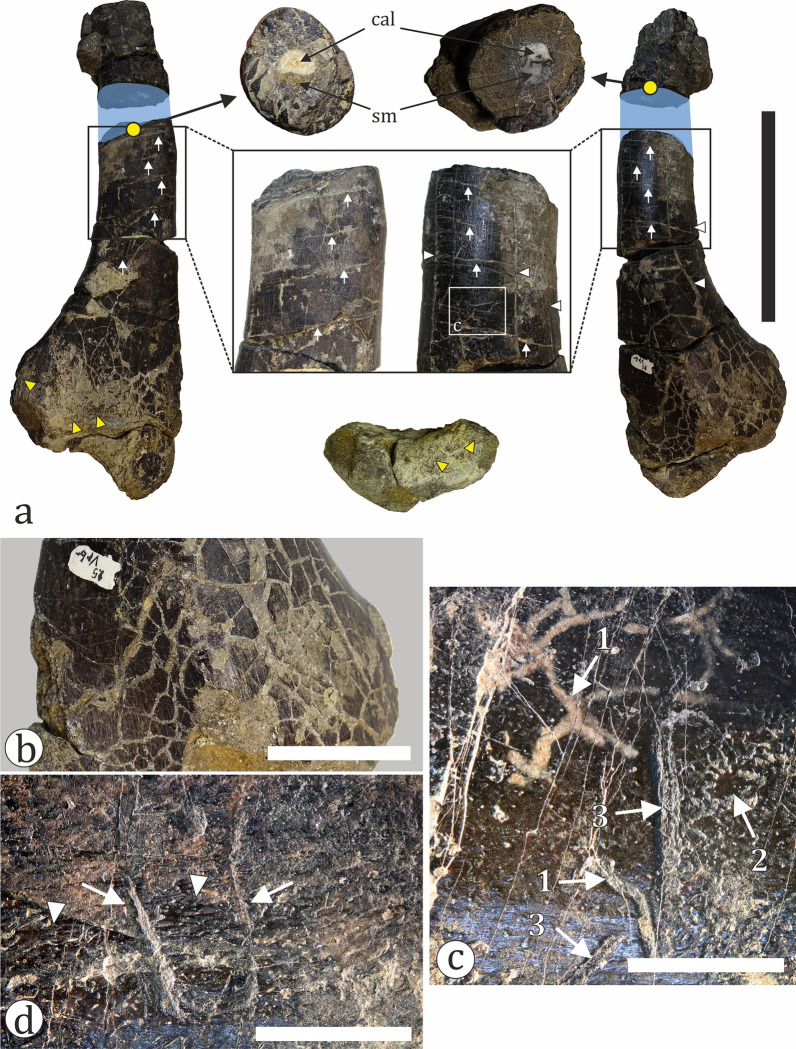
Ornithopod fossil material. **a** Partial left tibia (NMNHS FR25) in anterior (left), posterior (right) and distal (bottom) view. The blue colored area marks the position of the paleohistological sampling. Yellow arrowheads mark bone areas with signs of abrasion. White arrows mark fractures of diagenetic origin, while arrowheads mark weathering cracks. **b** The posterior surface of tibia NMNHS FR25 showing numerous weathering cracks. **c** A detail of the posterior diaphyseal surface which bears several types of bioerosional structures, including furrows/canals (1), shallow borings forming dendrite-like structures (2) and possible tooth marks (3). **d** A detail of the posteromedial diaphyseal surface showing possible tooth marks (arrows). White arrowheads indicate abraded areas where the underlying cortical vascular network is revealed on the surface. *cal* calcite, *sm* sediment matrix. Scale bar: **a**—100 mm; **b**—30 mm; **c**, **d**—5 mm

**Fig. 20:**
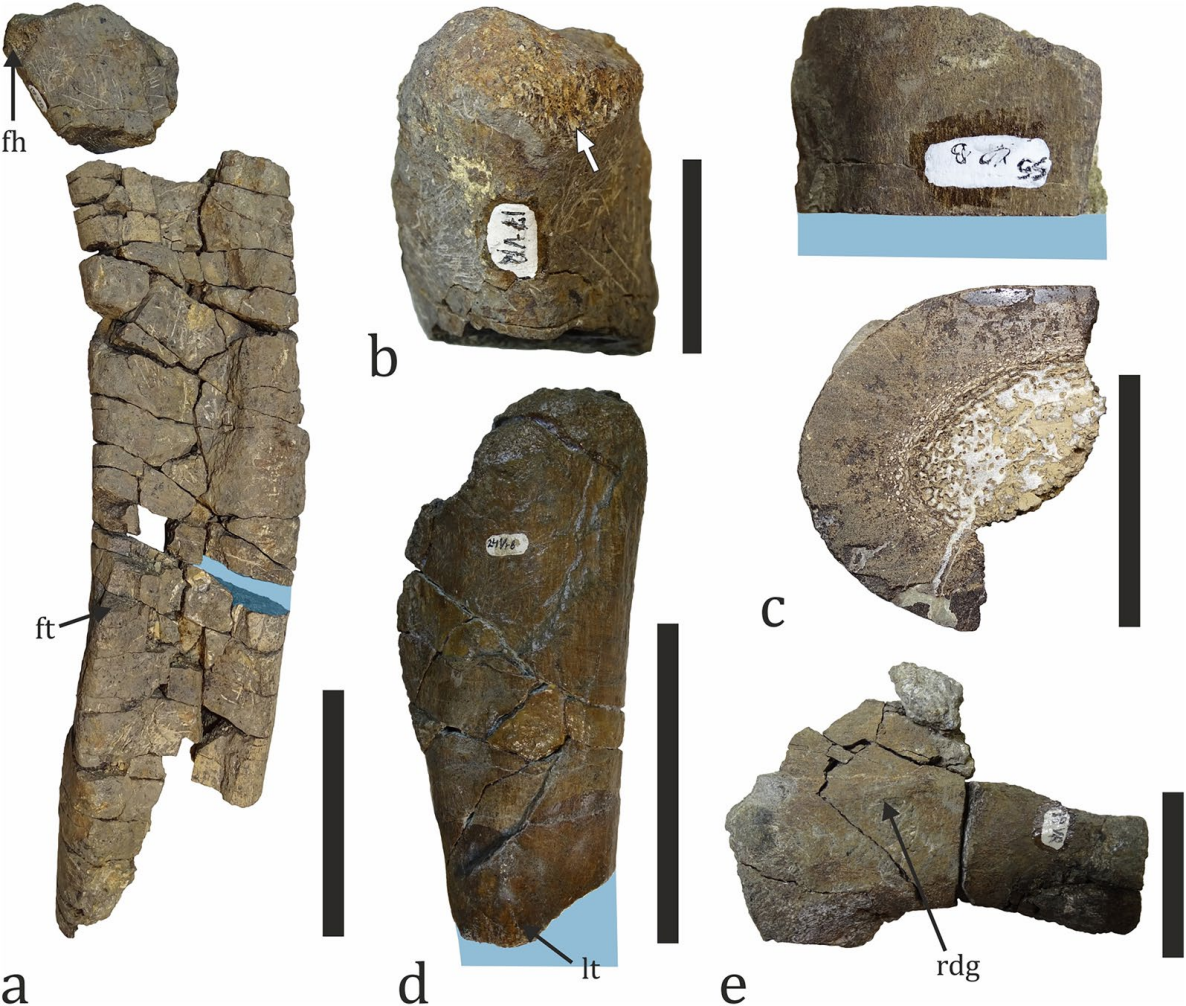
Titanosaur fossil material. **a** Partial right femur (NMNHS FR17) in posterior view. The blue area marks the position of the paleohistological sampling. **b** The femoral head of NMNHS FR17 in medial view, showing an abraded surface (arrow). **c** Stylopodial diaphyseal bone fragment (NMNHS FR55). The blue area marks the position of the paleohistological sampling. **d** Proximal fragment of a fibula (NMNHS FR24) in lateral view. The blue area marks the position of the paleohistological sampling. **e** Partially preserved caudal centrum (NMNHS FR18) in left lateral view. *fh* femoral head, *ft* fourth trochanter, *lt* lateral trochanter, *rdg* ridge. Scale bar: **a**, **d**—100 mm; **c**—50 mm; **b**, **e**—30 mm

**Fig. 21:**
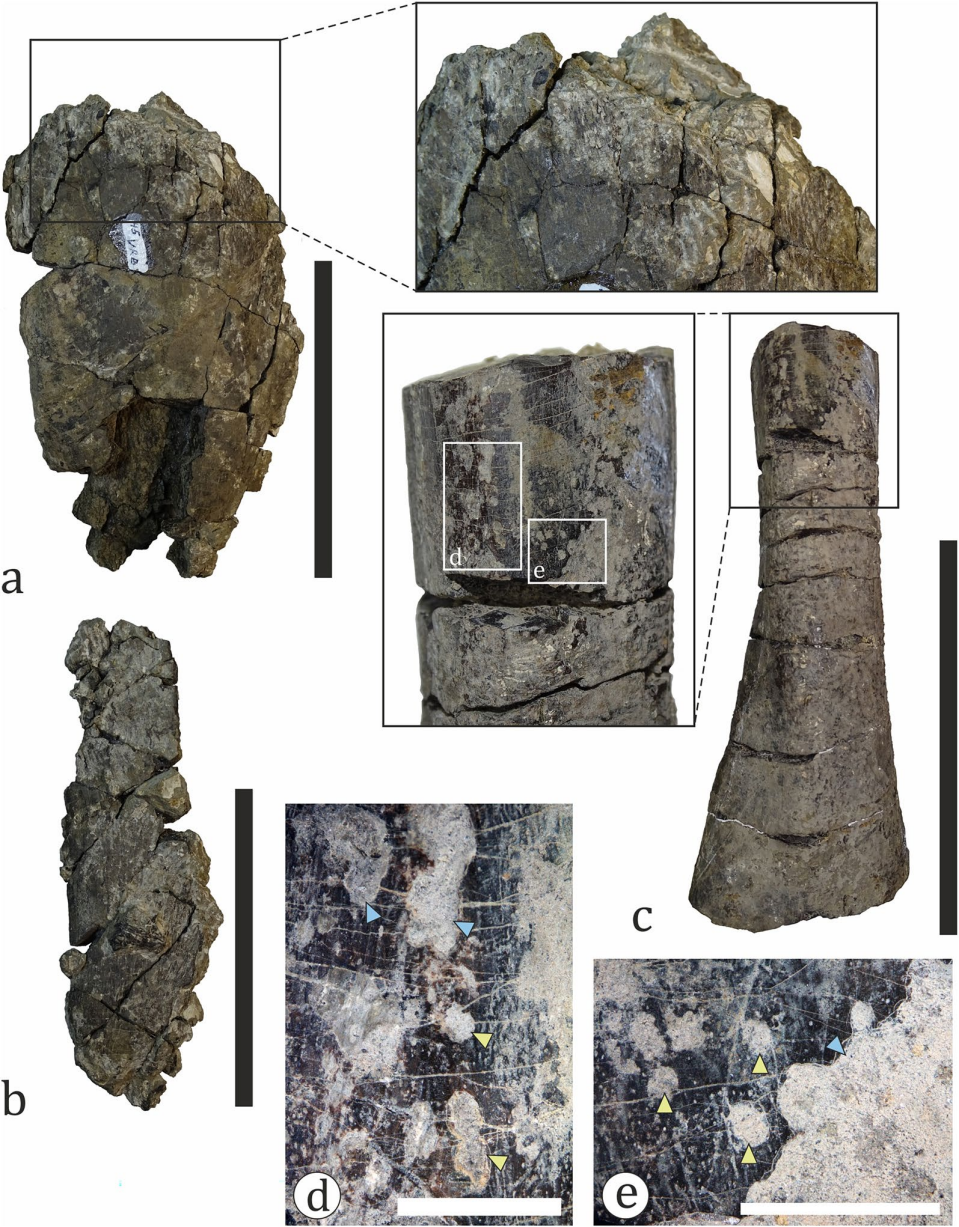
Titanosaur fossil material. **a** Proximal fragment of a fibula (NMNHS FR45) in lateral view. The enlarged area shows the poor state of preservation and abrasion of proximal parts of the bone. **b** Cortical bone fragment which is found in proximity and assumed to be part of specimen NMNHS FR45. **c** Partial tibia (NMNHS FR44) in posterior view. **d, e** Bioerosion on the posterior surface of the diaphysis of NMNHS FR44. Yellow arrowheads mark single boreholes and blue arrowheads mark groups of boreholes which locally completely destroy the original bone surface. Scale bar: **a**–**c**: 100 mm; **d**, **e**: 5 mm

**Fig. 22:**
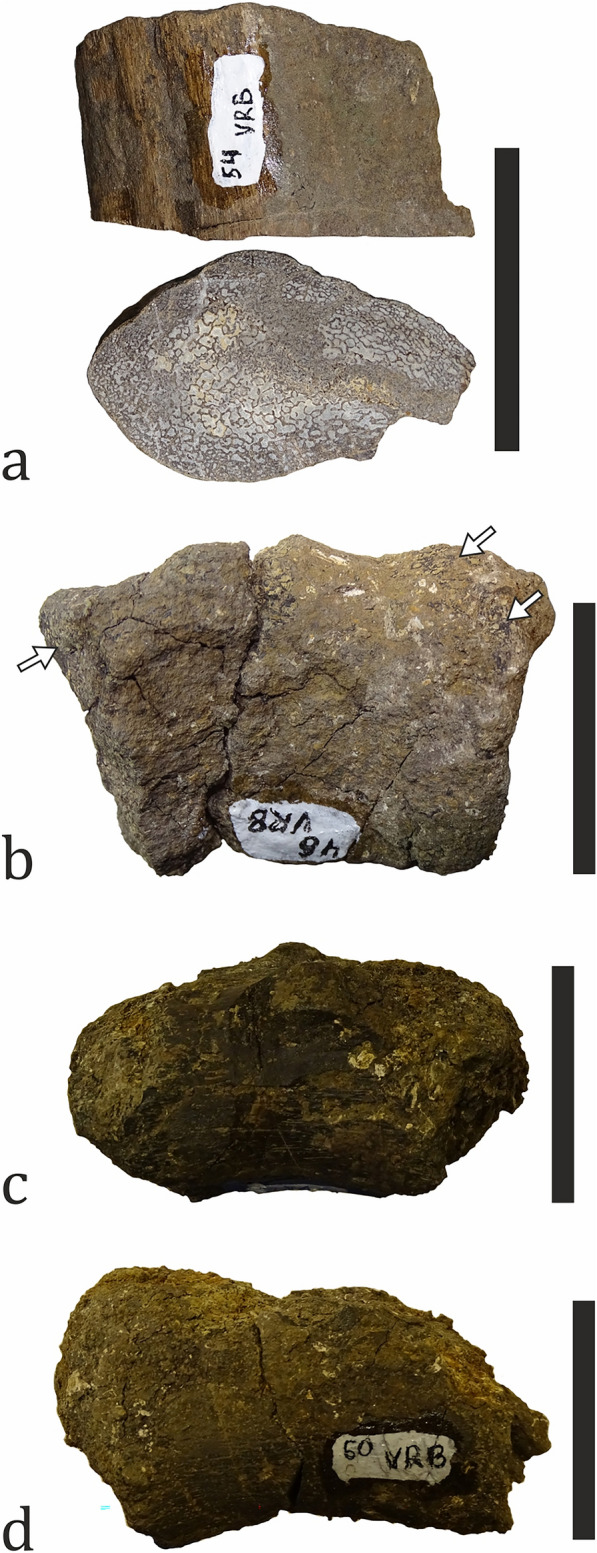
Indeterminate dinosaur material. **a** Long bone fragment (NMNHS FR54). **b–d** Poorly preserved centra (NMNHS FR46, FR63, FR60). Scale bar: **a: **50 mm; **b**–**d**: 30 mm

The fossils of non-avian dinosaurs are the second most common vertebrate fossils at Vrabchov Dol. They comprise 18% (n = 45) of all fossil material collected so far and 23.56% of the taxonomically ascribed specimens (Fig. 13), and include axial (vertebrae and one prezygapophyseal fragment) and appendicular elements (a femur, tibiae, fibulae, humerus, radius, ulna, and metapodia), as well as indeterminate bone fragments (Figs. 18, 19, 20, 21, 22). Twenty-two specimens (48.89%) have been found ex situ, of which three (6.67%) could be tentatively assigned stratigraphically. The in situ collected bones originate from beds 5, 7–11, with bed 9 being the most productive one with 11 specimens (24.44%) and one more specimen tentatively referred to it. The dinosaur fossils appear to be concentrated in beds 8 through 10, where 44.44% (n = 20) of the material has been found. Three of the fossil-bearing beds yielded only one specimen each (Fig. 15). The fossils are predominantly fragmentary, disarticulated and, with a single exception, found in isolation. The degree of preservation varies from poor to relatively good, although better preserved specimens are rare. In addition, some of the bones have suffered diagenetic deformation. At least two dinosaur clades—Ornithopoda and Titanosauria—are currently recognized as part of the fossil assemblage. The undiagnostic and as of yet undetermined specimens cannot be confidently ascribed to one of these two clades (with the exception of few specimens identified as titanosaurian on the basis of their osteohistology), but are here provisionally referred to as Dinosauria indet. due to their size, robustness, diaphyseal internal structure (i.e. cortical thickness and size and characteristics of the medullary area) and, in some cases, bone histology.

Ornithopod remains (n = 7) account for 2.80% of all vertebrate fossils and for 15.56% of the dinosaur material. The specimens are a complete humerus, associated complete radius and an ulna missing the mid-diaphyseal portion, one tibia missing the distal epiphysis and another one comprising the distal half of the bone, incomplete fibula and a caudal centrum (Figs. 18, 19). Ornithopod fossils are found in beds 5, 9, and 10, with bed 9 being the most productive one with 4 specimens. The centrum is collected ex situ. Based on the stratigraphic distribution of the material we infer that the bones belong to at least three, but probably four different individuals; although all forelimb elements come from a laterally restricted section of bed 9, there is apparent size discrepancy between the humerus and the lower arm bones, which indicates that they might belong to different animals. Two groups of ornithopods are known to have been present on the European Archipelago during the Santonian–early Campanian interval—rhabdodontids and hadrosauroids (Augustin et al., 2023b; Chiarenza et al., 2021). Although we refrain from ascribing the material to any of these groups with certainty pending a detailed osteological study, it is worth mentioning that the presence of hadrosauroids in the assemblage is indicated by two specimens – humerus NMNHS FR41 and centrum NMNHS FR47 (Fig. 18a, d). The humerus is S-shaped and has a long and well-developed deltopectoral crest which extends distally to around mid-shaft (its length is estimated to be about 43% of the total length of the bone, but the poor preservation of the proximal part of the bone hinders more precise measurement), as is typical for hadrosauroids (Horner et al., 2004). Additionally, in terms of general morphology it is very similar to a recently described hadrosaurid humerus from the upper Campanian of Romania (Ebner et al., 2025). The caudal centrum also has typical hadrosauroid features, namely a hexagonal outline in anterior (cranial) and posterior (caudal) view (Horner et al., 2004). It should be noted that the size of the remaining Vrabchov Dol ornithopod bones is larger than that of correlative elements in quasi-contemporaneous (Santonian–early Campanian) rhabdodontid species *Mochlodon vorosi* and *Mochlodon suessi* from Hungary and Austria, respectively (Ősi et al., 2012a), and closer to that of large rhabdodontids from the lower Campanian of France and *Tethyshadros* from the lower Campanian of Italy (Buffetaut et al., 1996; Chiarenza et al., 2021). Furthermore, our histological analysis (see below) reveals that despite the size difference, both tibiae (NMNHS FR25, FR40) belong to subadult individuals, and thus, it is possible that there is more than one ornithopod taxon at the site (Figs. 18c, 19a). Ornithopods inhabited diverse terrestrial habitats, but many appear to be associated with coastal environments (Butler & Barrett, 2008; but see Vázquez López et al., 2025). We consider the Vrabchov Dol ornithopod fossils to be para-autochthonous or allochthonous elements of the fossil association.

Nikolov et al. (2020b) first hypothesized the presence of titanosaurs on the basis of the osteohistology of two indeterminate bone fragments found in the late 2000s and in 2017, respectively. Subsequent excavational campaigns led to the discovery of several fragmentary, but diagnostic sauropod axial and appendicular elements, including caudal vertebrae, a femur, two partial fibulae, and a metatarsal, among others (Figs. 20, 21). After the extinction of rebbachisaurids in the Turonian (Salgado et al., 2022), titanosaurs remained the only Late Cretaceous group of sauropods globally. This fact allows us to provisionally ascribe all remains to Titanosauria, pending a more detailed description. Currently, the titanosaurian material (n = 9) comprises 3.6% of all discovered fossils and 20% of the dinosaur specimens. Four bones were collected from beds 8 and 9 (two bones per bed), two other specimens could be tentatively referred to bed 8, and the rest were found ex situ. The fossil material pertains to at least two individuals. Skeletal elements are incompletely preserved, found in isolation, and bear macro- and microscopic signs of abrasion (see below). Although there are titanosaurs known from paralic environments (e.g., Augustin et al., 2023a; Smith et al., 2001) and recent studies reveal that at least some latest Cretaceous European titanosaurs entered coastal environments like lagoons or marginal freshwater wetlands (Marmi et al., 2014; see also Vázquez López et al., 2025), traditionally, the members of this sauropod clade have been considered to be inhabitants of inland environments (Butler & Barrett, 2008; Mannion & Upchurch, 2010; Vázquez López et al., 2025). In the case of the Vrabchov Dol titanosaurs, the nature of the fossil material suggests that these animals lived at some distance from the depositional center, and thus, we deem their fossils to be an allochthonous component of the assemblage.

### Osteohistology and microscopic features of the bones

#### Osteohistology and ontogenetic stage

We sampled 11 dinosaur fossils for histological analysis (Table 1). These account for 24.44% of all recognized dinosaur material (n = 45). Overall, the degree of bone tissue preservation is moderate to good and without extensive microorganism influenced diagenetic alterations of the cortical bone, which allows for reliable histology-based ontogenetic study of the material.

**Fig. 23:**
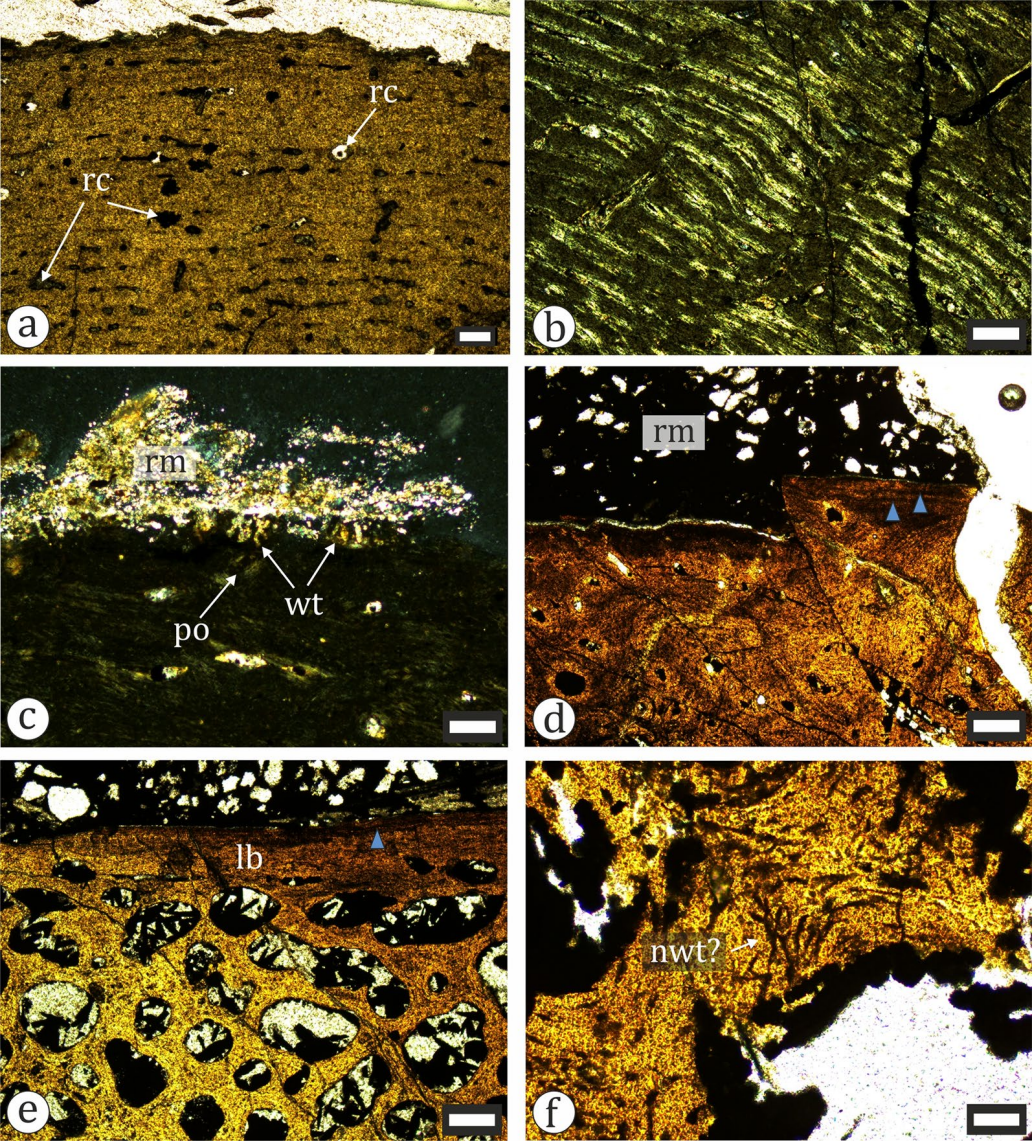
Osteohistology of ornithopod tibiae in plane- (**a**, **d**-**f**) and cross-polarized (**b**, **c**) light. **a** Laminar bone in the outer cortex of NMNHS FR25. **b** Laminar bone in the mid-cortex of NMNHS FR25. **c** Wedl tunnels on the bone surface of NMNHS FR25. **d** The cortex of NMNHS FR40 showing poorly vascularized lamellar bone with two lines of arrested growth (blue arrowheads). **e** The thin cortex of lamellar bone and extensive trabecular bone region of NMNHS FR40. **f** Trabecular bone in NMNHS FR40 affected by microbial microscopic focal destruction. *lb* lamellar bone, *nwt* non-Wedl tunnel, *po* primary osteon, *rc* resorption cavity, *rm* rock matrix, *wt* Wedl tunnel. Scale bar: **a**, **b**, **d**, **e**—250 μm; **c**—100 μm; **f**—50 μm

Our sample includes two ornithopod tibiae—NMNHS FR25 and FR40. The larger of the two (NMNHS FR25) is sampled at about mid-diaphyseal level (Fig. 19a). The cortex is thick and surrounds an oval medullar cavity which is free of trabecular bone. Primary bone tissues are highly vascularized and of the woven-parallel complex (WPC) type. The amount of parallel-fibered tissue in the bone matrix varies in different parts of the section. Vascular canals are longitudinal and circumferential primary osteons (PO) and arranged in laminar fashion (Fig. 23a–b). PO are mature, even in the outer cortex where there appears to be a slight decrease in vascularization. The secondary remodelling is generally weak, with only the inner third of the cortex more affected by resorption and secondary osteon (SO) formation. There, two generations of SO are observed, but these do not form Haversian bone tissue. SO and resorption cavities in the outer half of the cortex are relatively rare (Fig. 23a). It is difficult to ascertain the number of growth marks present in the bone, but there appear to be two lines of arrested growth (LAGs) in the outer cortex. Three circumferential cracks in the inner half of the bone wall, which can be traced along the section, might be the result of breakage along existing LAGs. The periosteal surface is poorly preserved and there are no signs of an external fundamental system (EFS) (Fig. 23c). The overall histology is typical for subadult ornithopods (Horner et al., 2000; Hübner, 2012; Prondvai, 2014; Werning, 2012). Specimen NMNHS FR40 is sampled distally, at the posterior side of the metaphysis (Fig. 18c). This sampling location makes the ontogenetic interpretation of the bone histology more difficult. The compacta is thin (Fig. 23d, e), composed of an outer layer of lamellar bone underlain by small amounts of poorly vascularized WPC tissues with predominantly parallel-fibered matrix. Longitudinal simple vascular canals are the most common, while PO are rare. Under the primary tissues there is a zone of trabecular bone (Fig. 23e), where some of the trabeculae consist of endosteal lamellar bone. The cortical periphery contains two LAGs (Fig. 23d). We tentatively consider this bone to belong to a subadult, or possibly even an adult individual.

**Fig. 24:**
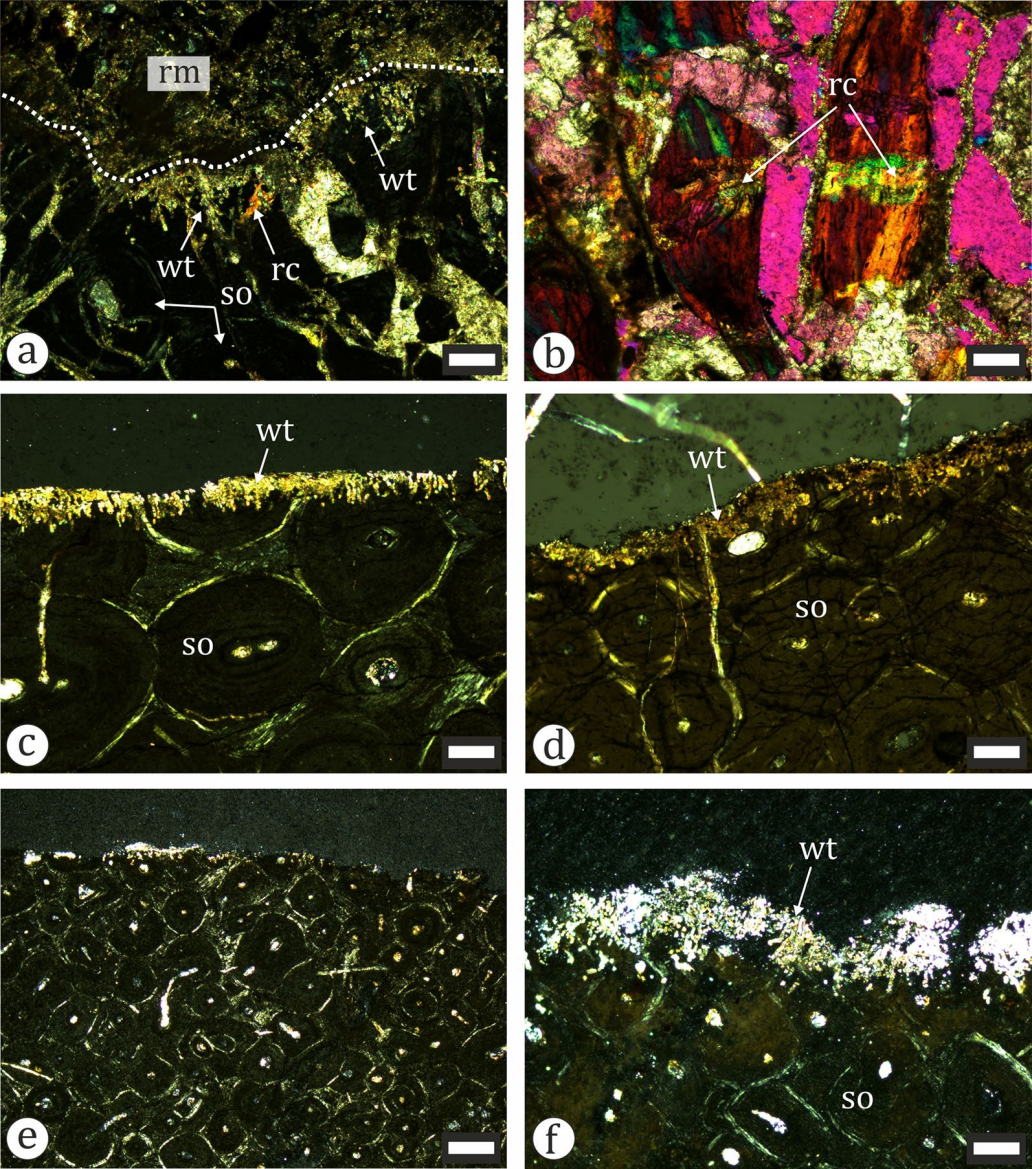
Osteohistology of titanosaur bones in cross-polarized light. **a** Secondary osteons and Wedl tunnels in the outermost cortex of NMNHS FR17. The dotted line traces the bone surfaces. **b** Partially recrystallized bone tissue in perimedullary developed bone trabeculae in NMNHS FR17 (λ-filter is used). **c, d** Numerous secondary osteons (**c**) and Haversian bone (**d**) in the outermost cortex of NMNHS FR24. Note the presence of Wedl tunnels along the bone surface and how the latter cuts deeply into the peripherally positioned secondary osteons. **e, f** Extreme cortical bone remodelling and local formation of Wedl tunnels in NMNHS FR55. *rc* recrystallization, *rm* rock matrix, *so* secondary osteons, *wt* Wedl tunnels. Scale bar: **a**–**d**, **f**—100 μm; **e**—250 μm

All five titanosaur specimens exhibit typical bone histology for the clade, which is characterized by highly vascularized primary tissues of the WPC with increased amounts of parallel-fibered or lamellar component in the bone matrix, laminar vascularization, strong to extreme bone remodeling with formation of dense Haversian bone, and few, if any, growth marks in the cortex (Klein et al., 2012; Sander et al., 2011) (Fig. 24). The HOS of the fossils ranges from HOS 10–11 for the femur NMNHS FR17 (Fig. 20a) to HOS 14 for long bone fragment NMNHS FR16 (the osteohistology of the latter specimen is described in detail by Nikolov et al., 2020b). Specimen NMNHS FR17 has also the lowest RS in the sample with a value of RS 4. The RS of the other specimens varies from RS 9 in fibula NMNHS FR24 to RS13 in long bone fragment U.S., K2 1586 (Table 1). The latter specimen is also the only one to have a LAG in the outermost cortex (see Nikolov et al., 2020b). Because none of the fossils shows evidence of pronounced decrease in vascularity or closely spaced growth marks in the outer cortex indicative of significantly slowed down bone growth rates, or the formation of EFS, which signifies the attainment of skeletal maturity, all of them are considered to belong to subadult individuals. The partial titanosaurian femur NMNHS FR17, which has a preserved length of 382 mm and hypothesized total length of about 500 mm, shows the youngest bone histology, although this may be the result of the overall faster growth of stylopodial elements, the femur in particular, compared to other limb bones (Padian et al., 2016). Because NMNHS FR17 comes from an island-dwelling sauropod, it is worth noting that similarly sized femora of the island-dwarf titanosaur *Magyarosaurus dacus* exhibit more advanced HOS than this specimen (Stein et al., 2010).

**Fig. 25:**
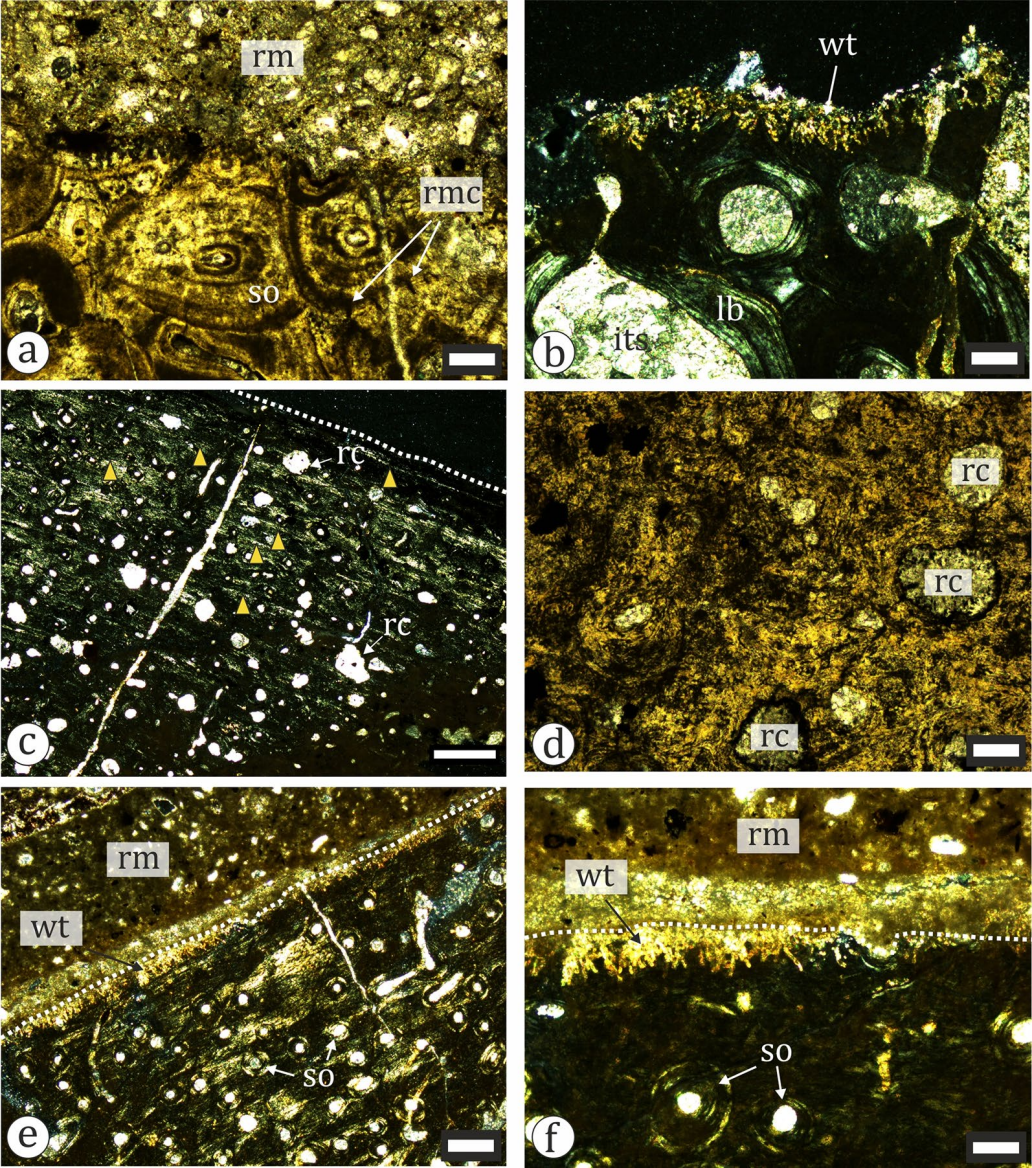
Osteohistology of taxonomically indeterminate dinosaur bones and a putative pterosaur bone in plane- (**a**, **d**) and cross-polarized (**b**, **c**, **e**, **f**) light. **a** Haversian bone in the outer cortex of metapodial NMNHS FR23. **b** Deeply eroded inner cortical and trabecular bone with Wedl tunnels in NMNHS FR54. **c** Optically anisotropic primary cortical tissues of bone fragment NMNHS FR21 with resorption cavities and at least 6 lines of arrested growth (yellow arrowheads). **d** Part of the deep cortex of NMNHS FR21 strongly affected by microbial focal destruction of the bone tissue. **e, f** Bone histology of the outer cortex of a putative pterosaur bone NMNHS FR39. *its* intertrabecular space, *lb* lamellar bone, *rc* resorption cavity, *rm* rock matrix, *rmc* radial microcracks, *so* secondary osteons, *wt* Wedl tunnels. Scale bar: **a**, **b**, **d**, **f** 100 μm; **c**—500 μm; **e**—250 μm

Two of the indeterminate dinosaur bone fragments (NMNHS FR19 and FR54) proved to be uninformative in regards to establishing their ontogenetic stage due to not preserving any compact cortical bone. The cross-section of the indeterminate metapodial element (NMNHS FR23) reveals a moderately thick cortex surrounding a central area completely infilled by cancellous bone. The compacta is completely remodelled and no growth marks can be observed; at least three generations of SO form dense Haversian bone in the outer cortex (Fig. 25a). Such a degree of bone remodeling is typical for skeletally mature bones; however, because of the higher degree of secondary remodeling in smaller and/or more slowly growing skeletal elements we cannot exclude the possibility that NMNHS FR23 belongs to a subadult individual. Specimen NMNHS FR21, an indeterminate diaphyseal fragment, exhibits an osteohistology which is similar to that of ornithopod tibia NMNHS FR25. The primary tissues are of WPC type with laminar vascularization. In the outer third of the cortex the parallel-fibered and lamellar tissues predominate (Fig. 25c). It appears that the vascularization decreases periosteally. Numerous resorption cavities and secondary osteons are present throughout the thickness of the cortex, even in its outer third, but there is no formation of Haversian bone. Two annuli and between eight and ten LAGs are present (Fig. 25c). The two outermost LAGs are closely spaced and associate with lamellar bone, which we interpret as an incipient EFS.

In addition to the dinosaur fossils, we sampled a putative pterosaur bone (NMNHS FR39). It has a thin compact cortex with small amounts of cancellous to trabecular bone endosteally. The primary tissues are strongly anisotropic with lamellar bone tissue present for certain in the outer third of the cortex (Fig. 25e). It appears that the collagen bundles are oriented in two different directions locally—some bundles are organized circumferentially, while the rest are oblique to the periosteal surface (not in the sense of the ‘plywood-like bone’ of de Ricqlès et al., 2000). The characteristics of the vascular architecture are difficult to observe. Both longitudinal and circumferential PO are present, but they are very mature, almost completely filled by lamellar tissue. The outermost third of the cortex is poorly vascularized (Fig. 25e). The whole bone is affected by processes of remodeling as evidenced by the presence of SO in the outer cortex and subperiosteally. SO become more densely packed in endosteal direction, where, locally, there are up to three generations of them. There is no formation of dense Haversian bone. The periosteal surface is abraded deeply as evidenced by the cross-cutting relationship between the bone surface and SO, and thus, an EFS, if present at all, is not observed (Fig. 25f). Although we could not convincingly establish the presence of growth marks, the overall histomorphology of the specimen suggests that it might be skeletally mature. In general, the histology of NMNHS FR39 is within the histodiversity known in pterosaurs (de Ricqlès et al., 2000; Padian & Woodward, 2021). An argument against the interpretation of this specimen as a pterosaur bone element is the absence of medullary bony struts, which are characteristic for pterosaur vertebrae and long bones (de Ricqlès et al., 2000; Williams et al., 2021).

Our analysis reveals that among the osteohistologically studied specimens there are no very young juvenile individuals (Table 1). All titanosaur bones and one of the ornithopod tibiae (NMNHS FR25) exhibit subadult histology. Three other specimens, including the smaller ornithopod tibia (NMNHS FR40) and the putative pterosaur bone, can be convincingly interpreted as subadult, although we cannot exclude the possibility that they have reached skeletal maturity. Only specimen NMNHS FR21 shows clear histological characteristics indicative of significant slowdown or complete cessation of appositional bone growth.

There are three possible hypotheses explaining the absence of juvenile individuals in our sample: (1) age segregation in the dinosaur taxa present at the locality, with young animals inhabiting environments away from the coast, (2) taphonomic bias against the preservation of juvenile remains, (3) and collection and/or sampling bias. It is known by osteological, histological, and ichnological evidence that some species of dinosaurs, including sauropods and derived ornithopods (hadrosaurids), formed age segregated groups (Joubarne et al., 2024; Myers & Fiorillo, 2009; and references therein). However, the scarce, isolated and commonly fragmented nature of the dinosaur material at Vrabchov Dol in no way suggests that the remains represent individuals of a single population of titanosaurs or ornithopods. Instead, as supported by some micro- and macroscopic features of the fossil bones (see below), we concur that the studied specimens experienced a variable degree of transportation before their deposition. Although the paleohistological sampling was largely opportunistic, in order to reduce the sampling bias, we chose for analysis specimens which cover all sizes of dinosaur bones recognized so far–from 101 mm-long metapodial NMNHS FR23 to titanosaur femur NMNHS FR17 with estimated length of about 500 mm. In conclusion, we deem it most parsimonious that the absence of juvenile material bears a taphonomic signal and is probably the result of smaller and more fragile bones not surviving the process of transportation to the depositional basin.

#### Microscopic taphonomic features of the bones

Bioerosion and other biotic and abiotic histology related microscopic features of the fossil bones can be used to reconstruct their taphonomic history (Pfretzschner, 2004; Trueman & Martill, 2002; Turner-Walker & Jans, 2008). All the thin sectioned Vrabchov Dol specimens exhibit diagenetic fractures, which are infilled with mineral phases, most commonly calcite. The medullary region of bones and the intertrabecular spaces are predominantly filled by calcite, although in some of the fossils these contain different amounts of the marly host rock matrix, with occasional fragments of mollusc shells. In this regard, specimen NMNHS FR25 is interesting because its medullar cavity is half filled by a rock matrix, with the remaining space taken by diagenetic calcite (Fig. 19a). Because the boundary between the two types of mineral infilling reflects the horizontal surface, it is possible to deduce that upon the final burial the bone was lying on its posterolateral side. A black opaque optically isotropic phase infills osteocyte lacunae and vascular canals, but also lines medullar or trabecular surfaces, or occurs as discrete spots or irregular mass over the cortical tissues and within intertrabecular spaces (Fig. 23a, d–f, 25a). Notably, this mineral phase is particularly abundant in tibia NMNHS FR40, where it forms large elongated crystals not observed in any other sampled specimen (Fig. 23e). In some of the thin sections areas of recrystallized cortical tissues are present (Fig. 24b). SO radial microcracking, which is characteristic for the fossilization of Haversian bone in aquatic environments (Pfretzschner, 2004), while present in all secondarily remodelled specimens, appears to affect relatively few SO. The only exception is metapodial NMNHS FR23, in which microcracking is common (Fig. 25a).

The Histology Index (HI), as introduced by Hedges et al. (1995), ranges from 2 to 5 (Table 1). NMNHS FR21 is most affected by the process of microscopic focal destruction (HI = 2), while 41.70% (n = 5) of the sampled bones show little evidence for intracortical bioerosion (HI = 4). In 25% of the specimens (n = 3) different portions of the cortex exhibit HI between 3 and 4.

Several types of microscopic bioerosion are observed in the sample, but the most common by far are tunnel-like peripherally positioned structures, which morphologically correspond to the Wedl tunnels of Hackett (1981) (Figs. 23c, 24, 25). These structures are present in eight out of the 12 histologically studied bones. The tunnels are simple or distally branching and extend from the bone outer surface inwards. They may occur individually, but more frequently are numerous. These borings are about 10 μm in width and extend up to 100–150 μm into the cortex. Within the thin section, the tunnels are not uniformly formed along the bone periphery, possibly reflecting the extent of the conditions which favoured the colonization of bone by microorganisms. The formation of Wedl tunnels (or similar bioerosional structures), which were first observed by Wedl (1864) in fresh human teeth, has traditionally been attributed to the activity of fungi (Hackett, 1981; Marchiafava et al., 1974; Wedl, 1864), but more recently Davis (1997) and Turner-Walker (2019) proposed euendolithic microflora, like cyanobacteria or chlorophytes, as bioerosional agents.

In addition to Wedl tunnels, specimen NMNHS FR55 exhibits bone tissue focal destruction structures morphologically similar to those reported by Bell and Elkerton (2008) and Pesquero et al. (2010) in recent human remains from marine environment and in Miocene fossil bones from lacustrine environments, respectively (Fig. 24f). Non-Wedl tunnels are not particularly wide-spread, both within a particular thin section and across specimens. Most commonly, they are observed around SO, but also within trabeculae and interstices of primary bone tissue (Figs. 23f, 25d). The progressive deterioration of cortical bone tissues and formation of non-Wedl tunnels has been hypothesized to be the result of endogenous bacteria invading the bones shortly after death (e.g. Child, 1995; Bell et al., 1996; but see Turner-Walker, 2019), and some authors interpreted the limited extent of focal destruction as indication of quick dismemberment of the carcass, or activity of other factors halting microorganism-induced alteration of bone (Trueman & Martill, 2002). In this context, it is possible that the relatively restricted tissue destruction observed in studied specimens is the result of early *post mortem* disarticulation of the animal remains. An alternative scenario involving the fast burial of the dead body which inhibits microorganism activity within bones is deemed unlikely given all the available data (see below).

Furthermore, in 10 of the histologically sampled specimens (83.3%) there is evidence for mechanical abrasion of the bone surface (Table 1; Figs. 23, 24, 25). The combination of abraded bone surfaces and Wedl tunnels allows us to reconstruct, at least in part, the succession of events undergone by the bones before their final burial. Wherever the Wedl tunnels are sufficiently developed and can be traced laterally along the section, they appear to have a consistent length, and in places of strongly abraded bone surface, they conform to its morphology (Figs. 24a, c, d, 25b, e, f). This is clear evidence that the mechanical abrasion observed in all specimens, most probably caused by transportation, preceded the microbiotic-induced bioerosional processes affecting the bones. Once transported to the depositional center, the skeletal elements remained for an unknown amount of time on the substrate (not necessarily underwater), or partially covered by sediment. The uneven development of Wedl tunnels along the periphery of some thin sections indicates that only parts of the bone which provided a suitable nutrient and developmental environment for certain microorganisms, were infested and bioeroded.

### Macroscopic taphonomic features of the fossils

Virtually all in situ-collected macrovertebrate fossil bones with preserved extremities (epi- and metaphyses) and/or articular surfaces show a variable degree of bone abrasion/erosion in these areas (Figs. 16c, 18a, d, 19a, 20b, 21, 22). There, the thin compact cortex is partly or entirely missing and the underlying spongiosa is revealed on the surface. Bone abrasion, as evidenced by the macroscopic observation of vascular canals or cancellous bone exposed on the surface, is also observed along crests and processes (as in humeri NMNHS FR41 and 51; Figs. 16c, 18a) and the diaphyses of some bones (as in tibia NMNHS FR25; Fig. 19d). We interpret these damaged areas of the bones as the result of transportation. Because most of the taxonomically identified specimens, both in situ and ex situ collected, albeit fragmentary, are moderately complete and the indeterminate bone fragments are not rounded, it can be assumed that they did not experience prolonged transportation to the depositional basin.

Two specimens—ornithopod tibia NMNHS FR25 and putative pterosaur bone NMNHS FR39—exhibit features which present interesting taphonomic cases. Weathering (early diagenetic) cracks are present on the metaphysis and along the diaphysis of NMNHS FR25. The diaphyseal cracks are few and mostly longitudinally oriented, while those on the metaphysis are numerous and form a complex network, breaking the bone’s surface into polygons of variable size. The metaphysis is more heavily weathered on its posterior side (Fig. 19a, b). Actualistic studies have shown that the upper (exposed) surface of the bone generally experiences stronger weathering than the lower surface, while the same is true for areas of the bone which are closer to the ground than surfaces away from it (Behrensmeyer, 1978). Sometimes, the lower surface of a bone could be more weathered due to minerals crystallizing on it, under alkaline soil conditions. Either way, the repeated alternation of heating and cooling or wetting and drying wears down the bone and leads to formation of cracks (*ibid*.). Although in NMNHS FR25 some of the sediment infilling the cracks contains small gypsum crystals (CaSO_4_·2H_2_O), at present we cannot confirm if they are of early diagenetic or recent exogenic origin. In light of this, we speculate that it was the posterior side of the bone which was initially exposed to the elements, during which it suffered weathering and only at a later point the specimen was turned on its posteromedial side and filled with sediment as suggested by the other lines of evidence.

Specimen NMNHS FR39 experienced another type of taphonomic/early diagenetic alteration. The bone is clearly deformed on the one side, where the cortex is collapsed inside the medullary region, almost in contact with the opposing bone wall (Fig. 17). The deformation could be the result either of trampling of the freshly buried bone by another animal, or by structural collapse caused by the weight of overlying sediments. Whatever the reason, the breakage must have occurred before the lithification of the sediment material. This speculation seems likely because the free medullary cavity of the undeformed part of the bone is completely infilled by sediment, which also includes fragments of bivalve shells. Also, in order to compress the other side, as we observe is the case, the sediment material needs to be soft—only non-lithified sediment can accommodate for the almost complete reduction of the medullary space.

Lastly, in addition to weathering and possible trampling, a few bones, as well as some of the turtle shell fragments, show macroscopic signs of bioerosion. Specimens NMNHS FR25 and FR44 (distal tibia fragment of a titanosaur) best represent the variety of observed bioerosional traces (Figs. 19d, c, 20c, d, e). In both fossils, the marks are developed on the bone surface and/or within the upper parts of the cortex. Following the terminology of Pirrone et al. (2014), we recognize the following morphological types of traces: pits, borings (holes), channels and furrows (Fig. 19c, d). While channels occur either individually or in small groups, borings are always grouped. In the case of NMNHS FR44, the whole posterior side of the fossil is bored, with some areas having completely eroded outer cortex (Fig. 20c, d, e). The intensive bioerosion on the posterior side of the bone suggests that it was exposed and used as a substrate by boring invertebrates. Some of the elongated linear traces (channels/furrows) present on the mid-diaphysis of NMNHS FR25 can be interpreted as tooth marks, probably the result of scavenging (Fig. 19c, d) (e.g., Augustin et al., 2020). Based on the known taxonomic diversity of the assemblage, as well as on the size of the marks, crocodylomorphs appear to be the most parsimonious perpetrators responsible for such traces. Bite marks producued by Allodaposuchid-like crocodile, including linear structures similar to the ones observed in NMNHS FR25, have been previously reported from the Santonian of Hungary (Botfalvai et al., 2014). However, we cannot exclude theropods, for which there are currently no records at Vrabchov Dol but are known to be present on other islands of the European Archipelago during the Santonian and early Campanian (see Csiki-Sava et al., 2015), as possible trace makers, because of the morphological similarities of some theropod bite marks to those observed on the studied bones (e.g., Augustin et al., 2020; Robinson et al., 2015). Furthermore, because the fossils were found in brackish to marine deposits, marine vertebrates, like mosasaurs and sharks, cannot be ruled out as producers of the tooth marks either (e.g., Einarsson et al., 2010; Everhart & Ewell, 2006). The other types of bioerosional structures present in this specimen appear to be caused by the activity of invertebrate organisms. Indeed, trace fossils of similar morphology have been attributed to insects, such as termites and dermestids (Augustin et al., 2019). A detailed study focused solely on the bioerosional traces is necessary to establish the nature of the biological agents responsible for the bioerosion present in the Vrabchov Dol fossil bones. Still, macroscopic data further prove that at least some of the fossil material is characterized by a very complex taphonomic history.

## Discussion

### Palynology informed paleoenvironmental interpretation and correlation

The studied vertebrate-bearing succession at Vrabchov Dol was deposited during the latest Santonian–early Campanian, as revealed by diagnostic pollen species (e.g., *Oculopollis zaklinskaiae*, *Oculopollis orbicularis*, *Krutzschipollis crassus* and *Krutzschipollis spatiosus*). In addition to the age assessment, the overall composition of the palynomorph assemblages and palynofacies data were combined for paleoenvironmental interpretations. Palynofacies patterns in all studied samples were analyzed in order to highlight the environments of deposition. The particulate organic matter was viewed as a sedimentary component that reflects the original conditions of the source area and in the depositional environment. One palynofacies type was identified, characterizing most of the investigated samples. It is represented by a significantly high abundance of phytoclasts compared to a minor contribution of AOM and moderate one of palynomorphs. Thus, the samples plot in the palynofacies field I of the APP ternary plot of Tyson (1993), indicating proximal oxic depositional settings (Fig. 6). High abundances of translucent phytoclasts further suggest highly proximal shelf, or even oxidated deltaic or lagoonal paleoenvironments with short transportation of the continental elements. Minor marine influx from the surrounding basin was documented at two levels in the section. The encountered palynofacies is also characterized by a very high C/M ratio, again suggesting a depositional setting in proximity of a fluvio-deltaic source.

Terrestrial palynomorphs (spores and pollen) provided detailed information on continental vegetation patterns that can be linked to climate conditions (temperature and precipitation). The spore content of the samples is dominated by *Deltoidospora* spp.*, Cyathidites* spp and *Vadaszisporites* spp. These spores are considered as humidity indicators as they commonly evolved and flourished in wet biotopes under a warm and humid subtropical climate. The vegetation in the studied area was also primarily composed of a range of Normapolles—producing angiosperms attributed to the families *Sapindaceae, Juglandaceae, Myricaceae* and *Betulaceae* (Batten & Christopher, 1981; Dulić, 2002). Their climatic implication and relationship to core Fagales is well established (Friis et al., 2011). In general, the group is thought to have been wind-pollinated and that Normapolles-producing plants grew under warm, seasonably dry climate. Thus, the vegetation pattern reflects a humid subtropical, seasonally dry climate during the latest Santonian–early Campanian in the studied area. The presence of bothremydid turtles and eusuchians in the vertebrate assemblage is indicative of a warm climate and supports this conclusion (Mannion et al., 2015; Markwick, 1998; Pérez-García & Rubio, 2024, p. 5, and references therein).

Palynology may be used to correlate the latest Santonian–early Campanian vertebrate site at Vrabchov Dol with the other well-known similarly aged sites in Hungary and Austria, by relying on biostratigraphy, Normapolles species diversity and abundance, as well as palynofacies characteristics and depositional environments. All these sites fall within the Late Cretaceous Normapolles microfloristic province defined by Góczán et al. (1967), featuring the restricted spatial distribution of most representatives to Europe and eastern North America. The presence of different Normapolles taxa in the assemblages from the host strata, with their high diversity and rapid evolution during the Santonian and Campanian, makes this group a prime biostratigraphic marker for correlating the vertebrate and dinosaur bearing sites in Bulgaria (Vrabchov Dol), Hungary (Iharkút and Ajka) and Austria (Muthmannsdorf).

Bodor and Baranyi (2012) reported rich Santonian palynological assemblages from the Iharkút vertebrate site, Bakony Mountains in Hungary. The palynological content of Iharkút corresponds well to those from Vrabchov Dol by its similar genera and species diversity, the Normapolles dominance and the most profuse *Oculopollis* and *Krutzschipollis* representatives (e.g., *Oculopollis zaklinskaiae*, *Oculopollis orbicularis*, *Krutzschipollis crassus*, *Krutzschipollis spatiosus* and *Krutzschipollis magnoporus*). Both localities have more than 90 percent Normapolles genera and species in common considering their palynofloras. Only the genus *Hungaropollis* diversified during the Campanian in Hungary (Góczán, 1964) and the Gosau Basin (Pavlishina et al., 2004) and is not found in the Bulgarian site. In general, the Bulgarian and Hungarian sites share common markers such as similar Normapolles assemblages, fluvial and deltaic environments as well as paleoclimatic reconstructions suggesting humid subtropical, seasonally dry climate.

The Muthmannsdorf site is part of the Grünbach Formation within the Gosau Group in the Grünbach—Neue Welt Basin in Austria. Draxler (in Summesberger, 1997) identified 29 species of fern, gymnosperm and angiosperm spores and pollen from the Grünbach Formation in this basin and emphasized that the most characteristic element from the early Campanian Grünbach palynoflora is the pollen from the Normapolles group. Hradecká et al. (1999) also reported comparatively diverse Normapolles assemblages from this formation in which angiosperm pollen comprises 80% of the total assemblage, with dominance of the *Oculopollis, Suemegipollis* and *Trudopollis* species in it. According to the studies of Herman and Kvaček (2007), the Grünbach flora experienced a humid sub-tropical climate with warm/hot summers and short relatively dry seasons. The dominating paleogeographic environment suggested for the plant-bearing deposits of the Grünbach Formation was that of a large island with unknown relief, at least temporarily connected to the continent. The terrestrial flora and the shallow water sediments also indicated a relatively large deltaic plain. In conclusion, the Bulgarian and Austrian sites share common markers such as similar Normapolles assemblages, continental and deltaic environments, and paleoclimatic reconstructions suggesting a humid subtropical, seasonally dry climate.

Following the sum of data, we can conclude that Normapolles pollen is a key indicator of floristic changes during the Late Cretaceous. This pollen, associated with the development of angiosperms, has been found in all three regions with similar abundance and diversity and reveals common ecological and evolutionary patterns during the Sanonian–Campanian in the Eastern and Central European Archipelago islands.

### Paleoecology and taphonomy of Vrabchov Dol’s fossil vertebrates

Our palynofacies analyses indicate that the vertebrate fossils found at the Vrabchov Dol locality were buried in a coastal environment with a strong fluvial influence, possibly a lagoon or the foreshore parts of a marine basin (Pavlishina et al., 2019; this study). The contents of the vertebrate fossil assemblage reflect the nature of such depositional environment: we find a combination of aquatic (fish), semi-aquatic (amphibians, turtles and crocodylomorphs) and terrestrial (non-avian dinosaurs and possibly pterosaurs) taxa, with water-dwelling vertebrates covering the whole spectrum of freshwater (lepisosteids and amphibians) to saline-tolerant (lepisosteids, turtles and crocodylomorphs) and marine (lamniform sharks) elements. The nature of the depositional environment is also reflected in the relative abundance of animals bound to water habitats, which constitute about 2/3 of all collected vertebrate fossils (Fig. 13). Turtle fossils, which are almost exclusively partially preserved shells and shell fragments, are most abundant among the recognized vertebrate groups (41.36% of the taxonomically identified material; Fig. 13), as is also the case in some other Upper Cretaceous European sites (e.g. Botfalvai et al., 2015; Buffetaut et al., 1999; Pereda-Suberbiola et al., 2000). Upward of bed 7 there is a noticeable increase in the abundance of vertebrate fossils, with 77.51% (n = 124) of stratigraphically well constrained specimens coming from this part of the section. It can be speculated that the increase reflects changes in the environment of and around the depositional basin (such as sea level changes), favoring the accumulation of more remains of terrestrial and freshwater vertebrates. It is worth mentioning that a change for this interval is also observed in our palynological data, where there is a drop in palynomorph abundance and taxonomic diversity (Fig. 4), although, at present, we cannot connect it to the interpretation inferred from the vertebrate record.

Body size of taxa in the assemblage varies greatly, from a few centimeters for lissamphibians to likely several meters for ornithopods and titanosaurs. Our data shows that microvertebrate fossils (including teeth) account for 41.2% of all vertebrate remains. Although we have found teeth, scales, and amphibian limb bones in situ, the relative abundance we report is probably an underestimation of the actual microvertebrate abundance, because of the limited amount of sediment material which was screenwashed and examined. Despite the potential sampling bias, acknowledged by us earlier, we can conclude that the fossil density of vertebrate macrofossils for all fossil-bearing strata is low.

A common feature of both the microvertebrate and macrofauna material is that the skeletal elements are isolated and usually incomplete. No partial skeletons or articulated remains have been found to date. Curiously, cranial elements other than isolated teeth appear to be completely absent from the assemblage. The low number of vertebrae (only 5.6% of all specimens; Fig. 11) and the abundance of dermal elements (31.6%), long bones (25.6%), and teeth (15.2%) might suggest a case of a lag deposit with lighter, more floatation-prone skeletal elements, like vertebrae and ribs, hydraulically sorted out of the assemblage, leaving bones less susceptible to transportation in place (Behrensmeyer, 2003; Voorhies, 1969). Alternatively, the lack of certain skeletal elements in our sample could indicate that these elements did not survive the transportation from freshwater or terrestrial environments, located away from the depositional center. Similarly, the absences of juvenile skeletal remains, at least as revealed by our osteohistological data, could be explained with reference to their more fragile nature compared to more mature bones, which prevents them surviving prolonged transport by water currents.

The predominance of generally durable elements, like dermal bones, scales and teeth, along with the lack of cranial material, which is considered least susceptible to transportation (see Voorhies, 1969), and thus is expected to be present in a lag deposit, and long bones and vertebrae with macroscopic signs of mechanical abrasion all leads to accept the second scenario of a highly transported assemblage as more likely. Furthermore, although some long bones, for which we have better field data, seem to show some weak spatial orientation, fossils are not sorted by size, with microfossils and large bones found in the same stratum and occasionally even together. (One example is a partial tibiofibula, which was found in the matrix enclosing titanosaur fibula NMNHS FR45. The specimens are shown on Figs. 14i and 21a, respectively.) The sum of all presented data reveals the Vrabchov Dol locality as an example of an attritional fossil assemblage in a coastal environment, with incremental accumulation of para-autochthonous and allochthonous elements, representative for the vertebrate fauna inhabiting various inland and coastal habitats.

From the body size discrepancy of different vertebrates present at the locality alone, it is clear that the fossils of microvertebrates (including teeth and lepisosteid scales) and macrofauna (dinosaurs in particular), despite being found in the same strata, had very different taphonomic histories. In the case of the fragile millimeters-long limb bones of lissamphibians, the transport from freshwater areas must have been very short, so that these tiny elements were able to remain coherent and fossilize. The material from macrofauna, however, presents a much more complicated story. All dinosaur (and a putative pterosaur) vertebrae and long bones with sufficiently preserved extremities show signs of abrasion in these areas (Figs. 16, 17, 18, 19, 20, 21, 22). Furthermore, cortical abrasion at a microscale level has been confirmed for 10 of the 12 histologically sampled specimens (Table 1; Figs. 23, 24, 25). (The other two fossils could not be evaluated either because of their fragmentary nature [NMNHS FR19], or because of cortical destruction in the process of thin section preparation [U.S., K2 1586].) We interpret the ubiquitous presence of damaged surfaces on the bones as evidence for transportation of the material to the site of deposition. Certainly, the distance which each bone traveled was different, but with the evidence at hand, it is not possible to evaluate how far each bone was transported. An allochthonous origin for the material is additionally supported by the isolated and commonly fragmented nature of the fossil bones.

Besides their allochthonous origin, some dinosaur remains share another common taphonomic feature, namely the presence of Wedl tunnels (Hackett, 1981; Wedl, 1864). These biogenic structures seem to be the result of the activity of endolithic microorganisms, like cyanobacteria and/or algae (Davis, 1997; Turner-Walker, 2019), although fungi have also been suggested as bioerosional agents (Hackett, 1981; Marchiafava et al., 1974). Experimental data show that in freshwater and shallow marine environments microbial bioerosion of bone starts as soon as suitable environmental conditions are met and progresses quickly until the remains are buried (Davis, 1997). The observed Wedl tunnels in most sampled bones indicate that these skeletal elements remained on the substrate where they were invaded by endogenic flora. As inferred from the relationships between the bone surfaces and the Wedl tunnels (Figs. 23, 24, 25), the microbial bioerosion postdates the transportation of the specimens to the depositional basin.

Non-Wedl tunnels are another type of microbial bioerosion commonly observed in fresh (recent) and fossil bone (Hackett, 1981). The structures are thought to be caused by bacteria soon after death, although the exact source of these organisms, i.e. soil or gut, remains elusive (Bell et al., 1996; Child, 1995; Jans, 2022; Turner-Walker, 2019). If endogenous bacteria are indeed responsible for the quick post mortem degradation of bone, like suggested by Bell et al. (1996) (for nuanced discussion see Turner-Walker, 2019), then the preservation of bone in its fossil state would imply timely halting of the bone destructive processes, either by dismembering of the carcass or by its fast burial (Trueman & Martill, 2002). The bones we studied histologically show mostly limited microscopic tissue destruction of non-Wedl type. Following the reasoning outlined above, we can hypothesize that the lack of extensive microscopic bone alteration in most sampled specimens is evidence that the carcasses did not remain in their natural state for long, possibly due to the activity of various scavengers. Interestingly, at least one specimen (NMNHS FR25) exhibits macroscopic traces, which can tentatively be interpreted as bite marks resulting from scavenging (Fig. 19c, d).

Macroscopic signs of bioerosion present in part of the dinosaur material also support the exposure of some bones on the substrate. We observed several types of bioerosional traces on bone surfaces, including pits, borings and meandering channels or furrows (Figs. 19c, 21d, e) (Pirrone et al., 2014). Pits and borings, in particular, are numerous, especially in NMNHS FR44 in which the whole preserved posterior bone surface is affected. In the literature, trace fossils of similar morphology to those observed on the Vrabchov Dol bones are attributed to insects, like dermestid beetles and termites (e.g., Augustin et al., 2019; Bader et al., 2009; Britt et al., 2008; Perea et al., 2025). To elucidate the nature of the invertebrates which eroded the examined bones, which is beyond the scope of this contribution, a detailed study on the morphology and structure of the trace fossils is necessary. In addition to the invertebrate damage, the noticeably weathered metaphyseal surface of ornithopod tibia NMNHS FR25 (Fig. 19b) suggests repeated cycles of wetting and drying or heating and cooling, which can happen only if the bone remained on the substrate for certain amount of time. Lastly, specimen NMNHS FR39 is damaged, either as a result of a trampling by a large vertebrate or due to bone collapse caused by the weight of overlying sediment.

In conclusion, we postulate that the fossil remains of large vertebrates found at Vrabchov Dol each have unique and complex taphonomic history unified by a process of transportation to the site of deposition and subsequent micro- and/or macroscopic bioerosion.

### General comparisons with the fauna of similarly aged and some younger European localities

There are relatively few known Santonian and lower Campanian fossil vertebrate localities in Europe which yield remains of terrestrial and freshwater fauna (Csiki-Sava et al., 2015). Some of these localities have produced scarce and fragmentary material (Averianov, 2014; Buffetaut et al., 2002b; Godefroit & Lambert, 2007; Lindgren et al., 2007), while others record more diverse fauna, either in the form of skeletal remains or as ichnofossils (Buffetaut et al., 1996; Chiarenza et al., 2021; Nicosia et al., 1999a, b). The Santonian vertebrate fauna of Hungary (Botfalvai et al., 2021b; Ősi et al., 2012b, 2016) and the early Campanian vertebrates of Austria are among the best studied on the continent (Bunzel, 1870, 1871; Seeley, 1881; Csiki-Sava et al., 2015), because of their richness, and in the case of the Austrian material, its long research history. The European fossil record of terrestrial vertebrates becomes significantly more complete in the upper Campanian and the Maastricthian, particularly for the territories which were located in the more eastern parts of the European Archipelago (Csiki-Sava et al., 2015; and references therein). These territories were then part of the so-called Hațeg Island, nowadays western Romania, and home to peculiar vertebrate faunas with dwarfed dinosaurs, abberant theropods and giant pterosaurs (Benton et al., 2010; Buffetaut et al., 2002a; Csiki et al., 2010c; Weishampel et al., 2010). Traditionally considered all Maastrichtian in age, recently, some of the Romanian vertebrate assemblages have been re-interpreted as upper Campanian (Albert et al., 2025; Bălc et al., 2024; Ebner et al., 2025). These new age estimations reduce the temporal gap between the oldest representatives of the Hațeg Island faunas and the earlier Santonian to lower Campanian vertebrate faunas of Hungary, Austria, and Bulgaria and open the way for new interpretations and testing of paleobiogeographical scenarios. The most recent addition to the Upper Cretaceous vertebrate fossil record of Europe is the Maastrichtian Osmakovo locality in southeastern Serbia. It yields surprisingly diverse fossil assemblage, including non-avian dinosaurs and mammals, and exhibits similarities to contemporaneous Romanian assemblages (Marković et al., 2025). Currently, Osmakovo is (paleo)geographically the closest Upper Cretaceous locality to Vrabchov Dol.

The Vrabchov Dol locality is unique for the Balkan Peninsula (particularly its eastern to southeastern parts), not only for yielding fossils of vertebrates from at least seven different clades but also for providing information about the taxonomic composition of the terrestrial fauna in a currently unexplored part of the Late Cretaceous European Archipelago from a time interval for which the fossil record of Europe is still relatively incomplete. To put its taxonomic contents into a wider paleogeographic, stratigraphic and evolutionary perspective, a comparison with the faunas of the geographically nearest fossil localities of similar Santonian–Campanian but also younger age in Europe, which, in this case, are those from Hungary, Austria, Romania, and Serbia, is warranted.

The first continental vertebrate remains from the Santonian in Hungary were found in 2000, in the alluvial floodplain deposits of the Csehbánya Formation at Iharkút (Ősi, 2004). After two decades of field work and over 100,000 collected specimens, the Iharkút locality is now one of the most important Upper Cretaceous vertebrate localities in Europe and provides an unprecedented look into the life and evolution of the terrestrial fauna on the European Archipelago during the mid-Late Cretaceous (Botfalvai et al., 2021b; Ősi et al., 2012b). The vertebrate assemblage is taxonomically and paleoecologically diverse, including over 40 taxa with terrestrial, semi-aquatic and aquatic modes of life (Botfalvai et al., 2021b). These include lepisosteid gars (Szabó & Ősi, 2017; Szabó et al., 2016a), pycnodontids (Szabó & Ősi, 2017; Szabó et al., 2016b), amiid, elopiform, ellimmichthyiform, salmoniform and acanthomorph actinopterygians (Szabó & Ősi, 2017), albanerpetonids (Szentesi et al., 2013), representatives of several anuran clades (Szentesi & Venczel, 2010, 2012), seven scincomorph and one mosasauroid lizards (Makádi & Nydam, 2015; Makádi et al., 2012; Makádi, 2006, 2013a, 2013b), ‘kallokibotionid’, dortokid and bothremydid turtles (Rabi & Botfalvai, 2006; Rabi et al., 2012, 2013), four crocodylomorphs, including the genera *Doratodon* and *Iharkutosuchus* (Ősi et al., 2007, 2012b; Ősi, 2008a; Rabi & Sebők, 2015), at least two azhdarchid pterosaurs (Ősi et al., 2005, 2011; Prondvai et al., 2014a), two species of nodosaurid ankylosaurs (Ősi, 2005; Ősi & Makádi, 2009; Ősi & Prondvai, 2013), the rhabdodontid *Mochlodon vorosi* (Ősi et al., 2012a), the ceratopsian *Ajkaceratops kozmai* (Ősi et al., 2010b; but see Czepiński & Madzia, 2024), titanosauriform sauropod (Ősi et al., 2017), and abelisaurid, basal tetanuran, non-avian maniraptoran and enantiornithean theropods (Dyke & Ősi, 2010; Ősi & Buffetaut, 2011; Ősi et al., 2010a; Ősi, 2008b). Botfalvai et al. (2015) established the depositional environment for the vertebrate assemblage as a floodplain of a low-gradient river. These authors also recognized three subsets within the assemblage, each with its own taphonomic history, with fossils of aquatic and semi-aquatic animals being the most common in general (*ibid.*). The lithological characteristics of the fossil-bearing succession at Iharkút reveal that the richest fossil layers represent a lag deposit, which formed in a short period of time as a result of flooding events (Botfalvai et al., 2016). Recently, Ősi et al. (2016) described a vertebrate assemblage from the neighbouring contemporaneous Ajka Coal Formation. This fauna, albeit known from more limited and fragmentary material, appears to show the same overall taxonomic composition as that of the Csehbánya Formation. However, the freshwater to brackish deposits of the Ajka Coal Formation were formed in swampy lacustrine environment (Ősi et al., 2016).

Recently, the sedimentary successions of the Gosau Group in Austria produced two new vertebrate assemblages of late Turonian and early Coniacian age, respectively (Ősi et al., 2019b, 2021). These freshwater to brackish and marine assemblages, albeit comprised of the fragmentary remains of fishes (including lepisosteids), amphibians, mosasauroids, other lacertilians, testudines, neosuchians and theropod dinosaurs, suggest a tantalizing connections with the younger Santonian vertebrate fossil fauna of Hungary, and by extension – the early Campanian one of Austria (Ősi et al., 2019b, 2021).

Unlike the Santonian vertebrate assemblages in Hungary, the sauropsid fauna from the lower Campanian of Muthmannsdorf, eastern Austria, has been known since the 1870s (Bunzel, 1870, 1871; Seeley, 1881). The fossiliferous strata are part of the coal-bearing Grünbach Formation, a unit deposited in freshwater to shallow marine environments (Summesberger et al., 2000, 2007). Regrettably, after the cessation of coal mining activity at Muthmannsdorf in the end of nineteenth century, which initially had led to the discovery of most of the fossils (Seeley, 1881), no additional vertebrate remains have been collected there (Csiki-Sava et al., 2015). For over a century, the fossils from this locality have been one of the most important sources of information about the European Late Cretaceous vertebrate faunas, and as such, it has attracted sufficient scientific interest. Although the fossil assemblage is nowhere near as rich as that from Iharkút it includes many of the taxa typical for the Late Cretaceous European Archipelago, such as ‘kallokibotionid’ and dortokid turtles (Rabi et al., 2013; Seeley, 1881), crocodyliforms, including *Doratodon* (Buffetaut, 1979; Rabi & Sebők, 2015; Seeley, 1881), azhdarchid pterosaurs (Buffetaut et al., 2011; Wellnhofer, 1980), the nodosaurid *Struthiosaurus austriacus* (Bunzel, 1870; Pereda-Suberbiola & Galton, 1992, 1994, 2001; Seeley, 1881; Stumpf et al., 2023), the rhabdodontid *Mochlodon suessi* (Bunzel, 1871; Ősi et al., 2012a; Sachs & Hornung, 2006; Seeley, 1881) and non-avian theropods (Ősi et al., 2010a; Seeley, 1881), as well as some more ‘exotic’ elements, like choristoderes (Buffetaut, 1989). Because the locality has not been explored further, especially for the presence of microvertebrate fossils, it can be assumed that the known list of taxa underrepresents the actual taxonomic diversity at Muthmannsdorf.

Similarly to the Muthmannsdorf vertebrate assemblage, the uppermost Cretaceous fossil vertebrates of western Romania have been known for well over a century and have enjoyed increased scientific interest in the past 50 years (e.g., Csiki-Sava et al., 2015, 2016; Grigorescu, 1983, 2005, 2010; Nopcsa, 1900, 1914, 1923). In result, numerous vertebrate-bearing localities are currently known from the Haţeg, Transylvanian, and Rusca Montană basins (e.g., Botfalvai et al., 2021a; Codrea et al., 2010, 2012; Vasile & Csiki, 2011; Vremir, 2010). Despite spanning several million years, from the late Campanian to the late Maastrichtian, the Haţeg Island faunas show generally static taxonomic composition (Csiki-Sava et al., 2016). In general, the vertebrate fossil assemblages of uppermost Cretaceous Romania are comprised of osteichthyans, including lepisosteids (e.g., Codrea & Jipa, 2011; Csiki et al., 2008; Trif & Codrea, 2022), anuran and albanerpetontid amphibians (e.g., Folie & Codrea, 2005; Vasile et al., 2019; Venczel et al., 2016), diverse squamates (e.g., Folie & Codrea, 2005; Vasile et al., 2013, 2019; Venczel & Codrea, 2016), kallokibotionin and dortokid turtles (Augustin et al., 2021; Nopcsa, 1923; Pérez-García & Codrea, 2018; Rabi et al., 2013), ziphosuchian, atoposaurid, allodaposuchid and hylaeochampsid crocodyliforms (Martin et al., 2006, 2010; Narváez et al., 2020; Venczel & Codrea, 2019), azhdarchid pterosaurs, including two nominal taxa (Buffetaut et al., 2002a; Vremir et al., 2013, 2015), nodosaurids (e.g., Codrea et al., 2010; Ősi et al., 2014), rhabdodontid ornithopods (Augustin et al., 2022; Brusatte et al., 2017; Godefroit et al., 2009; Magyar et al., 2024), hadrosauroids (Ebner et al., 2025; Grigorescu & Csiki, 2006; Nopcsa, 1900; Weishampel et al., 1993), at least four genera of titanosaurian sauropods (Csiki et al., 2010a; Díez Díaz et al., 2025; Mocho et al., 2023, 2024), non-avian theropods (e.g., Csiki & Grigorescu, 1998; Csiki et al., 2010c, 2015; and references therein), birds (Dyke et al., 2012; Wang et al., 2011a, b), and kogaionid multituberculate mammals (e.g., Codrea et al., 2014, 2017; Csiki & Grigorescu, 2001; Csiki-Sava et al., 2022).

Although the depositional environment of the Vrabchov Dol assemblage is more akin to that of the contemporaneous Muthmannsdorf and the Turonian and Coniacian vertebrate localities of Austria (Ősi et al., 2019b, 2021; Summesberger et al., 2000, 2007), at a higher taxonomic level its content shares more similarities with the slightly older Iharkút and Ajka faunas, as well as with the late Campanian–Maastrichtian ones of the Hateg Island. Shared faunistic elements with the Hungarian assemblages include lepisosteids, amphibians, bothremydid turtles, allodaposuchids, hylaeochampsids, ornithopods, sauropods and possibly pterosaurs, while similarities to uppermost Cretaceous Romanian localities include the presence of lepisosteids, amphibians, allodaposuchids, hylaeochampsids, ornithopods (hadrosauroids in particular), titanosaurs, and, again, possibly pterosaurs. Curiously, the dinosaur fauna of Vrabchov Dol appears to be more similar in composition and abundance of taxa (see below) to that of Romania (and also Serbia; see Marković et al., 2025), than to those of Hungary and Austria.

Although ongoing work on the osteological description and taxonomic evaluation of most of the Bulgarian material precludes precise correlation and discussion of paleobiogeographic hypotheses, some observations and comments can be made. Nikolov et al. (2025) noted the similarities in apical morphology of indeterminate lepisosteid teeth from Vrabchov Dol to dental remains attributed to *Atractosteus* from the Santonian of Hungary (Ősi et al., 2016; Szabó et al., 2016a). Lanceolate lepisosteid teeth from some Maastrichtian localities in Romania have also been ascribed to *Atractosteus* solely on the basis of tooth morphology, although, regrettably, none of these specimens is illustrated in the literature (Codrea & Jipa, 2011; Csiki et al., 2008). In Europe, sans Cenomanian finds from the Iberian Peninsula (e.g., Pérez-García & Rubio, 2024; Pérez-García et al., 2017), pre-late Campanian bothremydid turtles are currently known only from France, Hungary and Bulgaria (Nikolov et al., 2020a; Rabi & Botfalvai, 2006; Rabi et al., 2012; Tong & Gaffney, 2000), which places the Bulgarian material among the oldest on the continent. One of the characteristics of the uppermost Cretaceous fossil record of Romania is the absence of bothremydid pleurodirans (Csiki-Sava et al., 2015; Rabi et al., 2013). Vremir (2004) mentions a specimen possibly referrable to the bothremydid genus *Polysternorn*, but this record has not been confirmed by future studies (e.g., Rabi et al., 2013). Similar to bothremydid turtles, the presence of allodaposuchids in the Bulgarian assemblage is another element shared with Hungarian and western European faunas, but not with the early Campanian Austrian one. The oldest remains from these basal eusuchian crocodylomorphs come from the Santonian of Hungary and the lower Campanian of France (Csiki-Sava et al., 2015; Martin & Buffetaut, 2008; Ősi et al., 2016). The isolated teeth from Vrabchov Dol ascribed to Allodaposuchidae extend the known paleobiogeographic range of the clade to the more eastern portion of the European Archipelago at the onset of the Campanian (Hristova, 2020). Allodaposuchids are common element in many of the Romanian localities (Csiki-Sava et al., 2016). Hylaeochampsids, the other eusuchians possibly present in the Vrabchov Dol assemblage, are part of the crocodyliform fauna of both the Iharkút–Ajka and the Hateg Island faunas (Csiki-Sava et al., 2015, 2016; Ősi et al., 2012b).

In the context of the European realm, the landmasses which were home to the Iharkút and Vrabchov Dol faunas are notable in having been inhabited by sauropods in Santonian-early Campanian times, as evidenced by body fossils (Nikolov et al., 2020b; Ősi et al., 2017; this study). Similarly-aged sauropod ichnites are known from the Apulian Platform (Nicosia et al., 1999a). Both the Hungarian and Bulgarian fossil material argue against the hypothesis of Le Loeuff (1993) for a Late Cretaceous “sauropod hiatus” in Europe and promise intriguing paleogeographic implications once more complete and diagnostic fossils are found. However, sauropods appear to be extremely rare in Iharkút, with just one known specimen after two decades of excavational work (Ősi et al., 2017). This is not the case with Vrabchov Dol, where titanosaur remains comprise 20% of the taxonomically determined dinosaur specimens (for comparison, the ornithopod specimens are 15.56% of all dinosaur specimens). Similarly, in the uppermost Cretaceous localities in Romania titanosaur sauropods are among the most common dinosaurs and also exhibit high taxonomic diversity (Csiki-Sava et al., 2015; Díez Díaz et al., 2025). The Maastrichtian Osmakovo locality in Serbia, which is the closest one to Vrabchov Dol reported so far, also yields sauropod remains (Marković et al., 2025). It seems certain that sauropods were consistently present and even abundant in the region since at least the latest Santonian.

Although sauropods remain rare during the Santonian–early Campanian interval in Europe, this is not the case with other herbivorous dinosaur clades like nodosaurid ankylosaurs and ornithopods, the latter of which are represented by rhabdodontids, and less commonly by hadrosauroids (Augustin et al., 2023b; Chiarenza et al., 2021; Csiki-Sava et al., 2015). Based on currently available evidence, nodosaurids are absent from the Vrabchov Dol vertebrate assemblage. This is somewhat unexpected considering the inferred paleoecology and depositional environment of the studied locality and the hypothesized preference of coastal and/or lowlands environments for nodosaurids (Butler & Barret, 2008; Arbour et al., 2016; Vázquez López et al., 2025). The lack of fossils from these dinosaurs could be either the result of sampling and/or preservational bias or reflection of their actual absence on this landmass. The latter is an intriguing possibility but to gain support it requires the discovery of more vertebrate material at Vrabchov Dol which can provide a more robust statistical framework for data analysis. It is worth noting that while nodosaurids are the most commonly found non-avian dinosaurs in Iharkút, they are very rare in the uppermost Cretaceous vertebrate assemblages of Romania (Botfalvai et al., 2015; Csiki-Sava et al., 2015; Ősi et al., 2019a). Currently, ornithopods are the only ornithischians present at the studied locality. At least two of the collected fossils can be tentatively referred to Hadrosauroidea, a group which is poorly represented in the uppermost Santonian–lowermost Campanian of Europe (Chiarenza et al., 2021; Dalla Vecchia, 2009). Because these remains are yet to be described and compared in detail to other European taxa, we cannot provide a meaningful commentary on their importance for the evolution and paleobiogeography of hadrosauroids during the latter half of the Late Cretaceous on the European Archipelago. The same applies to the other ornithopod fossils found at Vrabchov Dol, which await a more detailed study. However, regardless of whether all of the Bulgarian material pertains to hadrosauroids, or if part of it belongs to rhabdodontids, it is certain that the fossils belong to individuals which were more similar in size to animals like *Zalmoxes*, *Telmatosaurus*, or young individuals of *Tethyshadros*, than to the smaller Santonian and early Campanian species of *Mochlodon* (Augustin et al., 2023b; Chiarenza et al., 2021; Ősi et al., 2012a). Lastly, if specimen NMNHS FR39 is confirmed to be of azhdarchid nature, then this would imply the presence of a pterosaur much larger than Hungarian and Austrian taxa during the Santonian to early Campanian of Europe (Buffetaut et al., 2011; Ősi et al., 2005, 2011; Prondvai et al., 2014a), one that is closer in size to the giants of latest Cretaceous Romania (Buffetaut et al., 2002a).

In conclusion, the current state of knowledge suggests that the Bulgarian vertebrate fauna shares more similarities to the Santonian fauna of Hungary, and to some degree to that of western Europe (France), than to the contemporaneous fauna of Muthmannsdorf, Austria. However, the herbivorous dinosaur fauna of Vrabchov Dol is more similar to that of the latest Cretaceous of Romania (and to lesser degree to that of Serbia, although this is probably due to the still limited dinosaur material from Osmakovo), than to that of Iharkút and Ajka in Hungary. The similarities include an abundance of titanosaurs, presence of hadrosauroids, and rarity/absence of nodosaurid ankylosaurs. These can be explained with the proximity of the Bulgarian locality to the vertebrate-bearing uppermost Cretaceous sedimentary basins of Romania, which back in the Late Cretaceous were much closer to one another and seem to have been part of the same island chain (Dercourt et al., 2000; van Hinsbergen et al., 2020). The shared paleogeographic history of these landmasses probably facilitated the migration of terrestrial vertebrates between islands. It is possible that during the latest Santonian and earliest Campanian the ancestors of some of the well-known inhabitants of the Hațeg Island, like titanosaurs and hadrosauroids, lived on the landmass, which remnants crop out in modern-day western Bulgaria. Still, more anatomical work and additional fossils are necessary to further evaluate the place of the Vrabchov Dol vertebrates in the context of the European Archipelago’s terrestrial ecosystems and the relationships of this fauna with better known quasi-contemporaneous and later Campanian–Maastrichtian faunas from different parts of the continent, particularly those from Romania and Serbia.

## Conclusions

The fossil vertebrate locality at Vrabchov Dol offers a rare view of life on land during the latest Santonian–early Campanian times in currently understudied parts of the Late Cretaceous European Archipelago. Our multidisciplinary investigations revealed new information about aspects of the paleoecology and taphonomy of this important locality. Bed by bed study of the palynological content of the fossil-bearing section shows that the paleoflora in the studied area was dominated by angiosperms from the Normapolles group, with subordinate presence of ferns and rare gymnosperms. The presence of spore taxa *Deltoidospora* spp., *Cyathidites* spp. and *Vadaszisporites* spp., which are humid indicators throughout the sedimentary section, as well as the general composition of the flora, reflect a subtropical humid, seasonally dry climate during time of deposition. The established *Krutzschipollis crassus*—*Krutzschipollis spatiosus* Association assigns a latest Santonian–early Campanian age for the vertebrate assemblage. Our palynofacies analysis indicates deposition in coastal, proximal shelf to oxidated deltaic or lagoonal paleoenvironment with short transportation of the continental elements, which is in agreement with earlier interpretations of the depositional environment. A minor marine influx in two levels of the section (beds 3 and 7) is marked by the rare occurrence of dinocysts.

The vertebrate assemblage is dominated by turtles whose remains (partial shells and shell fragments) comprise over 30% of all vertebrate fossils found at the site. Non-avian dinosaurs are the second most abundant group, followed by crocodylomorphs and amphibians. In total, representatives of at least seven vertebrate clades are present. Most of these are aquatic to semi-aquatic freshwater or brackish-water tolerant groups, like amphibians, lepisosteids, turtles and crocodylomorphs. The remains of strictly terrestrial vertebrates account for 18% of the assemblage. The skeletal elements are disarticulated, isolated and, most commonly, fragmentary. Turtle shells and shell fragments are the most common type of vertebrate fossil found at Vrabchov Dol, with long bones being second. No cranial elements have been found so far. Long bones appear to show some degree of spatial orientation but more robust field data is necessary to confirm these observations. Fossil bones are not sorted by size, with microvertebrate and macrofauna remains found in the same strata and in close proximity. All sufficiently preserved specimens show signs of abrasion on the bone extremities, with some exhibiting signs of weathering. These indicate transportation of the material and subsequent exposure on the surface before final burial. The latter is supported by the observed microscopic (in paleohistological thin-sections) and macroscopic bioerosional marks, caused, respectively, by endolithic flora, like cyanobacteria, and invertebrates and possibly scavengers. Additionally, our histological data shows that among the sampled dinosaur bones there is no juvenile material, with most elements coming from subadult individuals, some of which approach skeletal maturity. The sum of available evidence suggests that the studied vertebrate assemblage is of attritional type, with fossils of mostly para-autochthonous but also allochthonous nature accumulating in the depositional basin over a period of time during which many of them remain on the substrate under subaerial or subaquatic conditions.

Comparisons of the known taxonomic composition of the Vrabchov Dol assemblage with that of other European localities of similar age, and particularly the Santonian Iharkút and Ajka in Hungary and the lower Campanian Muthmannsdorf in Austria, show that the Bulgarian vertebrate fauna is most similar in composition to the slightly older Hungarian one. Shared components of potential paleobiogeographic importance include lepisosteid gars, bothremydid turtles, allodaposuchids and sauropods. Uniquely, at least for the moment, is the absence of nodosaurids in Vrabchov Dol, dinosaurs which are otherwise a common component of Late Cretaceous dinosaur communities across the European Archipelago. Further comparisons with the younger (late Campanian–Maastrichtian) vertebrate faunas of Romania and Serbia reveal some shared characteristics of the dinosaur assemblages of Vrabchov Dol and the Hațeg Island. Most notable is the common presence of titanosaurs and ornithopods, hadrosauroids in particular, as well as the rarity (or absence) of nodosaurids. Other shared vertebrate groups include lepisosteids, amphibians, and allodaposuchid and hylaeochampsid crocodyliforms. Notable difference between the Bulgarian and Romanian assemblages is the absence of bothremydid turtles in the latter.

Future geological, sedimentological, geochemical, and mineralogical studies of the fossil-bearing strata and part of the vertebrate material will doubtlessly build upon the results and interpretations presented herein, further expanding our knowledge on the depositional environment, paleoecology and taphonomy of the Vrabchov Dol locality. Lastly, more fossil material and more precise taxonomic identification of the collected material is necessary for the better understanding of the role of the latest Santonian–early Campanian vertebrate fauna of Bulgaria for the evolution of the European terrestrial vertebrate ecosystems leading up to the mass-extinction at the K-Pg boundary.

## Supplementary Information

Supplementary file 1.

Supplementary file 2.

Supplementary file 3.

Supplementary file 4.

## Data Availability

All data used in this study is provided as supplementary files (Tables S1-S4). Information on the precise geolocation of the vertebrate locality can be provided to professional paleontologists and geoscientists for research purposes which do not involve field work at the site upon request sent to the corresponding author. Paleontological material is housed at the National Museum of Natural History at the Bulgarian Academy of Science (NMNHS), Sofia, Bulgaria and palynological slides are curated by P.P. at the Department of Geology, Paleontology and Fossil Fuels at Sofia University, Sofia, Bulgaria. Because of the ongoing research and descriptive work of the fossil material subject of this contribution, unpublished information regarding the specimens can be provided on a case by case basis upon request sent to the corresponding author or to Assoc. Prof. Latinka Hristova, curator of the non-mammalian paleontology collection at NMNHS.
